# Structural and kinetic mechanisms of state-dependent potassium channel inhibition

**DOI:** 10.1126/sciadv.aef8456

**Published:** 2026-10-02

**Authors:** Efthimios Kyriakis, Agnese Roscioni, Jodene Eldstrom, Daniel Sastre, Ying Dou, Alice Taddei, Steven Molinarolo, Michael Tian, Luca Maragliano, Filip Van Petegem, David Fedida

**Affiliations:** ^1^ The Department of Anesthesiology, Pharmacology and Therapeutics, University of British Columbia, Vancouver V6T 1Z3, Canada.; ^2^ Department of Life and Environmental Sciences, Polytechnic University of Marche, Via Brecce Bianche, Ancona 60131, Italy.; ^3^ Center for Synaptic Neuroscience and Technology, Istituto Italiano di Tecnologia, Largo Rosanna Benzi 10, Genova 16132, Italy.; ^4^ The Department of Biochemistry and Molecular Biology, University of British Columbia, Vancouver V6T 1Z3, Canada.

## Abstract

The KCNQ1 (Kv7.1) potassium ion (K^+^) channel plays a critical role in cardiac repolarization, while loss- or gain-of-function mutations increase the risk of life-threatening arrythmias and sudden death. Like many channels, KCNQ1 exhibits state-dependent pharmacological properties with inhibitors such as UCL2077 displaying higher potency for intermediate over activated states. Here, we report cryo-EM structures and MD simulations of KCNQ1 in complex with UCL2077 in two activation states. UCL2077 breaks tetrameric symmetry and binds in diagonally opposed protomers in pockets formed by the S5-S6 helices from one protomer and the S6 of an adjacent protomer. UCL2077 binding drives distortion of the S6 helix and causes an allosteric collapse of the pore. Specificity arises via structural configurations in the pore that occur with greater frequency in the intermediate state and are stabilized upon drug binding.

## INTRODUCTION

KCNQ1 (Kv7.1) is a voltage-gated potassium channel protein with critical roles in membrane ion transport throughout the human body (*1*), contributing to physiological processes such as cardiac repolarization (*2*, *3*), epithelial ion transport (*2*, *4*, *5*), and auditory function (*6*). In the heart, KCNQ1 assembles with KCNE1 to form the slow delayed rectifier current, known as *I*_Ks_ (*2*, *3*). The dysregulation of this major repolarizing current, caused by mutations in KCNQ1, is associated with atrial fibrillation, the most common nonlethal cardiac arrhythmia (*7*), and less common life-threatening arrythmias that include long QT syndrome type 1 (LQT1) and short QT syndrome type 2 (SQT2).

KCNQ1 is a homotetrameric channel, with each protomer composed of six transmembrane helices (TMHs; S1 to S6), as well as intracellular helices HA-HD, of which HA-HB interact with CaM in a 1:1 stoichiometry (Fig. 1A) (*8*–*10*). The TMHs form the voltage sensor domain (VSD; helices S1 to S4) as well as the pore domain [PD; helices S5 to S6, selectivity filter, and selectivity filter helix (FH)]. The VSD is linked to the PD through the S4-S5 linker (S4-S5L) and interacts with the PD of an adjacent subunit (PD′), resulting in a domain-swapped configuration (Fig. 1A). Building on functional and initial structural descriptions of KCNQ1, recent cryo–electron microscopy (cryo-EM) studies have transformed our understanding of its physiological regulation by revealing the discrete conformational steps of channel activation and the VSD-PD coupling mechanisms vital for ion conduction (*9*–*15*). While these advances have captured activator-bound KCNQ1 structures, inhibitor-bound states remain elusive. Understanding how inhibitors block KCNQ1 is essential to develop new treatments for gain-of-function disorders such as atrial fibrillation and SQT2.

**Fig. 1.**
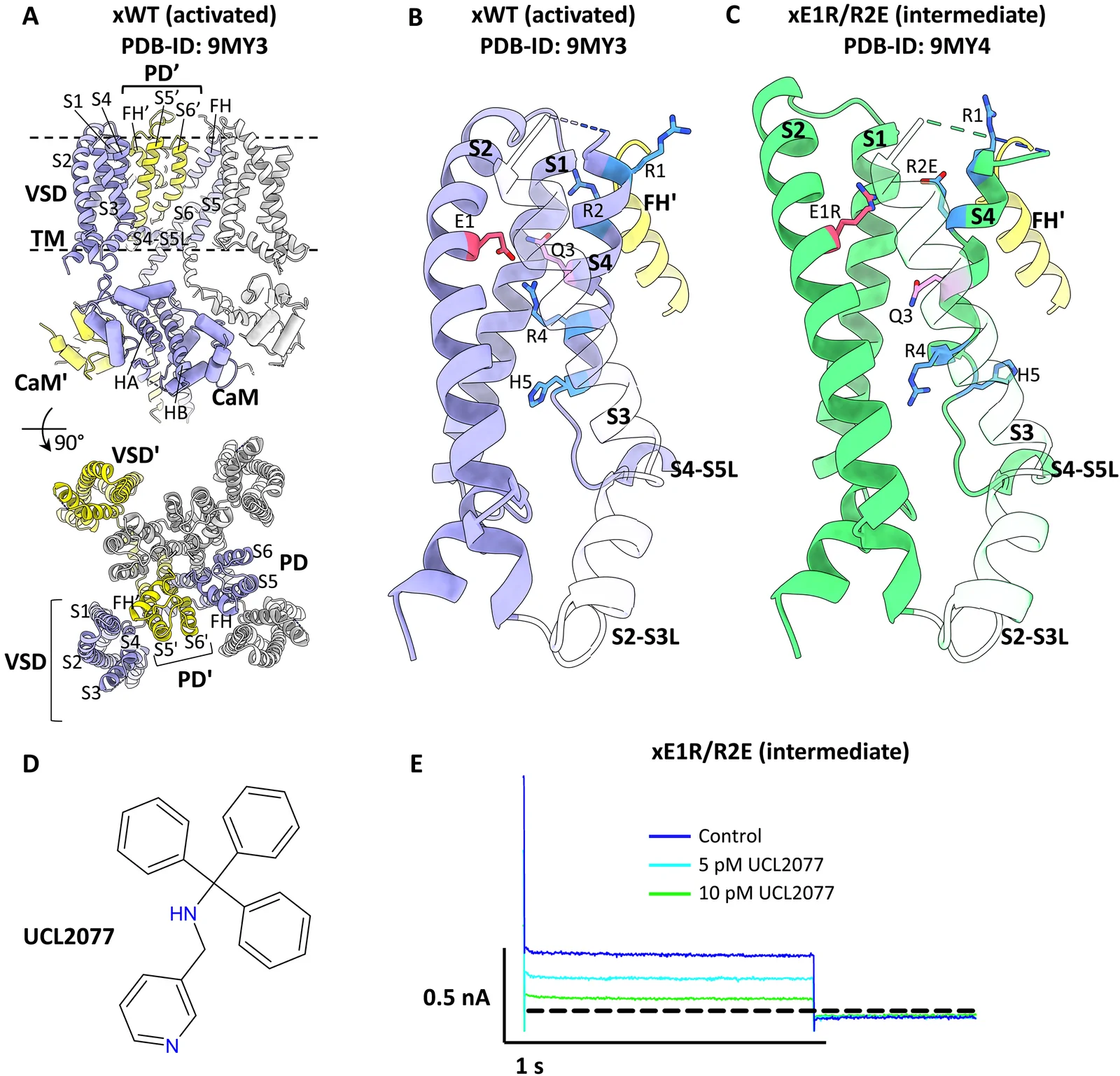
Structures of the xWT activated-state (PDB ID: 9MY3) and xE1R/R2E intermediate-state (PDB ID: 9MY4) KCNQ1 channels. (**A**) Side and top views of the xWT activated-state structure. One pair of domain-swapped protomers is colored purple and yellow, and the second pair is shown in white for clarity. CaM is displayed as tube helices. (**B** and **C**) VSD configurations of the xWT-activated (B, purple) and xE1R/R2E-intermediate (C, green) structures (*20*). Positively charged residues are shown in blue [R1 (xR218), R2 (xR221), R4 (xR227), H5 (xH230)], negatively charged residues in red [E1 (xE150)], and Q3 (xQ224) in pink. The adjacent FH helix (FH′) is highlighted in yellow to emphasize its spatial relationship with S1 in each state. (**D**) 2D chemical structure of UCL2077. (**E**) Whole-cell patch recordings of xE1R/R2E exposed to UCL2077. Construct as used for cryo-EM, but uncleaved from GFP, expressed in tsA201 cells. Pulses were every 15 s from a holding potential of −90 to 60 mV for 4 s and then to −40 mV for 0.9 s to record tail currents. Only tail current and holding current are shown, before (blue) and after 5 pM (cyan) or 10 pM (green) UCL2077. The dashed line indicates 0 pA.

KCNQ1 inhibition is challenging to study because the channel displays strong, yet not mechanistically understood, state-dependent physiology and pharmacology as the S4 helix transitions between the “down” resting state and the “up” activated state. The S4 movement is mediated by arginine residues, R1, R2, and R4 [R218, R221, and R227 in *Xenopus* KCNQ1 (xKCNQ1); Fig. 1B], and engineered charge-reversal pairs between these residues and E1 (E150 in xKCNQ1) in the S2 can stabilize the channel into specific functional states along the activation continuum (*16*–*18*); E1R/R1E represents a resting down state, E1R/R2E an intermediate state, and E1R/R4E the activated up state. While both the intermediate and activated constructs are constitutively active in vitro, the intermediate state is more sensitive to inhibitors (*17*, *19*), highlighting its potential therapeutic relevance. In detergent conditions, where no voltage is applied, the wild-type (WT) VSD S4 helix adopts the activated up conformation, with E1 forming a salt bridge with R4 (Fig. 1B). In this state, S1 is curved and contacts the FH of the neighboring protomer (FH′; Fig. 1B). By contrast, in the E1R/R2E intermediate structure, S4 remains largely up, but S1 is straight and detached from the neighboring FH (Fig. 1C) (*20*). These structural differences provide a basis for exploring the mechanisms of state-dependent inhibition of KCNQ1.

Among the KCNQ1 inhibitors, UCL2077 (Fig. 1D) is most interesting as it is a potent, state-dependent and highly isoform-selective KCNQ1 inhibitor (*21*) with IC_50_ (median inhibitory concentration) values in the picomolar range for the E1R/R2E intermediate state and the nanomolar range for the activated state (*19*). UCL2077 [3-(triphenyl methyl-aminomethyl)pyridine] was developed to inhibit Ca^2+^-activated K^+^ channels in hippocampal neurons (*22*). However, Soh *et al.* (*21*) tested UCL2077 on mammalian cells expressing KCNQ channels and found that it selectively inhibits KCNQ1 channels over other KCNQ subtypes. UCL2077 was originally thought to enter the open pore and prevent the channel reaching the activated state (*21*), an idea supported by voltage clamp fluorescence studies (*23*). However, the mechanism of this action remains unknown. Here, we report the cryo-EM structures of the xKCNQ1 E1R/R2E (xE1R/R2E) intermediate and WT (xWT)–activated states in the presence of UCL2077. The structures reveal that, in contrast to previously studied compounds that bind to KCNQ channels, UCL2077 breaks the fourfold symmetry of the channel and binds only in two diagonally opposed protomers. The binding site is in a hydrophobic pocket formed by the S5-S6 helices and FH from one protomer, and the S6 helix from the adjacent protomer. The binding of UCL2077 in the xE1R/R2E structure triggers VSD rotation as well as global and local S6 conformational changes that force S6 residues toward the pore axis and collapse of the ion permeation pathway. Our structural and molecular dynamics (MD) studies reveal the physical and kinetic bases for the preference of UCL2077 for the xE1R/R2E intermediate state over the xWT-activated state and its selectivity for KCNQ1 over other KCNQs. Hence, our work begins to shed insight into poorly understood mechanisms of state-dependent channel inhibition.

## RESULTS

### Binding of UCL2077 in the xE1R/R2E intermediate state breaks fourfold symmetry

To evaluate the binding of UCL2077 in the xE1R/R2E intermediate state structure, a cryo-EM dataset was collected in the presence of 250 μM UCL2077 using the same construct we recently described a native structure for (*20*). Electrophysiological recordings verified that this *Xenopus* construct is sensitive to UCL2077 and exhibits 57 ± 7% inhibition at 10 pM [means ± SEM (*n* = 3); Fig. 1E], similar to that reported for the human full-length construct (*19*).

As the binding site was unknown, data analysis and initial reconstructions were performed without imposing symmetry (see Materials and Methods and fig. S1, A to I). The resulting C1 reconstruction revealed a binding mode consistent with twofold rotational symmetry (fig. S1, H and I), and C2 symmetry was subsequently imposed, yielding a reconstruction at 3.95-Å resolution (fig. S1, J to N and sample maps in fig. S2). To identify the binding site, we compared the cryo-EM map of xE1R/R2E-CaM-UCL2077 with our previous, C4-symmetric, native xE1R/R2E-CaM map (*20*). Side views of these reconstructions are shown in Fig. 2 (A and B) with the adjacent protomers colored red/orange for xE1R/R2E-CaM-UCL2077 and green/lime for native xE1R/R2E-CaM. Calmodulin (CaM) is colored white and unmodeled densities dark gray. Density corresponding to the ligand (yellow) was found between the S5 and S6 helices of protomer P1 but was not observed at the equivalent site in the immediately adjacent, 90°-rotated protomer P2 (Fig. 2A, insets), nor in the native channel (Fig. 2B, inset). As the channel complex exhibits C2 symmetry, UCL2077 density is again evident in protomer P3 and absent in P4. This alternating pattern is more clearly illustrated in the top view cross section in Fig. 2C, which reveals ligand density in protomers P1 and P3 but not in P2 and P4. By contrast, the C4-symmetric native channel shows no comparable density at the equivalent site(s) (Fig. 2D), nor do the C1 or C2 maps. We next analyzed local resolution across the reconstruction, which revealed higher resolution in the PD compared with the VSD (fig. S1K), consistent with previous observations for the native xE1R/R2E-CaM channel (*20*), with the ligand fitting comfortably within the map (Fig. 2E and fig. S1K).

**Fig. 2.**
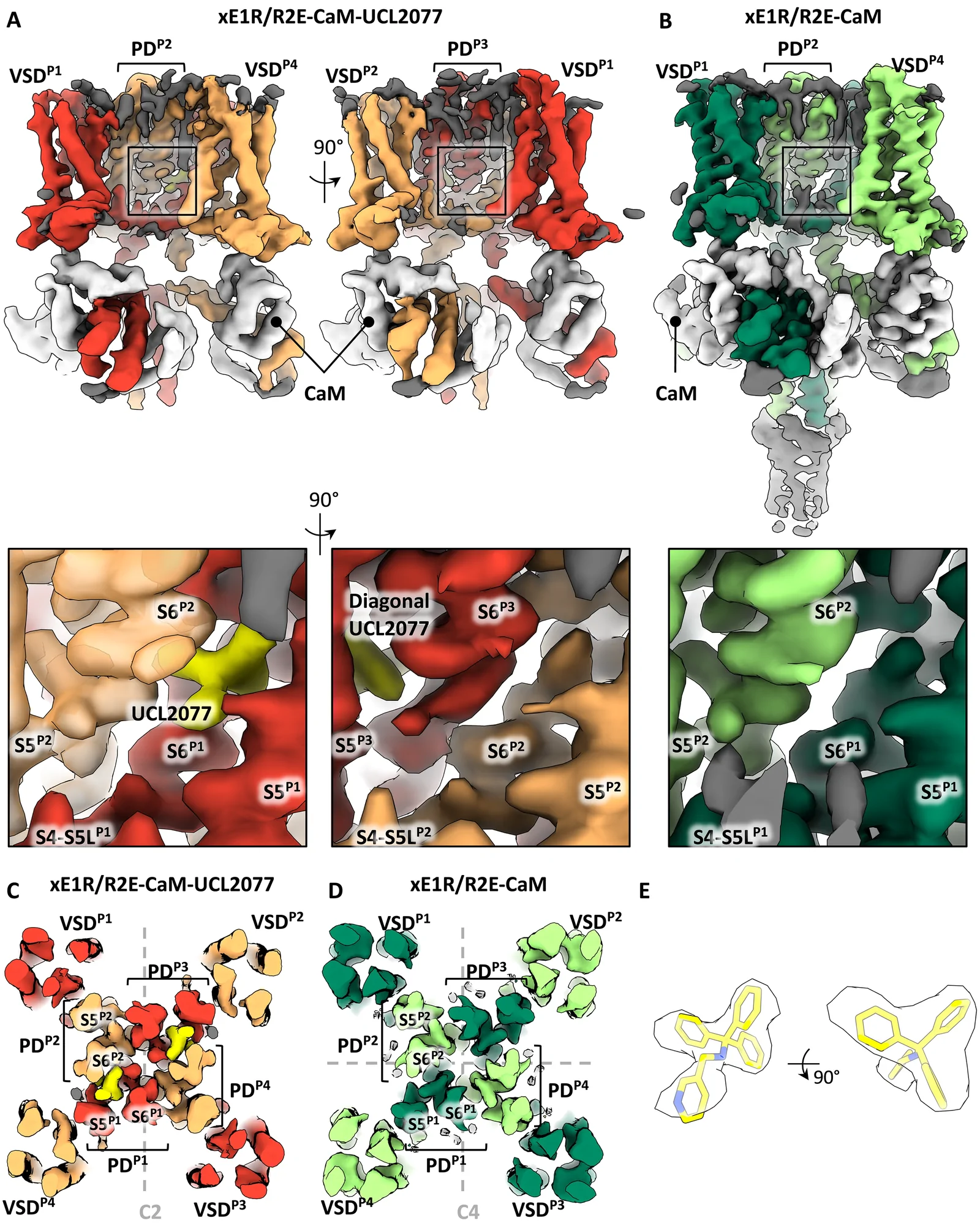
Cryo-EM maps of xE1R/R2E-CaM in the absence or presence of UCL2077. Diagonally opposed protomers of the xE1R/R2E channel in the presence or absence of UCL2077 are colored red/orange and green/lime, respectively. CaM densities are colored white, unmodelled densities dark gray, and UCL2077 densities yellow. (**A**) Side-view cryo-EM reconstruction of the xE1R/R2E-CaM-UCL2077 complex. 0° and 90° views are shown due to the C2 symmetry of the map. (**B**) Cryo-EM map of the native C4-symmetric xE1R/R2E-CaM channel for reference (*20*). Insets zoom in on the corresponding binding site region. (**C** and **D**) Top view cryo-EM map cross section at the level of UCL2077 for the ligand-bound (C) and native channels (*20*) (D). (**E**) UCL2077 fit in the cryo-EM map. The xE1R/R2E-UCL2077 cryo-map is shown at a contour level of 0.0275 and for native xE1R/R2E at 0.103.

A tetrameric structural model built in the C2-symmetric map rotated by 90° is illustrated in Fig. 3 (A and B) with CaM, colored white, attached to the VSD as it is in the native xE1R/R2E and xWT channels (*20*). UCL2077, colored yellow, binds in the transmembrane (TM) region formed primarily by the PD (S5, FH, and S6 helices) of one protomer (PD^P1^, Fig. 3A, red color) with secondary contributions from the S6 helix of the adjacent protomer (S6^P2^, Fig. 3A, orange color). The binding site is lined by residues; xT255^P1^, xL256^P1^ in the S5^P1^; xF325^P1^, xF329^P1^, xF330^P1^, and xP333^P1^ in the S6^P1^; xV300^P1^ and xT301^P1^ in the FH^P1^; and residues xV324^P2^, xI327^P2^, and xS328^P2^ in the adjacent S6^P2^ (Fig. 3, A and B, insets; cryo-EM map for binding site residues is shown in fig. S3). Since UCL2077 exhibits a C2-symmetry binding mode, only two diagonal PDs are occupied by the ligand (P1/P3), while their adjacent PDs are free of ligand (P2/P4). Consequently, the UCL2077-bound PD^P1^ (Fig. 3A) interacts with both UCL2077 free protomers; the adjacent S6 helix (PD^P2^; Fig. 3A) and the domain-swapped VSD^P4^ (Fig. 3B), demonstrating how ligand binding can simultaneously bridge multiple structural elements across the tetramer.

**Fig. 3.**
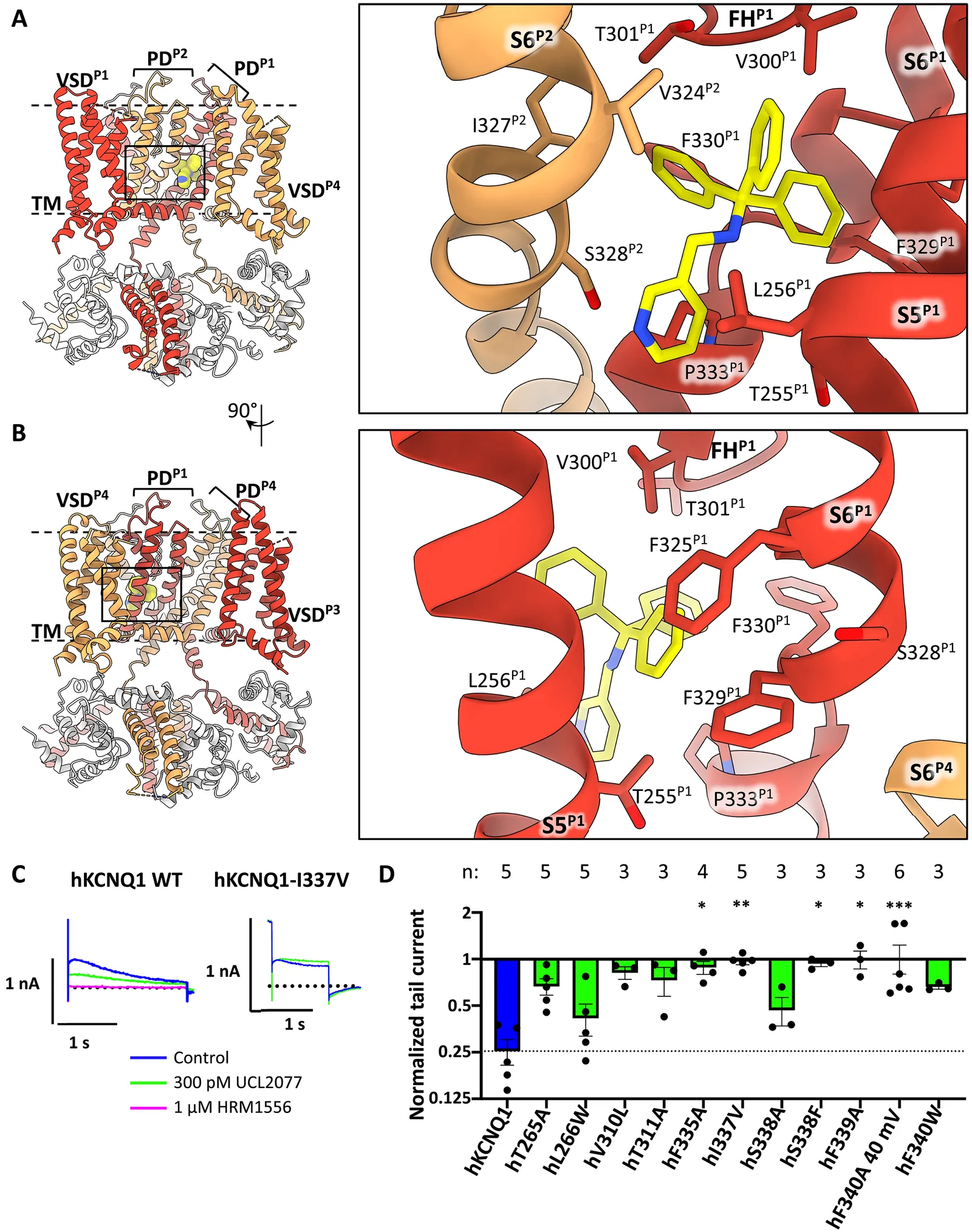
Structure of the xE1R/R2E-CaM-UCL2077 complex and interactions with binding site residues. Diagonally opposed protomers of xE1R/R2E are colored red/orange, CaM in white, and UCL2077 in yellow. (**A**) Side view of the tetrameric channel showing the UCL2077 bound at the interface formed by the S5-S6 helices of one protomer and the S6 helix of the adjacent protomer. The inset provides a close-up of the ligand binding pocket, highlighting residues that interact with UCL2077 and S328^P2^, which does not. (**B**) Side view of the complex channel structure rotated 90° relative to (A) to reveal additional interacting residues not visible in the initial orientation. (**C**) Representative tail current traces of WT hKCNQ1 and the hI337V mutant in control (blue) 0.3 nM UCL2077 (green) or 1 μM HMR1556 (magenta). Cells were pulsed from a holding potential of −90 to 60 mV (except hF340, in (D), which was pulsed to +40 mV) for 4 s and then for 0.9 or 2 s at −40 mV. Dotted lines denote zero current. (**D**) Initial tail current amplitudes in 300 pM UCL2077 were normalized to control values before drug exposure. Bars represent means ± SEM. Only values that were statistically different from WT using one-way Anova are starred. *P < 0.05, **P < 0.01, and ****P < 0.001. Data and figure were analyzed and prepared using Clampfit 10 (Molecular Devices) and GraphPad Prism 10 (GraphPad Software, Boston, MA, USA).

### Mutational studies and MD simulations validate the binding site in the intermediate state

To confirm the binding site, residues in *human* KCNQ1 (hKCNQ1) shown to interact with UCL2077 in the *Xenopus* cryo-EM structure were mutated, except in particular hP343 where mutations are not tolerated (Fig. 3, C and D) (*24*, *25*). Note that residue numbering for hKCNQ1 is +10 with respect to xKCNQ1. Since the other KCNQs were shown to be largely insensitive to UCL2077 (*21*), when appropriate we changed the residue in hKCNQ1 to that found in the other KCNQs, where they differed (Fig. 3D). That was the case for hL266W, hV310L, hT311A (only KCNQ3), and hI337V (only KCNQ2 and KCNQ3). Cells expressing the hWT and mutant forms of hKCNQ1 were pulsed repetitively to +60 mV for 4 s under voltage clamp and exposed to 300 pM UCL2077 (Materials and Methods). Inhibition was evaluated from initial tail current amplitude at −40 mV in the presence of UCL2077 compared with control (Fig. 3D).

Mutation at hV310, hF335, and hF339, markedly diminished the effect of the inhibitor, highlighting their central role in ligand binding (Fig. 3D). In addition, mutations at hF340, not only a residue interacting with UCL2077 but also important for the UCL2077 mechanism of action, (elaborated on below), significantly reduced the effectiveness of UCL2077 when mutated to alanine. hF340W showed reduced sensitivity to UCL2077 (Fig. 3D) and indicates that side-chain size at this location is important for channel inhibition. The mutation of hI337V, a subtle change in side-chain size, also significantly reduced blocker efficacy. Although hL266 sits at the mouth of the binding pocket, the bulky hL266W mutation did not significantly alter blocker effectiveness. A similarly modest effect was observed for hT311A, which suggests that although L266 and T311 help form the binding site, they are not critical determinants of inhibitor binding or selectivity. In addition, while hS338 was not identified as a residue in contact with UCL2077 (S328^P2^; Fig. 3A), mutation to a larger residue (hS338F) might indicate the pathway UCL2077 uses to gain access to the binding site. The bulkier phenylalanine does significantly reduce the effectiveness of the blocker, whereas loss of a polar residue here (hS338A) does not seem to affect potency.

The cryo-EM map unambiguously defines the ligand-binding pocket and the key interacting residues. However, the near symmetry of the ligand allows multiple orientations that are compatible with the density. To determine the most likely pose, as explained in detail in Materials and Methods, we leveraged computational approaches, including Rosetta EMERALD (*26*), AutoDock (*27*), and MD simulations. Across all approaches (figs. S4 to S7), the data consistently suggested a single preferred binding orientation, which preserved the interactions described above. The pose shown in Fig. 3 represents this cross-validated, most stable, and reproducible configuration, providing a robust representation of the ligand-bound state. To further evaluate its stability, MD simulations of the top-ranked pose were performed using only the VSDs and PDs of the xE1R/R2E structural model determined here, with the intracellular helices and CaM removed (Fig. 4A). Three independent replicas were run for 500 ns each, following 500 ns of equilibration with minimal restraints applied to the gate to allow proper pore hydration. Over the whole runs, the protein backbone root mean square deviation (RMSD) plateaued at ∼4.0 Å (Fig. 4A), indicating that the overall channel structure remained stable. The ligand retained well-defined binding poses, exhibiting only minor positional fluctuations, primarily involving the pyridine ring, which is more exposed to the solvent and thus more flexible (Fig. 4B). Residues lining the binding pocket showed minimal deviations from their initial coordinates, further supporting the stability of the intermolecular interactions and the reliability of the complex under dynamic conditions.

**Fig. 4.**
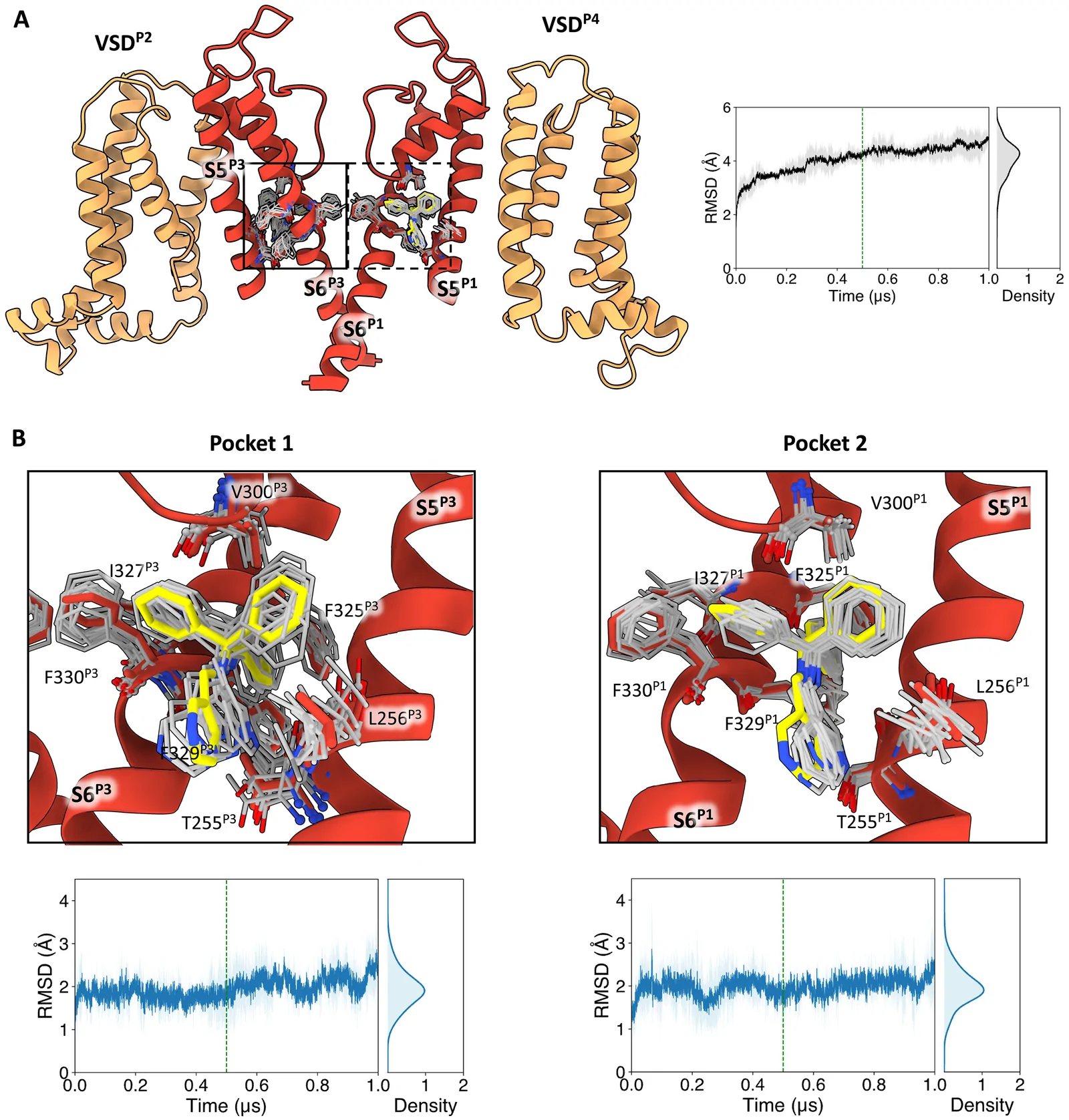
MD simulations of the xE1R/R2E-UCL2077 complex structure confirm stable ligand binding across replicas. (**A**) Portion of the channel used for the 1 μs MD simulations. Only the PDs that bind UCL2077 (red) and their domain-swapped VSDs (orange) are shown for clarity. Graph shows RMSD over time, with kernel density estimates (KDEs) of aggregated replicas on the right. (**B**) Ligand binding dynamics in the two binding pockets over the course of the simulations. The initial positions of the binding site residues and the ligand are shown as red and yellow cylinders, respectively, with conformational changes during the simulation represented as gray sticks and captured every 50 ns. Graphs below show ligand RMSD in each pocket over time, with KDE of aggregated replicas on the right.

### The binding of UCL2077 in the intermediate state induces PD conformational collapse

In comparison to the native xE1R/R2E structure, we found that the binding of UCL2077 causes conformational changes in the S6 helices. In the native xE1R/R2E structure (*20*), the UCL2077 binding site is partially occluded by the S6 helix which displays a kinked configuration at the PAG (xP333) motif (Fig. 5A, white). This kink is preserved for the S6 helices in the UCL2077-free P2 protomer (Fig. 5A, orange) but changes to helix-loop-helix in the UCL2077-bound P1 protomer, which places residues above the PAG motif closer to the pore axis (Fig. 5A, red). More specifically, the S6 residues near the PAG motif in the UCL2077-free protomers maintain their native conformations (Fig. 5B, white/orange), while those in the UCL2077-bound protomers undergo notable local conformational changes. These changes include a displacement of the side chain of xF330^P1^ into the inner vestibule to accommodate ligand binding, a movement of xA331^P1^ toward the pore axis and a one-residue register shift of xI327^P1^ along the helix (Fig. 5B, red/white, and movie S1). These local adjustments propagate along the helix, resulting in larger scale S6 shifts. Focusing on the gate residues in the lower S6 (xG335, xS339, and xL343; Fig. 5A), the distances between xL343-Cα residues deviate from the native xE1R/R2E structure by between ∼4.0 and 2.0 Å for the UCL2077-bound S6^P1/P3^ and UCL2077-free S6^P2/P4^ helices, respectively (Fig. 5C). This S6 gate conformation (∼16 Å^P1/P3^, ∼14 Å^P2/P4^ measured at xL343-Cα) is somewhere in between the closed [∼12 Å (*20*); Fig. 5C, white] and open [∼20 Å (*10*, *28*, *29*)] channels.

**Fig. 5.**
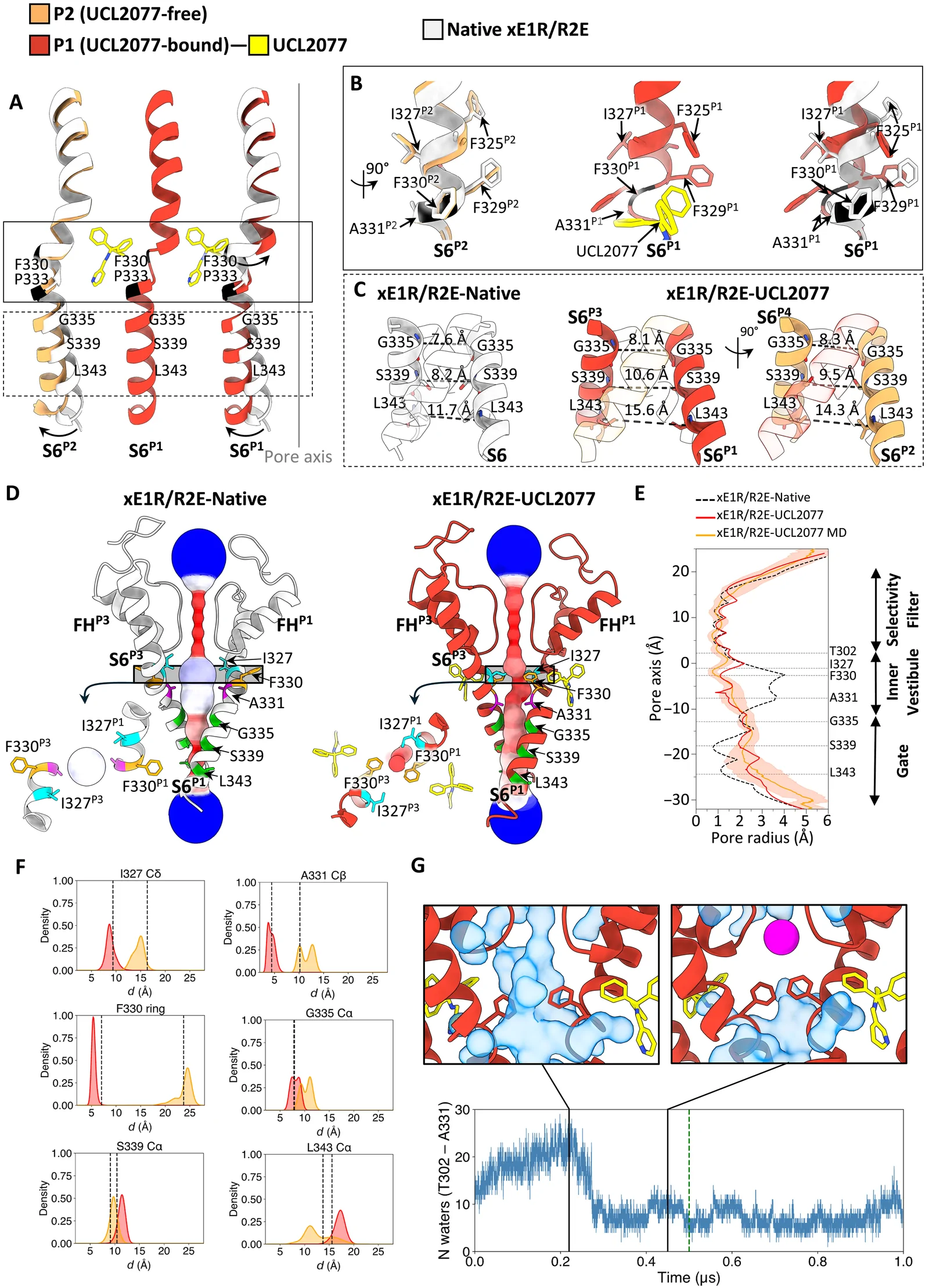
Conformational changes in the S6 helix upon UCL2077 binding in the xE1R/R2E structure. Native xE1R/R2E (*20*) is shown for reference in white, UCL2077-bound xE1R/R2E protomers in red (P1 and P3), UCL2077-free protomers in orange (P2 and P4), and UCL2077 in yellow. (**A**) The S6 helix adopts a helix-loop-helix configuration at the PAG motif in the UCL2077-bound protomers. xP333 (PAG motif) and xF330 are colored black. (**B**) Close-up view of the UCL2077 binding region, rotated 90° relative to (A), highlighting local S6 conformational changes associated with UCL2077 binding. (**C**) Side views showing distances between gate residues in the native xE1R/R2E and ligand-bound structure. (**D**) Pore-axis radius profiles for the native xE1R/R2E and UCL2077-bound structure with narrower regions shown in red and wider regions in blue. Only two diagonally opposed protomers are shown for clarity; inset shows a top-view representation emphasizing the restriction at the level of xF330. Gate residues (xG335, xS339, and xL343), xI327, and xF330 are shown as green, cyan, and orange cylinders, respectively. (**E**) HOLE pore radius profiles of the native (dashed black line) and drug-bound (in red) structures and the average pore profile from MD simulations (orange, shadow area represents SD). (**F**) KDEs of distances between facing residues in the inner vestibule (xΙ327Cδ, xF330 ring, and xA331Cβ) and gate region (xG335Cα, xS339Cα, and xL343Cα), computed from replica-aggregated data. Black vertical lines indicate reference values from the cryo-EM structure. (**G**) Pore hydration at the level of xF330 during MD simulations of the xE1R/R2E-UCL2077 structure. The plot shows the number of water molecules over time between residues xT302 and xA331. Representative snapshots are taken from the simulation at 220 and 450 ns. Water molecules are represented as a cyan surface and a potassium ion as a magenta sphere.

The observed gate dilation is typically associated with channel activators rather than inhibitors. However, the conformational changes in the S6 helices, place the diagonally opposed xF330 residues to face the pore axis, which constricts the permeation pathway. The analysis of pore profiles in the native (*20*) and UCL2077-bound structures revealed that UCL2077 binding reduces the radius of key constriction sites at the level of the xF330^P1/P3^ and xA331^P1/P3^ (Fig. 5, D and E). The narrowing was maintained across all three MD simulation replicas (Fig. 5E), with stable distances between opposing residues in the inner vestibule and gate (Fig. 5F); notably, the distance between the facing rings of inward-pointing xF330 residues (5.66 Å) was even smaller than in the cryo-EM structure (6.88 Å). Consequently, the diagonal binding of UCL2077 changes the hydration properties of the inner vestibule, as revealed by MD simulations showing progressive dehydration at the level of xF330 (Fig. 5G, movie S2, and fig. S8). Hence, ligand binding induces conformational changes that propagate through the channel, ultimately stabilizing a constricted, locally dehydrated pore conformation.

### Intermediate state VSD rotation caused by UCL2077 binding

The UCL2077 binding in the PD not only produces S6 rearrangements, but it also triggers shifts in the VSDs and the PD-VSD coupling mechanical bridge, the S4-S5L. Top and side views in Fig. 6 show PD-based superposition of xE1R/R2E-UCL2077 (red/orange) onto the native xE1R/R2E structure (white). The alignment is focused on the domain-swapped PD-VSD pairs formed by the PD^P2^ from protomer 2 (PD free of UCL2077) and the VSD^P1^ from protomer 1 (PD binds UCL2077, Fig. 6, A and C), as well as the PD^P1^ from protomer 1 and VSD^P4^ from protomer 4 (PD free of UCL2077, Fig. 6, B and D). Although the densities for the upper half of the VSDs in the xE1R/R2E-CaM-UCL2077 map were too weak to build side chains (fig. S9, A and B), comparison between the PD^P2^-VSD^P1^ pair shows that the VSD^P1^ largely preserves its native S1-FH detached conformation with respect to the PD (Fig. 6, A and C). In contrast, for the PD^P1^-VSD^P4^ pair, the VSD S1-S2 helices move closer to the UCL2077-bound FH^P1^, adopting an S1^P4^-FH^P1^ attached-like conformation (Fig. 6, B and D). In both cases, the VSD helices are rotated clockwise with the VSD^P4^ being rotated to a greater degree (movie S3), which brings the S1^P4^ helix ∼7 Å (measured between xL132-xW313 Cα) closer to the diagonally opposed S6^P2^ (Fig. 6B, top view). MD simulations of the UCL2077-bound xE1R/R2E structure show that the PD-VSD attached and detached conformations behave differently. While the attached one is stable, with interdomain distances (measured between xA290 and xI135) even shorter than in the cryo-EM structure for VSD^P4^ and PD^P1^ (Fig. 6D, inset graphs), the detached one samples a broader range of distances and is less well maintained (Fig. 6C, inset graphs). In addition, the S4-S5L is displaced downward by ∼1.4 and ∼2.0 Å in the P2 and P1 protomers, respectively (Fig. 6, C and D, measured at xW238-Cα) potentially contributing to the shifts in the VSD. The positioning of the S1^P4^ helix near the S6^P2^ (Fig. 6B) is also evident in comparison with the native xWT channel, in which the S1 helix is attached to the adjacent FH (fig. S9, C and D). This diagonal VSD^P4^-PD^P2^ proximity has not been observed before for KCNQ1 and may signal the importance of flexibility of the S1-S2 helices in the intermediate state in modulating drug sensitivity.

**Fig. 6.**
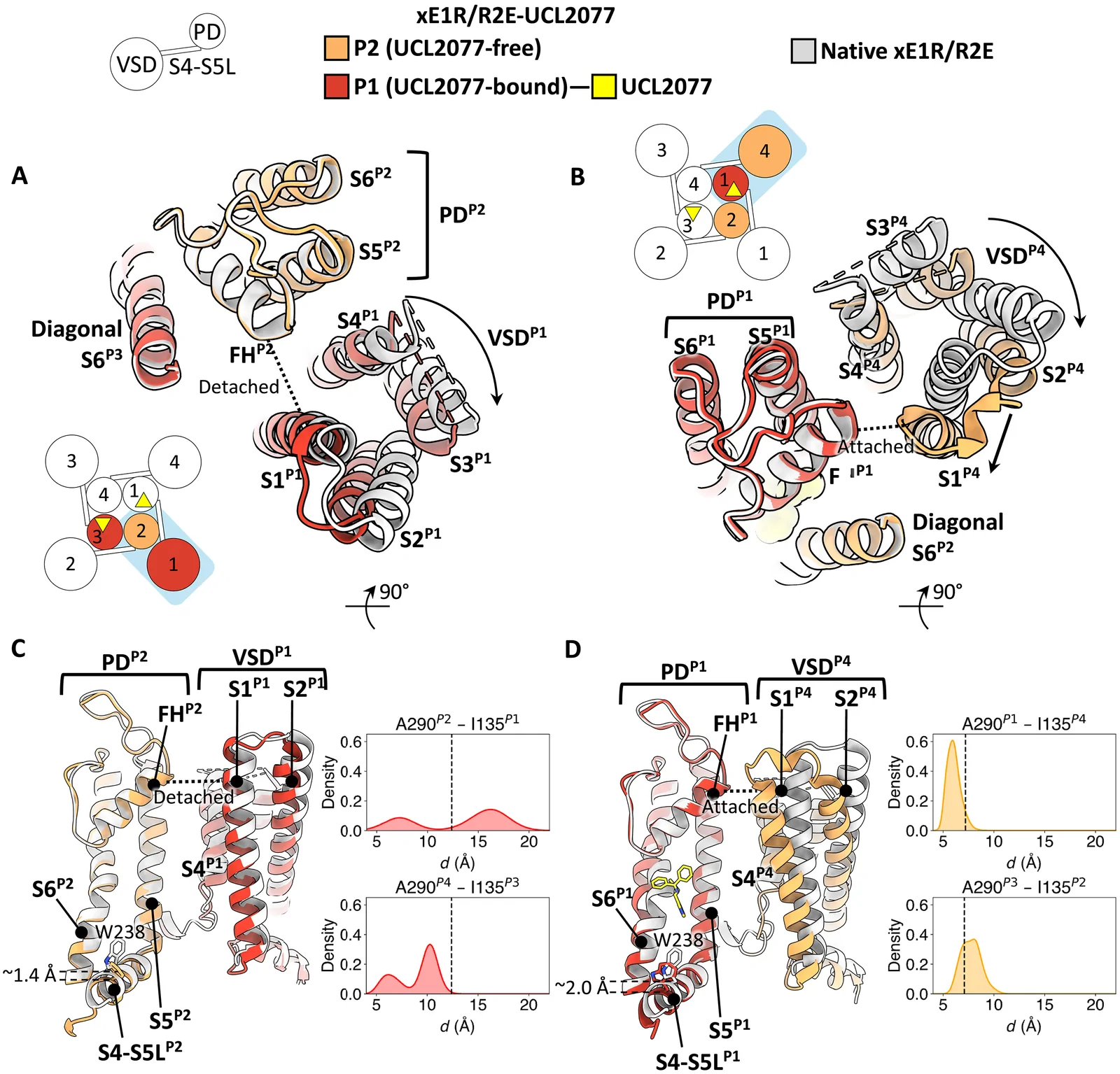
UCL2077 induces shifts in the VSD in the xE1R/R2E structure. Comparison of domain-swapped PD-VSD pairs in ligand-bound and ligand-free xE1R/R2E protomers. The xE1R/R2E native structure (*20*) is shown for reference in white, UCL2077 PD-bound protomers in red (P1 and P3), PD-free protomers in orange (P2 and P4), and UCL2077 in yellow. Top (**A** and **B**) and side (**C** and **D**) views are shown. (A and C) Comparison focused on the PD-bound VSD (P1, red) and the adjacent UCL2077-free PD (P2, orange). (B and D) Comparison focused on the UCL2077-bound PD (P1, red) and the adjacent PD-free VSD (P4, orange). In (C) and (D), the S6 helix diagonally opposed to the VSD has been removed for clarity. The plots show KDEs of distances between PDs (A290Cα) and VSDs (I135Cα) for the four protomers from aggregated MD replica data. Vertical lines indicate reference values in the cryo-EM structures.

### Structural and kinetic determinants of intermediate-state selectivity

WT cryo-EM structures of KCNQ1 that represent the fully activated state of the channel have been solved and show the VSD adopting a common activated conformation (*9*–*11*, *20*, *29*). Accordingly, to investigate differential state-dependent binding in KCNQ1, the xWT KCNQ1 construct (*20*) was purified in detergent (0 mV), followed by the addition of 500 μM UCL2077, and a cryo-EM dataset was collected (figs. S10 and S11 and table S1). Compared with the xE1R/R2E-UCL2077, the xWT-UCL2077 dataset was more heterogeneous (see Materials and Methods), and a high-resolution map at 3.81 Å was obtained only with C2-symmetry imposed. Still, the cryo-EM map (Fig. 7A) and the structural model (Fig. 7B) show UCL2077 (colored yellow) bound at the same location and causing a displacement of xF330 in the inner vestibule, as in the xE1R/R2E-UCL2077 structure (Fig. 2C), but the density for its 3-pyridyl group was absent (Fig. 7C; cryo-EM map for binding site residues is shown in fig. S12). This, along with the not well-resolved CaM (fig. S10B), reflects the increased sample heterogeneity. A more systematic analysis of this heterogeneity in comparison to the xE1R/R2E-UCL2077 can be found in the Supplementary Materials.

**Fig. 7.**
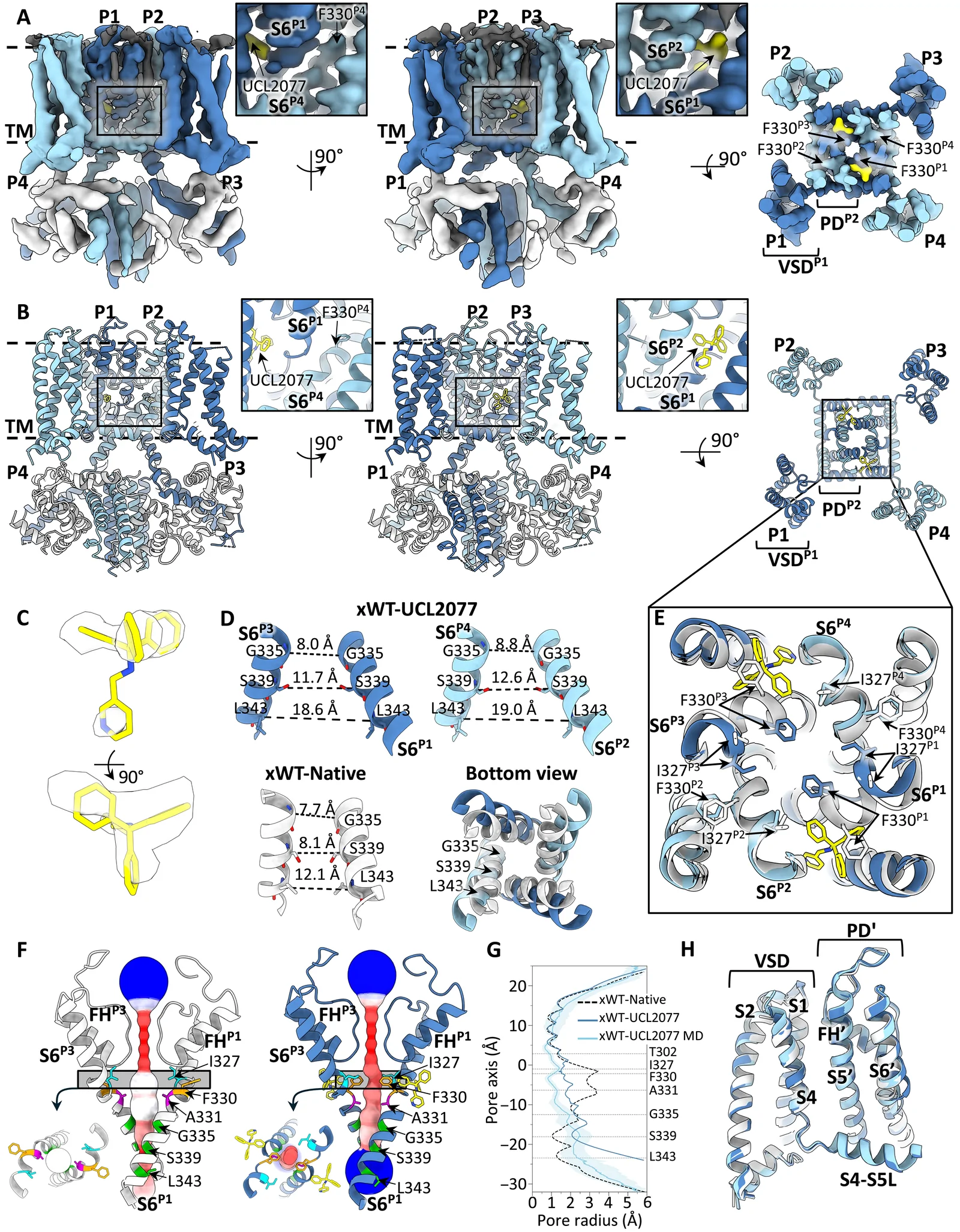
The binding of UCL2077 in the xWT channel. Two side views rotated by 90° and a top view of the cryo-EM map (**A**) and structural model (**B**) of xWT in the presence of UCL2077. Insets show zoom-in of UCL2077 binding site. Diagonally opposed protomers that bind UCL2077 (P1 and P3) are colored blue and the protomers that do not bind UCL2077 (P2 and P4) are colored cyan. UCL2077 is colored yellow, CaM light gray, and unmodelled densities dark gray. (**C**) UCL2077 fit in the cryo-EM map. (**D**) Distance between α carbons of S6 gate residues in the native (*20*) (white; PDB-ID: 9MY3) and UCL2077-bound (blue/cyan) protomers. (**E**) Top view representation of xWT-UCL2077 (shades of blue) and xWT native (*20*) (white) clipped at the level of UCL2077 binding site. (**F**) Pore radius profiles for the native xWT and UCL2077-bound structures with narrower regions shown in red and wider regions in blue. Only two diagonally opposed protomers are shown for clarity; insets show top-view representations emphasizing the restriction at the level of xI327 and xF330. Gate residues (xG335, xS339, and xL343), xI327, and xF330 are shown as green, cyan, and orange cylinders, respectively. (**G**) Pore radius profiles of the native (*20*) (dashed black line) and drug-bound xWT structure (solid blue line) and average pore profile from MD simulations (light blue, shadow area represents S.D.) (*20*). (**H**) Superposition of PD-VSD UCL2077-bound and UCL2077-free pairs with native xWT (white) (*20*). The xWT-UCL2077 cryo-map is shown at a contour level of 0.0951.

The binding of UCL2077 in the xWT structure induces global and local S6 conformational changes, as observed for the xE1R/R2E-CaM-UCL2077 structure, with two xF330 residues collapsing the ion permeation pathway (Fig. 7, E to G). However, the gate was completely open, displaying an almost C4-symmetric configuration (∼19.0 Å measured at xL343-Cα; Fig. 7D). In this open state, the resolvable CaM is not rotated as in the fully open PIP2-bound structures (*10*, *28*, *29*) and still interacts with the VSD (Fig. 7B). The VSD helices and the S4-S5L maintain their activated state conformation, as shown in Fig. 7H where the domain-swapped UCL2077-bound (blue) and UCL2077-free (cyan) pairs are aligned onto the native xWT-activated structure (white) (*20*). Consequently, despite the similarity in the binding mode between the xE1R/R2E and xWT-UCL2077–bound states, the presence of UCL2077 in the activated state leaves the gate completely open but does not lead to VSD rotation, and the S1 helix remains attached to the adjacent FH (FH′). Therefore, the xWT-UCL2077 bound channel adopts an activated open configuration with CaM interacting with the VSD.

The xWT-UCL2077–bound state was also investigated with MD simulations. The protein backbone RMSD plateaued slightly below 4 Å (fig. S13A), and the ligand remained stable in both occupied pockets throughout all three runs (fig. S13B), confirming the binding mode observed in the cryo-EM map. Consistent with the xE1R/R2E-UCL2077 simulations, the inward displacement of xF330, xI327, and xA331 was maintained in all replicas. The distance between diagonally opposed xF330 residues reached values even smaller than those observed in the cryo-EM structure (fig. S13C). Notably, the average pore profile (Fig. 7G) and selected distance measurements (fig. S13C) reveal narrowing of both the inner vestibule and the gate relative to the cryo-EM map, indicating a shift toward an intermediate conformation, similar to that observed in the xE1R/R2E-UCL2077 structure (Fig. 5E), and dehydration at the level of xF330 (fig. S14). Last, the VSDs remained in a PD-attached conformation throughout the simulations (fig. S13D), consistent with the activated-state VSD arrangement observed in the cryo-EM structure (Fig. 7H), and at variance with the rotated VSD conformation seen in the xE1R/R2E-UCL2077 structure.

In both xE1R/R2E-UCL2077 and xWT-UCL2077 structures, the binding of UCL2077 is associated with a flip of two diagonally opposed xF330 residues toward the pore axis. To first understand why UCL2077 binds only in diagonally opposed protomers of KCNQ1, we used the UCL2077-bound xE1R/R2E protomers to generate a 4:4 UCL2077-bound tetramer and searched for potential clashes in the 4:0 [native structure (*20*), Fig. 8A; four xF330 out], 4:2 (UCL2077-bound structure, Fig. 8B; two xF330 in), and 4:4 (model generated UCL2077-bound tetramer, Fig. 8C; four xF330 in) KCNQ1:UCL2077 protomers. No clashes were observed in the native and UCL2077 bound structures (Fig. 8, A and B), but four phenylalanine side chains in the inner vestibule would clash with each other and with the adjacent xI327, which would also clash with UCL2077 (Fig. 8C). Therefore, the binding of UCL2077 molecules in all four or in two adjacent protomers is sterically hindered.

**Fig. 8.**
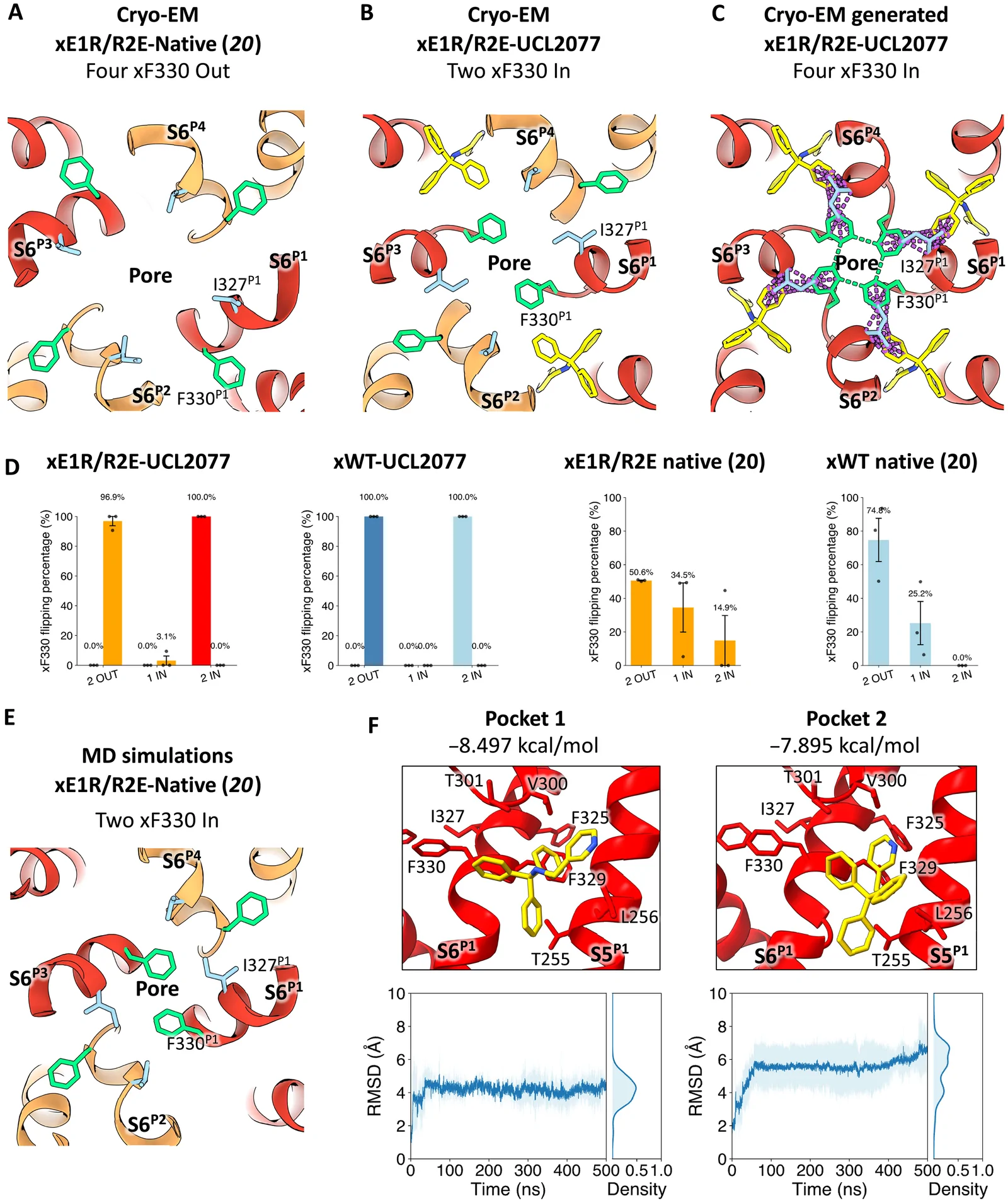
The C2-symmetric binding of UCL2077 is favored in the intermediate state. (**A** to **C**) Top-view representation, clipped at the level of xF330, with diagonally opposed protomers colored red and orange. xI327 and xF330 are shown as cyan and green cylinders, respectively. (A) Structure of native xE1R/R2E (*20*) with four xF330 facing outside the inner vestibule. (B) xE1R/R2E-UCL2077 structure with two xF330 residues in the inner vestibule. (C) Tetramer generated by two copies of the UCL2077-bound protomers with four xF330 residues in the inner vestibule. Clashes are shown as purple and green dashed lines. (**D**) Side-chain orientation of xF330 in (left to right): xE1R/R2E bound, xWT-bound, xE1R/R2E-intermediate, and xWT-activated simulated systems, averaged over three replicates with SEM. Data from bound structures are distinguished by chains pairs (red and blue bars: pair P1/P3 in xE1R/R2E and xWT, respectively; orange and cyan bars: pair P2/P4 in xE1R/R2E and xWT, respectively). (**E**) MD-derived conformation of native xE1R/R2E-intermediate state with two diagonally opposed xF330 residues in the inner vestibule (flipped-in). The diagonally opposed protomer pairs are colored red and orange. (**F**) Docking of UCL2077 onto the flipped-in native xE1R/R2E conformation extracted from the MD simulations; top: best poses with ligand and flexible residues represented as sticks; bottom: mean ligand RMSD over time calculated from three replicates, with KDEs of aggregated data.

MD simulations of native (*20*) and UCL2077 bound xE1R/R2E and xWT channels give kinetic insight into how the fluctuating positions of xF330 residues can modulate UCL2077 inhibition. From 500-ns MD replica trajectories of each system, the percentage of time xF330 spent inside or outside the inner vestibule for each pair of diagonally opposed protomers was calculated (Fig. 8D, red and blue bars for pair P1/P3 in xE1R/R2E and xWT, respectively, and orange and cyan bars for P2/P4 pair in xE1R/R2E and xWT, respectively). As expected, the bar graphs for both UCL2077-bound xE1R/R2E and xWT systems show that in the two UCL2077-bound protomers, the xF330 side chains remained in the inner vestibule for 100% of the trajectories (2 IN), which largely prevented the xF330 in the other pair from being able to flip. In the absence of UCL2077, since xF330 flipping was not coupled across protomers, the fraction of time spent by the residue inside the inner vestibule was calculated separately for each protomer and then aggregated. Single xF330 residues flipped toward the pore in both xE1R/R2E and xWT tetramers, with persistence values of 34.53 ± 14.63% and 25.24 ± 12.87%, respectively, while two diagonally opposed xF330 residues were found to simultaneously occupy the inner vestibule only in the xE1R/R2E state (2 IN, 14.9 ± 14.88% persistence). This conformation is similar to the UCL2077-bound structure (Fig. 8E) and is not observed in the xWT activated state. Flexible docking was then used to test whether UCL2077 can bind this xE1R/R2E native “xF330-in” conformation extracted from the MD trajectories. Docking scores for the two pockets were −8.5 and −7.9 kcal/mol, respectively (Fig. 8F, top). Additional simulations were started from the low energy poses. Although less stable than the cryo-EM binding mode, UCL2077 remained bound in the predicted pockets throughout the trajectories (Fig. 8F, bottom). These data suggest that the higher occupancy of xF330 in the flipped state in native xE1R/R2E makes this intermediate state a better target for UCL2077.

### Subtype specificity of UCL2077 for KCNQ1

No other KCNQ1 inhibitor–bound structures have been reported so far, but inhibitor complexes are available for KCNQ2 bound to Ebio3 (*30*) and KCNQ4 bound to linopirdine (*31*). Linopirdine blocks KCNQ4 channels by binding within a cytosolic cavity located beneath the inner gate, directly obstructing the ion permeation pathway (Fig. 9A) (*31*). However, linopirdine is not a specific KCNQ4 blocker as it exhibits higher affinity for all other KCNQ family channels (*32*), suggesting a similar binding site in other KCNQs as well. Ebio3 binds near the UCL2077 binding site in a pocket formed by the S4-S5L, S5, S6 helices from one protomer, and S6 helix from the adjacent protomer (Fig. 9A) and inhibits KCNQ2 channels by preventing the inner gate from opening (*30*). Our results reveal a third blocking mechanism. The KCNQ1-UCL2077 bound structures suggest an allosteric inhibitory mechanism where the S6 is bent into the conduction pathway to restrict ion permeation (Fig. 5E).

**Fig. 9.**
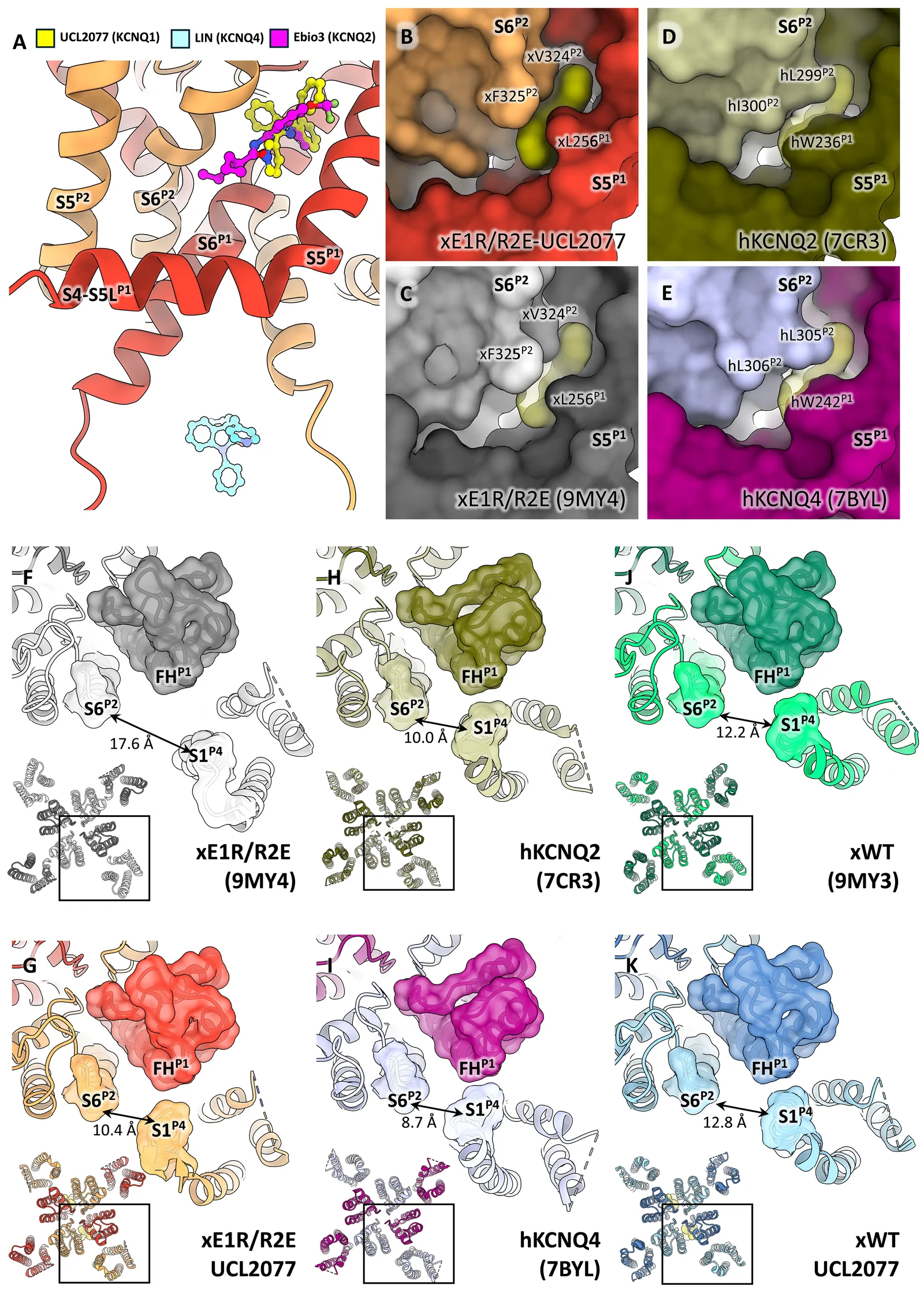
UCL2077 selectivity for the xE1R/R2E-intermediate state of KCNQ1. (**A**) KCNQ inhibitor binding sites mapped on the PD of the xKCNQ1-UCL2077 structure. The UCL2077-bound and -free protomers are colored red and orange, respectively. UCL2077 is colored yellow, Ebio3 (*30*) (KCNQ2; PDB-ID: 9IXZ) magenta, and linopirdine (*31*) (LIN, KCNQ4, PDB-ID: 7BYN) cyan. Ligands are shown as ball and stick. (**B** to **E**), surface representation of the fenestration formed between the S5-S6′ helices at the putative UCL2077 binding sites, with UCL2077 shown as a translucent yellow surface in xE1R/R2E-UCL2077 (B), native xE1R/R2E (*20*) (C), native KCNQ2 (PDB ID: 7CR3) (*67*) (D), and native KCNQ4 PDB ID: 7BYL) (*31*) (E) structures. (**F** to **K**) Domain-swapped VSD^P4^-PD^P1^ and the diagonally opposed VSD^P4^-PD^P2^ packing in native xE1R/R2E PDB ID: 9MY4) (*20*) (F), xE1R/R2E-UCL2077 (G), native KCNQ2 PDB ID: 7CR3) (H), native KCNQ4 PDB ID: 7BYL) (I), native xWT PDB ID: 9MY3) (*20*) (J), and xWT-UCL2077 (K) structures. The backbone of the UCL2077 bound PD^P1^ and its adjacent UCL2077-free S1^P4^ (VSD^P4^) or S6^P2^ (PD^P2^) are shown in dark or light surface representations, respectively. Arrows show the shortest distance between the S1^P1^ and its diagonally opposed S6^P2^ Cα atoms in xKCNQ1 (L132-W313), KCNQ2 (F112-W288) and KCNQ4 (L118-W294). In the insets, tetrameric structures are shown to facilitate orientation.

A close look at the xE1R/R2E UCL2077 binding site shows that UCL2077 fits tightly in the binding pocket, complementing its shape (Fig. 9B), which is accessible also in the native structure (Fig. 9C). However, this fenestration between the S5^P1^-S6^P2^ helices (xL256^P1^, xF325^P2^, and xV324^P2^; Fig. 9, B and C) is smaller in other KCNQs, which suggests that in these channels, the binding of UCL2077 could be sterically hindered by PD residues (Fig. 9, D and E). The KCNQ1 hL266W mutation, a residue important for drug binding in all other KCNQs (KCNQ2-W236 and KCNQ4-W242; Fig. 9, D and E), did not make the channel significantly less sensitive to UCL2077 (Fig. 3D), so that other structural differences, such as VSD-PD packing, may define the preference of UCL2077 for KCNQ1.

## DISCUSSION

UCL2077 has been used primarily as a pharmacological tool to study KCNQ channel function, with early work focusing on neuronal KCNQ subtypes despite its stronger inhibition of KCNQ1 (*21*). More recently, it was shown that UCL2077 is a potent, state-dependent inhibitor of KCNQ1, which displays higher affinity for the intermediate E1R/R2E state compared with the fully activated states and higher affinity for the intermediate state even in the presence of the accessory subunit KCNE1 compared with KCNQ1/KCNE1 channels (*19*). Its therapeutic relevance is supported by its ability in vitro to reverse the effect of clinically relevant gain-of-function, short QT syndrome mutants such as S140G (*33*). Our UCL2077-bound KCNQ1 structures reveal a well-defined binding pocket, confirmed by the electrophysiology of critical mutants, in two different activation states of the channel. Structural comparisons and kinetic studies using MD simulations reveal a mechanism for state-dependent drug sensitivity.

### UCL2077 binding

In KCNQ1 UCL2077 binds in the PD periphery (Fig. 3). This may be because in KCNQ1, as in all KCNQ channels, access to the inner vestibule through the open gate is sterically restricted. Even in the fully activated, PIP2-bound, open KCNQ1 structure, the constriction at the conserved PAG motif remains narrow [Cα-Cα distance ∼8.5 at hG345, Protein Data Bank (PDB) ID: 9VEO (*29*)], which creates a persistent bottleneck to bulky compounds. In addition, KCNQ1 lacks large S6-S6′ fenestrations, which restricts ligand access to the inner vestibule, unlike other channels (e.g., Nav) that have wider fenestrations important for lipid and drug binding (*34*). As a result, bulky molecules such as UCL2077 do not occupy the pore and instead rely on lateral binding sites.

UCL2077 breaks the fourfold symmetry of the channel and binds in two diagonally opposed protomers within a hydrophobic pocket formed by the S5-S6 helix from one protomer, with contributions from the S6 helix of the adjacent protomer (Fig. 3, A and B). Mutational studies validate the proposed binding pocket (Fig. 3, C and D), while MD simulations demonstrate stable ligand binding and sustained key interactions over the course of the simulations (Fig. 4). The xKCNQ1-UCL2077 bound structures reveal conformational changes in the S6 helix, which is bent into the conduction pathway (Fig. 5E). This bend places diagonally opposed S6 xF330 and xA331 residues in the inner vestibule, which leads to collapse and dehydration of the permeation pathway (Fig. 5G).

### VSD-PD coupling

KCNQ1 follows a multistep activation pathway with an E1R/R2E intermediate conformation that is electrophysiologically and structurally well defined. This state is absent in KCNQ2 and KCNQ3 (*17*, *20*, *35*) but may be present in KCNQ4, which has recently been shown to have a nonconducting intermediate state (*36*). In the KCNQ1 xE1R/R2E intermediate state, the S1-S2 helices are straight and detached from the adjacent FH in a manner not yet observed in other KCNQ channels [Fig. 9F and (*20*)]. The S1^P4^-FH^P1^ detached conformation reshapes the VSD-PD interface, loosens the local packing around the PD, and transiently increases accessibility to the UCL2077 binding pocket. This xE1R/R2E VSD flexibility might be a key element for UCL2077 binding in the xE1R/R2E structure, since its binding induces a clockwise movement of the VSD of the UCL2077-free protomers, bringing the S1^P4^ helix ∼7 Å closer to the diagonally opposed S6^P2^ helix (Fig. 9G, orange). In the activated xWT channel, the S1^P4^ is attached to the FH^P1^, engages tightly with the PD, and establishes a rigid VSD-PD interface (Fig. 9J). In the published native KCNQ2 (Fig. 9H) and KCNQ4 (Fig. 9I) structures, the S1 helix also leans toward the diagonally opposed S6 helix, resulting in a much tighter packing than the one observed for the xE1R/R2E intermediate state (Fig. 9F) or even the activated native and UCL2077 bound xWT KCNQ1 channels (Fig. 9, J and K). This compact arrangement could impede UCL2077 accessibility, prevent their xF330 equivalents from flipping, or limit S6 conformational changes, making KCNQ2 and KCNQ4 channels less sensitive to UCL2077. The intermediate state of KCNQ4 is nonconducting, and so the ability of the KCNQ1 gate to open in the intermediate state and its PD flexibility may make it uniquely susceptible to UCL2077. Even in the xWT-UCL2077 structure, the gate adopts a fully open conformation (Fig. 7D), albeit without the other structural changes associated with PIP2 binding and opening [S6 straightening and combining with HA helix, and CaM rotation (*10*, *28*, *29*)]. In contrast, in the xE1R/R2E-UCL2077 structure the gate dilation is intermediate between open and closed channels (Fig. 5C), which indicates that the charge-reversal mutations in the VSD have long range effects on the pore of xE1R/R2E. The ability of the gate to adopt a dilated conformation is also supported by our previously reported cryo-EM maps for native xE1R/R2E channel in the presence of PIP2 (*20*) and the xWT-UCL2077 MD simulations, which suggest that the S6 gate transitions from an open state toward the xE1R/R2E-UCL2077 intermediate state (Figs. 5E and 7G). As the channel transits from closed to open states, UCL2077 binds and stabilizes an intermediate state before it reaches the fully activated state, making the binding of UCL2077 in the intermediate state energetically favored.

### Kinetic preference for the intermediate state

MD simulations for the native xE1R/R2E channel uncovered a structural conformation similar to that with UCL2077 bound, even in the absence of UCL2077 itself (Fig. 8E). This “flipped-in” configuration did not occur in the trajectories for the xWT activated channel where only the residue in one protomer was observed to flip in the time frame sampled (Fig. 8D). The asymmetric flip of xF330 has been also observed in previous MD simulations of fully activated KCNQ1 (*37*), suggesting that the presence of two xF330 residues in the inner vestibule is limited once the channel reaches the activated state. The dual “flipped-in” conformation observed only in the MD trajectories for the native xE1R/R2E channel underlines the changed S6 kinetics between the intermediate and activated states of the channels. Consistent with the xWT xF330 limited flipping, the xWT-UCL2077 dataset was found to be heterogeneous, which led to less well-defined UCL2077 and CaM densities in comparison to xE1R/R2E-UCL2077 (Fig. 7). This may indicate less stable ligand binding, varying stoichiometry, or restricted access to the binding site, which results in a mixture of various states. Despite the heterogeneity, a common inhibitory mechanism is present for intermediate and activated states as the simultaneous presence of two xF330 side chains in the inner vestibule was resolved in cryo-EM structures of UCL2077 bound to xE1R/R2E-UCL2077 and xWT-UCL2077 (Figs. 5E and 7G).

### The UCL2077-bound structure could represent a gating state

Interpreting drug-bound channel structures benefits from placing the ligand induced conformational changes within the broader context of gating mechanisms. Notably, a collapsed pore similar to the one seen in the xE1R/R2E-UCL2077 structure has been described for the Shaker-IR (inactivation-removed) I384R mutant (*38*). This isoleucine in the Shaker S4-S5L is important for electromechanical coupling in Kv1 channels (*39*), with I384R completely uncoupling the PD from the VSD, locking the channel in a closed conformation (*38*). Compared with the WT Shaker channel, the I384R mutation leads to two hydrophobic residues, I470 and V474, swinging into the pore to collapse the permeation pathway and prevent ion conduction (fig. S15A) (*38*). This is similar to the effects of UCL2077 on the xE1R/R2E channel, where xF330, xI327, and xA331 collapse the permeation pathway (fig. S15B). Alignment between the S6 helices of the Shaker-IR-I384 and xE1R/R2E bound to UCL2077 shows that the residue equivalent to Shaker I470 in KCNQ1 is xF330 (fig. S15C), which highlights the plasticity of this region. These conformational rearrangements are likely enabled by the PVP (P473) and PAG (xP333) motifs in Shaker and xKCNQ1, respectively, as the presence of proline introduces local flexibility and helix breaking. While the physiological gating relevance of the inhibited KCNQ1 state cannot be directly inferred, its similarity with structurally and functionally characterized collapsed-pore in Shaker, together with the observation of a closely related conformation in MD simulations for the ligand-free xE1R/R2E channel, argues that this xF330 flipped state may reflect a gating state of KCNQ1.

Pausing KCNQ1 in the intermediate state, using the charge reversal mutations in the VSD, allowed for reduced sample heterogeneity and isolation of a state in which the channel was highly sensitive to UCL2077. This state appears to show greater PD flexibility that allows for xF330 flipping into the pore and the creation of a stable binding site for the ligand. Once bound, UCL2077 stabilizes a narrowed permeation pathway, which leads to eventual dehydration of the pore. Channels in the fully activated state may be less able to accommodate UCL2077 binding and so the drug is less likely to find a target in these channels. This represents a previously unreported mechanism of state-dependent KCNQ1 inhibition with potential applicability to neuronal KCNQ family channels and also provides structural templates for drug discovery for the development of new therapeutic agents for gain-of-function KCNQ1 pathologies.

### Limitations of the study

While our UCL2077-bound structures define the overall channel architecture, ligand binding, and inhibition mechanisms, they represent static snapshots and therefore do not capture the full conformational dynamics associated with ligand binding. Therefore, while the observed binding mode provides a structural framework for understanding inhibition, it may not fully reflect the dynamic binding process or alternative poses that could occur during channel gating. In addition, our cryo-EM structures, as most of the published cryo-EM structures, were determined in detergent, lacking the native membrane environment, which may influence the structure.

## MATERIALS AND METHODS

### Electrophysiology

Whole-cell patch clamp experiments were carried out as previously published (*19*, *20*). tsA201 cells (Sigma-Aldrich) were plated on glass cover slips and the next day transfected using Lipofectamine 2000 (Thermo Fisher Scientific) as per the manufacturers protocol. A 1.5 μg of channel DNA was used per dish with all the KCNQ1 constructs C-terminally linked with green fluorescent protein (GFP) to identify transfected cells. Cells were patched ∼24 to 48 hours after transfection.

The bath solution contained 135 mM NaCl, 5 mM KCl, 1 mM MgCl_2_, 1 mM CaCl_2_, 2.8 mM Na-acetate, and 10 mM Hepes (pH 7.4; adjusted with NaOH). The pipette solution contained (in mM) 130 KCl, 5 EGTA, 1 MgCl_2_, 4 Na_2_–adenosine 5′-triphosphate, 0.1 guanosine 5′-triphosphate, and 10 Hepes (pH 7.2; adjusted with KOH). All reagents were from Sigma-Aldrich except 3-(triphenylmethylaminomethyl)pyridine (UCL2077) and (3R,4S)-(+)-*N*-[3-hydroxy-2,2-dimethyl-6-(4,4,4,-trifluorobutoxy)chroman-4-yl]-*N*-methylmethanesulfonamide (HMR1556), which came from Tocris Biosciences. These were dissolved in dimethyl sulfoxide (DMSO) and stored at −20°C.

Recordings were obtained using an Axopatch 200B amplifier, Digidata 1440, and pClamp10 software (Molecular Devices). The resistance of electrodes was between 2 and 3 MΩ, data were sampled at 10 kHz and filtered at 5 kHz. Series resistance was below 10 MΩ, usually below 5 MΩ, and compensated at 70 to 75%. Cells were held at −90 mV and pulses were applied every 15 s.

### Protein expression, purification, cryo-EM grid preparation, cryo-EM data collection, and analysis

Protein expression and purification were performed as described before (*20*). Briefly, the KCNQ1 construct used for structural studies comprised a truncated *Xenopus KCNQ1* (residues 67 to 610) fused to a GFP-6×His tag to facilitate expression monitoring and affinity purification and was coexpressed with calmodulin (CaM) in tsA201 mammalian cells. Cells were transfected with a 5:1 ratio of xKCNQ1 to CaM plasmids using polyethylenimine, harvested after 48 hours, and membranes were solubilized in 1.5% (w/v) glyco-diosgenin (GDN) before affinity capture with an anti-GFP nanobody-based resin. The bound protein was eluted by on-column PreScission protease cleavage to remove the GFP tag. The cleaved xKCNQ1-CaM was concentrated and polished by size exclusion chromatography using a Superose 6 Increase 10/300 GL column (Cytiva) in buffer containing 20 mM Hepes (pH 7.2), 150 mM KCl, 4 mM EGTA, 2 mM dithiothreitol, and 0.03% GDN (*20*). The purified protein was concentrated at ∼3 mg/ml, and the sample was used immediately for cryo-EM grid preparation. For the xE1R/R2E and xWT-UCL2077 complex structures, 250 and 500 μM UCL2077 was added to the purified proteins, respectively, 20 min prior cryo-plunging. UCL2077 was dissolved in 100% (v/v) DMSO, and the final DMSO concentration in the sample was 1% (v/v).

Quantifoil R2/2 copper grids with holey carbon (Electron Microscopy Sciences) were glow-discharged (PELCO easiGlow), and using a Vitrobot Mark IV (Thermo Fisher Scientific), 2.5 μl, the sample was applied to the grids at 4°C and 100% humidity, blotted using a blot force of 9 for 3 s, and plunged in liquid ethane. Grids were screened using a 200-kV Glacios microscope (Thermo Fisher Scientific) equipped with a Falcon III detector, and data were collected on a 300-kV Titan Krios (Thermo Fisher Scientific) equipped with a Falcon 4i Direct Electron Detector and Selectris energy filter at the High-resolution Macromolecular Cryo-Electron Microscopy (HRMEM) facility at UBC. Data were collected with a pixel size of 0.96 Å, magnification of ×130,000, total dose of 50 e/Å^2^, and a defocus range between −0.5 to −2 μm. Unless otherwise stated, cryo-EM maps were visualized in UCSF ChimeraX (*40*) at a contour level of 0.0275 for xE1R/R2E-UCL2077, at 0.0951 for xWT-UCL2077, and at 0.103 for the previously published native xE1R/R2E (*20*).

For the xE1R/R2E-UCL2077 dataset, data processing was carried out using CryoSPARC V4.6.0 (fig. S1) (*41*). Gain-corrected images were patch motion–corrected followed by CTF estimation. From a total of 14,687 images, those with CTF fit worse than 6 Å were omitted, leaving 13,080 images. Particles were picked using a blob picker, and KCNQ1-CaM–like two-dimensional (2D) classes were used for template-based particle picking. A total of 3,155,378 particles were extracted using a box size of 360 pixels and Fourier-cropped to 120 pixels to minimize computational time and cleaned up with 2D classification (fig. S1A). Highest quality 2D classes were used to generate ab initio maps, and subsequently, all particles were classified with heterogeneous refinement. KCNQ1-CaM–like particles (379,656 particles) were re-extracted using a box size of 360 pixels and further cleaned up with one round of ab initio/heterogeneous refinement. Nonuniform refinement with C4 symmetry imposed was used to generate a map that was used for reference-based motion correction, followed by nonuniform refinement without symmetry imposed (236,020 particles; fig. S1, B and C). The map revealed densities that could correspond to UCL2077 in two diagonally opposed protomers (fig. S1D, inset, protomers 1 and 3). A mask encompassing the PD was used for 3D classification (fig. S1E). After 3D classification, five classes were generated and were used for nonuniform refinement without symmetry imposed (fig. S1, F and G). Classes 2, 3, and 5 displayed only low-resolution features. Class 4 at 4.35-Å resolution displayed density that could correspond to the ligand in one protomer. However, the map exhibited fragmented density near the binding site and loss of well-defined secondary structure, which limited our ability to elucidate a potential binding mode. For these reasons, class 4 was not used for further analysis. Class 1 (57,405 particles) at 4.02-Å resolution displayed high-resolution features for the PD and lower resolution, in comparison, for the VSD. Densities that correspond to UCL2077 were found only in two diagonally opposed protomers (fig. S1H, inset; protomers 1 and 3). A top view clipped at the level of UCL2077 densities highlights that the channel adopts a C2-symmetry configuration (fig. S1I). Nonuniform refinement with C2-symmetry imposed (fig. S1, J to N) improved the resolution for the PD and VSD and was used for model building. Sample maps are show in fig. S2.

For the xWT-UCL2077 dataset, data processing was carried out using CryoSPARC v4.2.1 (fig. S10) (*41*). A total of 867 images with a CTF fit worse than 5 Å were omitted leading to 12,244 micrographs. Particles were picked using a blob picker and KCNQ1-CaM–like 2D classes were used for template-based particle picking. A total of 2,252,915 particles were extracted using a box size of 360 pixels and were cleaned-up with 2D classification. Because of sample variability, C4 symmetry was imposed during ab initio and heterogeneous refinement. After two rounds of ab initio/heterogeneous refinement (fig. S10A), KCNQ1-like particles were used for ab initio and homogeneous refinement without symmetry imposed. Nonuniform refinement without symmetry imposed resulted in low-resolution maps. Therefore, C2 symmetry was imposed for nonuniform refinement (fig. S10, B and C) and produced a high-resolution map at 3.81 Å. The produced map supports diagonal binding of UCL2077 (fig. S10, D to F) and was used for model building. Sample maps are shown in (fig. S11). A systematic analysis of this heterogeneity observed in the xWT-UCL2077 dataset in comparison to the xE1R/R2E-UCL2077 can be found in the Supplementary Materials.

### Model building and refinement

For the xWT-UCL2077 and xE1R/R2E-UCL2077 complex structures, our previously published xWT-Native (PDB ID: 9MY3) and xE1R/R2E-Native (PDB ID: 9MY4) structures were used as a reference for model building (*20*). The reference structures were rigid-body fitted in the cryo-EM maps and built manually using *Coot* (v1.1.18) (*42*). The models went through rounds of manual building using *Coot* (*42*) and real-space refinement with secondary restraints using PHENIX (v2.21.2) (*43*). Model quality was assessed using Molprobity (*44*), and figures and morphs were generated using ChimeraX (v1.8 and 1.10) (*40*).

Ligand placement within the cryo-EM density was first evaluated using Rosetta EMERALD (*26*), which is designed for automated ligand-binding determination in medium-resolution cryo-EM maps. Twenty candidate poses were generated, grouped into three clusters based on binding similarity, and ranked (fig. S4A). Cluster 1 (12 of 20 poses; fig. S4B) exhibited ligand score range −81.491 to −84.908 and cluster 2 (6 of 20; fig. S4C) −81.249 to −83.146, and cluster 3 (2 of 20; fig. S4D) −73.552 and −78.197, favoring a UCL2077 conformation where the pyridine ring faces the solvent (fig. S4B). To provide an independent, density-unbiased assessment, ligand docking was also performed using AutoDock Vina (*27*) with an exhaustiveness of 32 using a search box centered on the EM-identified binding pocket. Binding poses were ranked based on the affinity and further clustered based on their pose similarity. Across both approaches, the top-ranked ligand pose was identical (fig. S5A, cluster 1), providing cross-validation of the predicted binding mode. The three Rosetta EMERALD poses were subjected to all-atom MD simulations to assess their stability (fig. S6). The highest-ranked pose maintained largely a single, well-defined conformational cluster (fig. S6D), whereas the second and the third poses sampled two distinct conformational clusters (fig. S6, H and L), indicating greater flexibility in the binding pocket. In addition, the conformation of the sidechains in pose 1 were largely maintained during the simulations (fig. S7, A and B), while for the second binding pose, an occasional flip of the side chain of xF260 occurred (fig. S7, C and D), which is not supported by the cryo-EM map. Therefore, we conclude that the first binding pose fits better in the cryo-EM map, with the highest binding energy, and stable ligand-protein interactions over the course of the simulations.

### MD simulations methods

#### Systems setup

##### Alternative ligand models bound to KCNQ1

The stability of three distinct UCL2077 conformations, each consistent with the cryo-EM map and determined using Rosetta EMERALD, was tested by MD simulations using the ligand-bound xE1R/R2E PD structure (residues G235 to Q349). The CHARMM-GUI Membrane Builder server (*45*) was used to prepare all necessary files for the simulations. Three statistically independent replicates of each system were prepared. Before production simulations, the CHARMM-GUI equilibration protocol was extended by a ∼30-ns-long trajectory with soft positional restrains on protein backbone atoms, as already done in our previous works (*46*, *47*). Then, each system replica was simulated free of restraints for 1 μs.

##### Ligand-bound full KCNQ1 TM (VSDs + PDs), xE1R/R2E and xWT

Ligand pose #1 was selected for simulations of the UCL2077-bound xE1R/R2E full TM domain (residues N95 to Q349). SWISS-MODEL was used to reconstruct missing loops (*48*), and all simulation files were generated with CHARMM-GUI. Three statistically independent replicates of the system were prepared. The equilibration protocol described above was further extended by 500 ns of minimally restrained simulation to allow hydration of the channel. In particular, the interprotomer distances between pairs of opposing G335 Cα atoms (7.9 Å) and S339 Cα atoms (10.42 Å) were not allowed to decrease using half-harmonic potentials (force constant *k* = 100 kcal·mol^−1^·Å^−2^). Successively, each replica was simulated free of restraints for 500 ns. The same protocol was employed for the xWT-UCL2077 bound structure, although in the extended equilibration stage, the distances between opposing G335 Cα atom and S339 Cα atom pairs were not allowed to decrease under 8 and 11.7 Å, respectively.

##### Ligand-free full KCNQ1 TM (VSDs + PDs), xWT

The xWT native activated structure (*20*) was also simulated, considering the whole TM domain (residues N95-Q349). The same protocol described in the “Alternative ligand models” section was used.

#### General MD settings

Each protein structure was inserted in a 1-palmitoyl-2-oleoyl-*sn*-glycero-3-phosphocholine membrane and solvated with explicit water molecules. For each system, the total charge was neutralized with a 150 mM KCl solution, yielding ∼80,000 atoms for the pore-only KCNQ1 and ∼192,000 atoms for the KCNQ1 TM. We used the NAMD2.14 software (*49*) and the CHARMM36m (*50*) force field for proteins and lipids, with a modified TIP3P water model (*51*) and NBFIX parameters as distributed in the force field. Bonded and nonbonded interaction parameters of UCL2077 were obtained from the CHARMM General force field (CGenFF) (*52*) using CHARMM-GUI. Electrostatic and van der Waals (vdW) interactions were calculated with a cutoff of 12 Å, and a switching function was applied starting to take effect at 10 Å to obtain a smooth decay (*53*). Electrostatic interactions were calculated using the Particle mesh Ewald algorithm (*54*), with a spline interpolation order 6 and a grid spacing of 1.0 Å. Covalent bonds including hydrogen atoms were constrained using the SHAKE and SETTLE algorithms (*55*, *56*), and an integration time step of 2 fs was used. Simulations were performed in the NPT ensemble with tetragonal periodic boundary conditions, at *P* = 1 atm and *T* = 310 K, using the Nosé-Hoover Langevin piston (oscillation period of 50 fs and damping timescale of 25 fs) (*57*, *58*) and a Langevin thermostat with a damping coefficient of 1 ps^−1^. The flipping of xF330 side chain in the xE1R/R2E native structure was analyzed using trajectories from our previous work (*20*). A summary of all simulations performed in reported in table S2.

#### Docking calculations

Flexible docking calculations were performed using AutoDock Vina (*27*, *59*) with the KCNQ1 xE1R/R2E closed native structure (*20*).

The receptor was obtained using the following method. First, cluster analysis was performed on 1.5-μs-long trajectories using the Gromos algorithm (*60*) and a cutoff of 2 Å in the PD region (residues G235 to Q349 for KCNQ1). The centroid of the most representative cluster was selected in which two opposing xKCNQ1 xF330 side chains point inside the pore lumen. Docking was performed with exhaustiveness parameter 32, a box of 14 Å by 14 Å by 14 Å, and the following flexible residues: xT255, xL256, xV300, xT301, xF325, xI327, xF329, and xF330 in xKCNQ1.

The best pose in each pocket was selected, and the system was simulated to assess ligand stability using the same protocol and settings described in the above “Alternative ligand models” section. Three replicas of 500 ns each were simulated for each of the two systems.

#### Calculations of the channel pore radius

The pore radius of each structure was calculated using the HOLE (*61*, *62*) program. For replicated trajectories, average and standard deviations values were obtained from aggregating the three 500 ns-long runs and taking a snapshot every 50 ns. The structures were aligned to the starting conformation using the backbone atoms. For all the HOLE calculations, AMBER van der Waals radii were adopted with a cutoff of 6 Å.

#### Structural analysis

RMSD and pore hydration were calculated using VMD (*63*) and tcl script. Cross distances were calculated using COLVAR (*64*) module distance with VMD. Cluster analysis on different binding pose was performed using the Gromos algorithm (*60*), considering RMSD on heavy atom of the ligand, and a cutoff of 1 Å. Contact analysis was performed using PROLIF (*65*) [via the MDAnalysis backend (*66*)]. To obtain smoothed representations of the probability distributions of structural observables (RMSDs and inter-residue distances), kernel density estimates were computed using an in-house Python script on values aggregated from independent replicas.

#### Molecular graphics

Molecular graphics and further analysis were performed with UCSF ChimeraX (*40*) and Visual Molecular Dynamics.

## Supplementary Materials

### The PDF file includes:

Supplementary Text Figs. S1 to S19 Legends for movies S1 to S3 Tables S1 and S2 References
