# Supplementary material for: Structural and kinetic mechanisms of state-dependent potassium channel inhibition
Source: Sci Adv. 2026 Oct 2;12(40):eaef8456. doi: 10.1126/sciadv.aef8456 (PMC13632742; doi:10.1126/sciadv.aef8456)
Supplement: Supplementary file 1 [file sciadv.aef8456_sm.pdf]

Science Advances
AAAS

Supplementary Materials for

Structural and kinetic mechanisms of state-dependent potassium channel inhibition

Efthimios Kyriakis *et al.*

Corresponding author: Luca Maragliano, l.maragliano@staff.univpm.it;
Filip Van Petegem, filip.vanpetegem@ubc.ca; David Fedida, david.fedida@ubc.ca

Sci. Adv. **12**, eaef8456 (2026)
DOI: 10.1126/sciadv.aef8456

The PDF file includes:

Supplementary Text
Figs. S1 to S19
Legends for movies S1 to S3
Tables S1 and S2
References

Other Supplementary Material for this manuscript includes the following:

Movies S1 to S3

Supplementary text

xE1R/R2E-UCL2077 and xWT-UCL2077 Variability Analyses

While the original processing pipeline successfully removed low-quality particles and resolved a clear UCL2077 binding site in two diagonally opposed protomers of the xE1R/R2E dataset (Fig. S1), the xWT-UCL2077 C2 reconstruction retained heterogeneity at the ligand-binding site, potentially due to the presence of multiple occupancy and/or conformational states (Fig. S10). To explore the conformational heterogeneity, we performed a more systematic analysis of both the xE1R/R2E-UCL2077 and xWT-UCL2077 datasets using a combination of 3D classification, and 3D variability analysis (3DVA), using cryoSPARC V4.6.0 (*41,68*).

To explore this heterogeneity, and consistent with the C2-symmetric binding mode of UCL2077, we applied C2 symmetry expansion using particles from the C2 non-uniform (NU) refinement. This converts N tetrameric particles into 2N symmetry-related particles for focused analysis. Local refinement was subsequently performed using a mask encompassing the KCNQ1 dimer to improve alignment of the expanded particles prior to variability analysis. By reducing the number of possible ligand positions from four protomer locations to two symmetry-related local sites, protomers sharing similar ligand occupancy states are more likely to be grouped together during downstream analyses. This strategy increases sensitivity to partially occupied, unbound, or intermediate states that may remain obscured in standard C1 or direct C2 reconstructions.

For the xE1R/R2E-UCL2077 dataset, the symmetry-expanded particles were subjected to local refinement using a dimer mask (Fig. S16A), yielding a reconstruction at 3.77 Å resolution (Fig. S16B). Using the locally refined particles, we next explored conformational heterogeneity focusing our analysis on ligand density, the conformation of the displaced S6 helix and the position of xF330 within the inner vestibule, dilation of the intracellular gate, and the relative arrangement of the VSDs and PDs.

3D classification identified several classes consistent with a UCL2077-bound conformation (Fig. S16C-D). Classes 1–3, 5, and 6, representing 87% of particles, displayed structural features consistent with UCL2077 binding, whereas class 4 (13%) exhibited a UCL2077-free or partially occupied S6 conformational state (Fig. S16C-D). However, all classes displayed weakened VSD density, limiting structural interpretation.

Next, we used 3DVA to determine if it could improve conformational heterogeneity. 3DVA cluster analysis similarly indicated that the majority of particles occupied a UCL2077-bound state (clusters 2-5; 90.3%), while a smaller population (cluster 1; 9.7%) displayed features compatible with a UCL2077-free state (Fig. S16E-G). Notably, this minor class retained density corresponding to both free and ligand-bound conformations near xF330, suggesting minor residual heterogeneity (Fig. S16E). Clusters 2-5 showed density supporting the asymmetric VSD-PD arrangement observed in the consensus reconstruction, although cluster 4 displayed weaker density for the PD^{P1}-VSD^{P4} attached conformation (Fig. S16E, orange). Importantly, the 3DVA demonstrated that UCL2077 occupancy correlates with the displaced S6 conformation, gate dilation, and the observed VSD-PD arrangement indicating that the xE1R/R2E-UCL2077 dataset is predominantly homogeneous. To further improve map quality, we selected subclasses exhibiting both UCL2077 density and well-resolved VSD features (clusters 2 and 5) and performed an additional round of 3DVA followed by cluster analysis, which revealed a highly homogeneous particle population

(Fig. S16H). These particles were subjected to homogeneous reconstruction, yielding a map at 3.74 Å resolution (Fig. S16I-J) compared to the initial 3.95 Å C2 NU reconstruction (Fig. S1J). UCL2077 preserves its conformation (Fig. S16K) and VSD-PD asymmetric packing remains unchanged (Fig. S16L), supporting the structural conclusions presented in this work

To further evaluate map quality and model agreement, we generated CS-Sharp (*41*), DeepEMhancer (*69*), and EMReady2 (*70*) maps. As shown in Fig. S17A, these independently processed maps consistently support the major structural features and overall model interpretation. Finally, a composite map was generated and used for refinement of the transmembrane domain of the deposited. RMSD analysis between the locally refined model and the C2-NU model showed minimal structural deviation, with overall RMSD values of 0.338 Å and 0.300 Å for the UCL2077-free and UCL2077-bound protomers, respectively (Fig. S17B), indicating that the two models are nearly identical.

To assess whether a similar strategy to that used for the xE1R/R2E-UCL2077 dataset could help resolve conformational heterogeneity and improve map quality in the xWT-UCL2077 dataset, we followed the same approach and performed C2 symmetry expansion on the C2 NU-refined xWT-UCL2077 particle stack, following local refinement (Fig. S18A-B), and subsequent 3D classification and 3DVA.

3D classification yielded classes that likely represent UCL2077-bound states (classes 1, 3, and 10; based on density for xF330 in the inner vestibule) or UCL2077-free states (classes 2, 6, 8, and 9). However, most classes appear to contain mixtures of both states. In addition, density for the VSD was generally weak across classes, although all classes support a VSD-attached conformation and open gate conformation, consistent with the C2 NU-refined reconstruction.

3DVA analysis revealed clusters that mostly represent mixed UCL2077-bound and -free states instead of clearly separated discrete states (Fig. S19A–B). Within this spectrum, certain clusters are more consistent with a UCL2077-bound like state (clusters 2, 4, and 8; 36.3%), whereas others (clusters 1 and 6; 30.2%) are more consistent with a UCL2077-free like state (Fig. S19A,C-D). This distribution suggests heterogeneous ligand occupancy, potentially with UCL2077 bound in a subset of protomers. Importantly, all clusters preserve an open-gate architecture and a VSD–PD attached conformation, consistent with the C2 NU-refined reconstruction. A third group of clusters (3, 5, 7, 9, and 10; 33.5%, Fig. S19A,C,E) exhibits weaker or less well-defined density, likely reflecting increased structural heterogeneity and/or partial occupancy. To improve the ligand-bound state, we pooled clusters 2, 4, and 8 and performed a homogeneous reconstruction, yielding a 3.75 Å map (Fig. S19F). The binding site shows improved UCL2077 density, which is now more clearly separated from the S6 helix, whereas in the C2 NU map the density appeared continuous with S6 (Fig. S19G), shown as a pink transparent surface).

3D classification and 3DVA indicate that the xWT-UCL2077 channel adopts an activated VSD conformation, an open gate, and an attached VSD-PD arrangement, consistent with the reported C2 NU refinement reconstruction. Importantly, these analyses support the overall conclusions presented in the manuscript without over-interpreting the underlying heterogeneity.

Taken together, heterogeneity analyses support that the xE1R/R2E–UCL2077 dataset is largely homogeneous, with approximately 90% of C2-symmetry expanded particles occupying a UCL2077-bound state. These results are consistent with and support the interpretations presented

in the manuscript. In contrast, for the xWT-UCL2077 dataset, variability analyses only slightly improved UCL2077 occupancy at the binding site. Instead, they suggest the presence of conformational heterogeneity, with coexisting bound- and free-like states that could not be fully separated by the approaches used here. Of note this observation is in agreement with our functional data showing the UCL2077 has higher potency for the E1R/R2E mutant compared to WT KCNQ1.

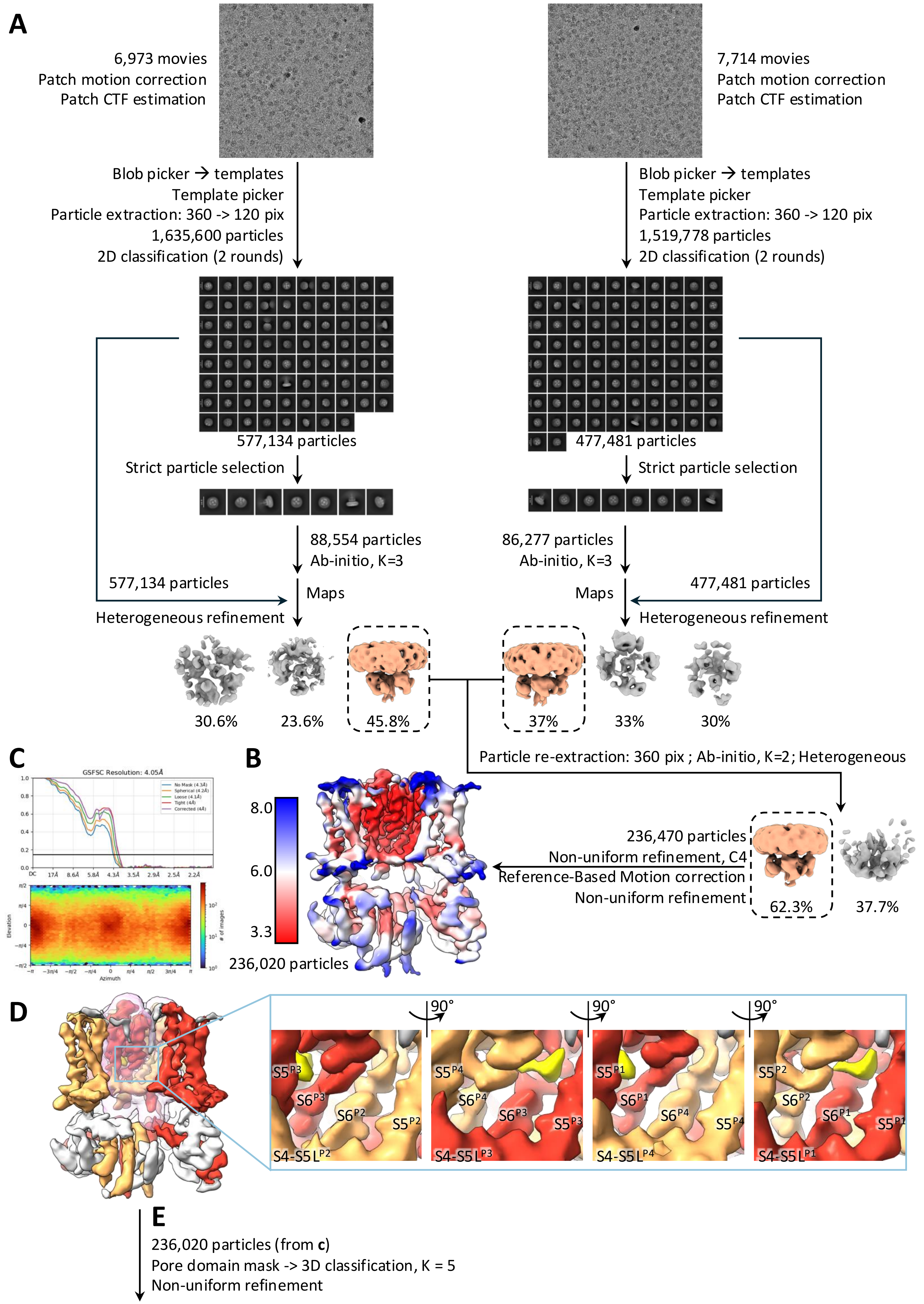

Continues on the next page

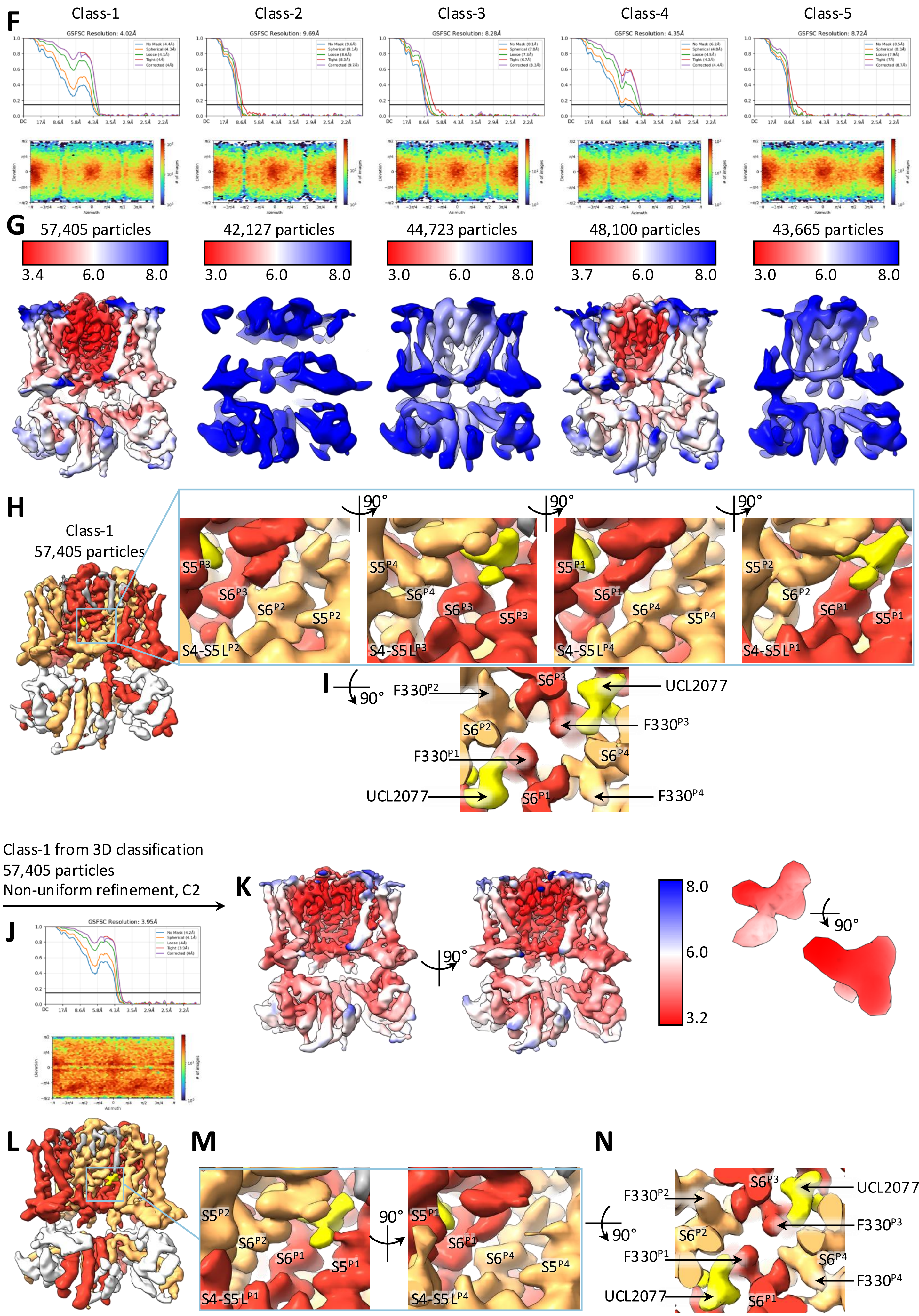
F
Class-1
Class-2
Class-3
Class-4
Class-5
GSFSC Resolution: 4.02Å
GSFSC Resolution: 9.69Å
GSFSC Resolution: 8.28Å
GSFSC Resolution: 4.35Å
GSFSC Resolution: 8.72Å
G
57,405 particles
42,127 particles
44,723 particles
48,100 particles
43,665 particles
3.4 6.0 8.0
3.0 6.0 8.0
3.0 6.0 8.0
3.7 6.0 8.0
3.0 6.0 8.0
H
Class-1
57,405 particles
90°
S5P3
S6P3
S6P2
S5P2
S4-S5LP2
S5P4
S6P4
S6P3
S5P3
S4-S5LP3
S5P1
S6P1
S6P4
S5P4
S4-S5LP4
S5P2
S6P2
S6P1
S5P1
S4-S5LP1
I
90°
F330P2
S6P3
UCL2077
F330P3
S6P2
S6P4
F330P1
UCL2077
S6P1
F330P4
Class-1 from 3D classification
57,405 particles
Non-uniform refinement, C2
J
GSFSC Resolution: 3.95Å
K
90°
8.0
6.0
3.2
L
M
S5P2
S6P2
S6P1
S5P1
S4-S5LP1
90°
S5P1
S6P1
S6P4
S5P4
S4-S5LP4
N
90°
F330P2
S6P3
UCL2077
F330P3
S6P2
F330P1
S6P4
F330P4
UCL2077
S6P1

Fig. S1. Processing pipeline for xE1R/R2E-CaM-UCL2077 dataset. Unless otherwise stated, classification and refinement were conducted without symmetry (C1). (**A**) Data processing and rounds of classification to remove particles not resembling KCNQ1. (**B**) Non-uniform (NU) refinement, and local resolution estimation (LRE) map. (**C**) FSC curves, particle distribution plot. (**D**) Same map colored by the diagonally opposed protomers in red and orange. CaM is colored white, detergent densities in gray, and potential UCL2077 densities in yellow. (**E**) A mask focused on the PD, shown as pink surface in (**D**) was used for further 3D classification followed by NU refinement for each class. (**F-G**) NU refinement FSC curves, particle distribution plots and LRE maps for each class. Class-1 exhibited continuous, well-separated density for the transmembrane helices, whereas class-4 showed fragmented density near the binding site and loss of well-defined secondary structure and was excluded from further analysis. (**H**) Class-1 colored by the diagonally opposed protomers in red and orange. CaM is colored white and detergent densities in gray. UCL2077 densities, colored yellow, were found in two diagonally opposed protomers (1 and 3) revealing a C2-symmetry binding, also shown as a top view (**I**). (**J-N**) Class-1 C2 NU refinement; (**J**) FSC curves and particles distribution plot. (**K**) LRE maps for full map and UCL2077. (**L**) and map colored by the diagonal protomers in red and coral. CaM is colored white and detergent densities gray. UCL2077 densities are colored yellow focused on the binding site from the side (**M**) and from the top (**N**).

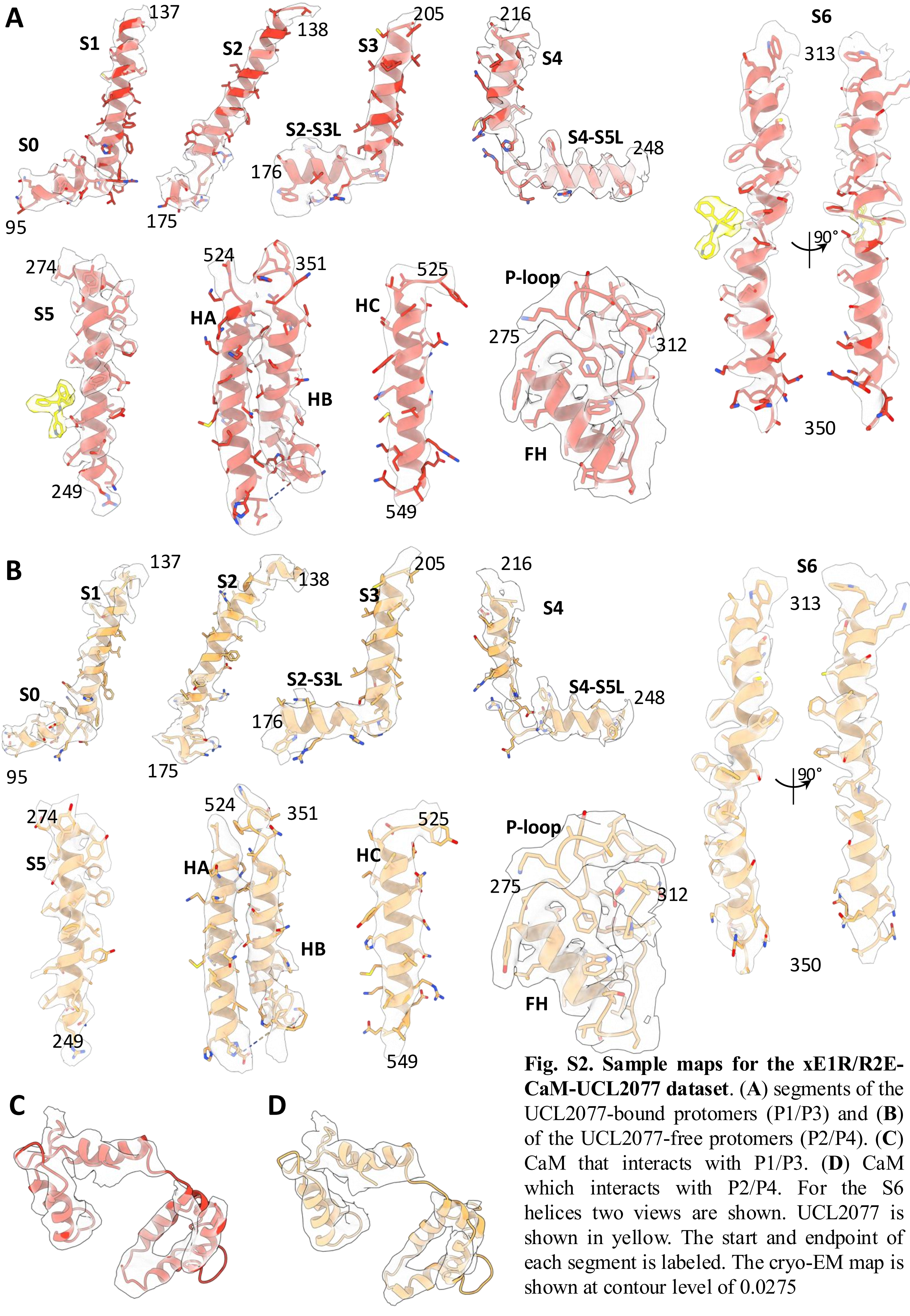

Fig. S2. Sample maps for the xE1R/R2E-CaM-UCL2077 dataset. (**A**) segments of the UCL2077-bound protomers (P1/P3) and (**B**) of the UCL2077-free protomers (P2/P4). (**C**) CaM that interacts with P1/P3. (**D**) CaM which interacts with P2/P4. For the S6 helices two views are shown. UCL2077 is shown in yellow. The start and endpoint of each segment is labeled. The cryo-EM map is shown at contour level of 0.0275

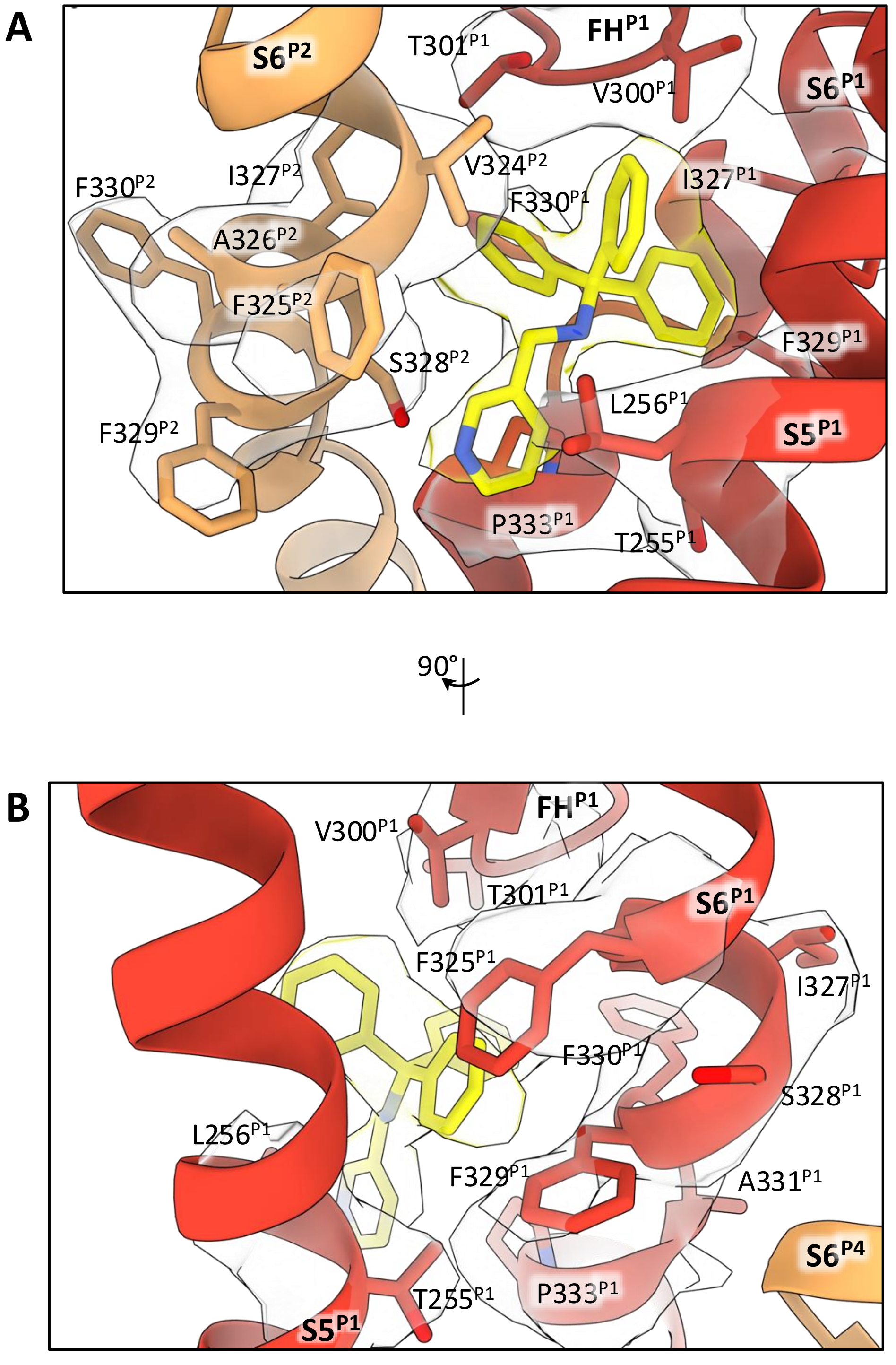

Fig. S3. Structure and map of the xE1R/R2E-CaM-UCL2077 complex and interactions with binding site residues. Adjacent protomers of xE1R/R2E are colored red/orange, and UCL2077 yellow. Cryo-EM map for binding site resides is shown as transparent surface. Cryo-EM map was clipped around the residues in the UCL2077 binding site. (**A**) Side view of the binding site formed by the S5–S6 helices of one protomer and the S6 helix of the adjacent protomer. S6 residues important for UCL2077 binding are also shown in the adjacent S6^{P2} (xV324-xF330) to highlight the quality of the map in this region. (**B**) Side view of the complex channel structure rotated 90° relative to (**A**) to reveal additional interacting residues not visible in the initial orientation. The xE1R/R2E-UCL2077 cryo-EM map is shown at contour level of 0.0275.

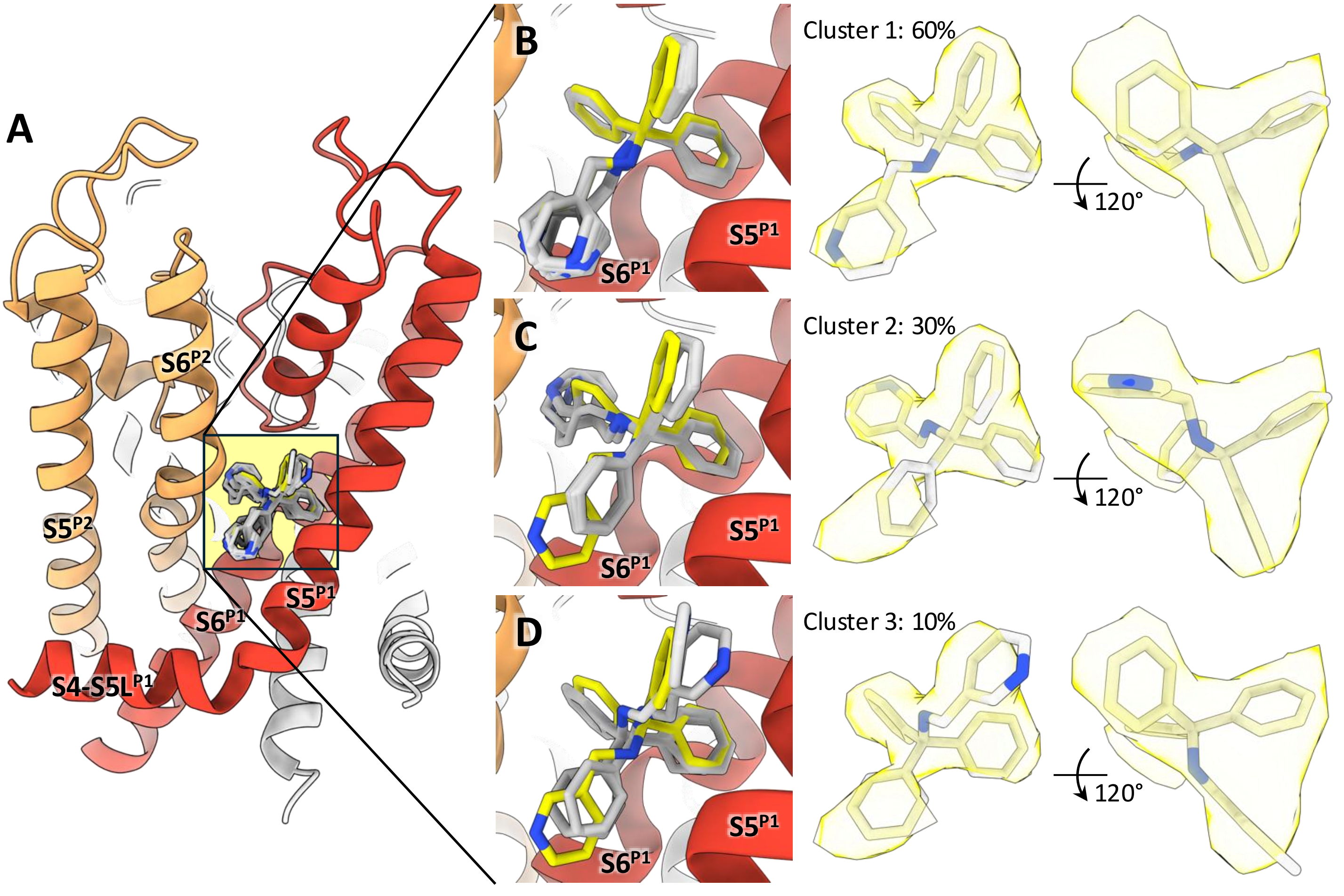

Fig. S4. UCL2077 binding pose determination using Rosetta EMERALD. The xE1R/R2E-UCL2077 protomer that has UCL2077 bound in its PD is colored red (P1), the protomer that does not bind UCL2077 in its PD is colored orange (P2), and their diagonal protomers are colored gray. UCL2077 as docked in the cryo-EM structure is colored yellow and the Rosetta EMERALD poses are in gray. (**A**) All Rosetta Emerald generated ligand poses in the binding pocket. (**B-D**) Comparison between cluster 1 (**B**), cluster 2 (**C**), and cluster 3 (**D**) with the cryo-EM pose. The fit of the top-ranked Rosetta EMERALD poses in the cryo-EM map in shown on the right in two different view. The xE1R/R2E-UCL2077 cryo-EM map is shown at contour level of 0.0275.

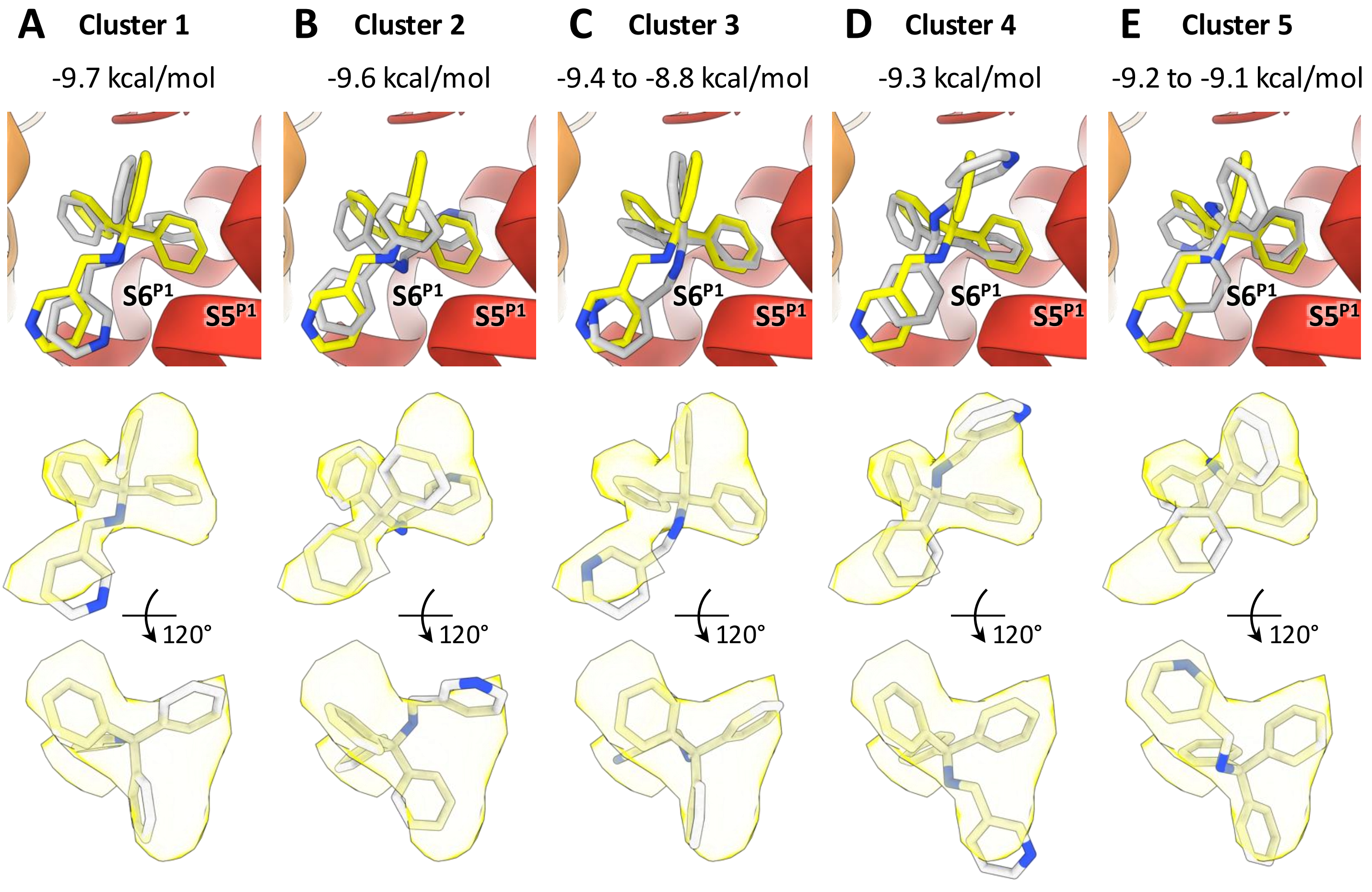

Fig. S5. AutoDock-generated binding poses and comparison with the cryo-EM structure. The xE1R/R2E-UCL2077 protomer that binds UCL2077 in its PD is colored red (P1), the protomer that does not bind UCL2077 in its PD is colored orange (P2). UCL2077 as docked in the cryo-EM structure is colored yellow for reference, and the AutoDock-generated poses in gray. (**A-E**) **Top** representative poses from each cluster in comparison with the ligand pose observed in the cryo-EM structure. **Bottom**: the representative pose from each cluster displayed in the cryo-EM map without any post-docking fitting or refinement. Two different view are shown. The xE1R/R2E-UCL2077 cryo-EM map is shown at contour level of 0.0275.

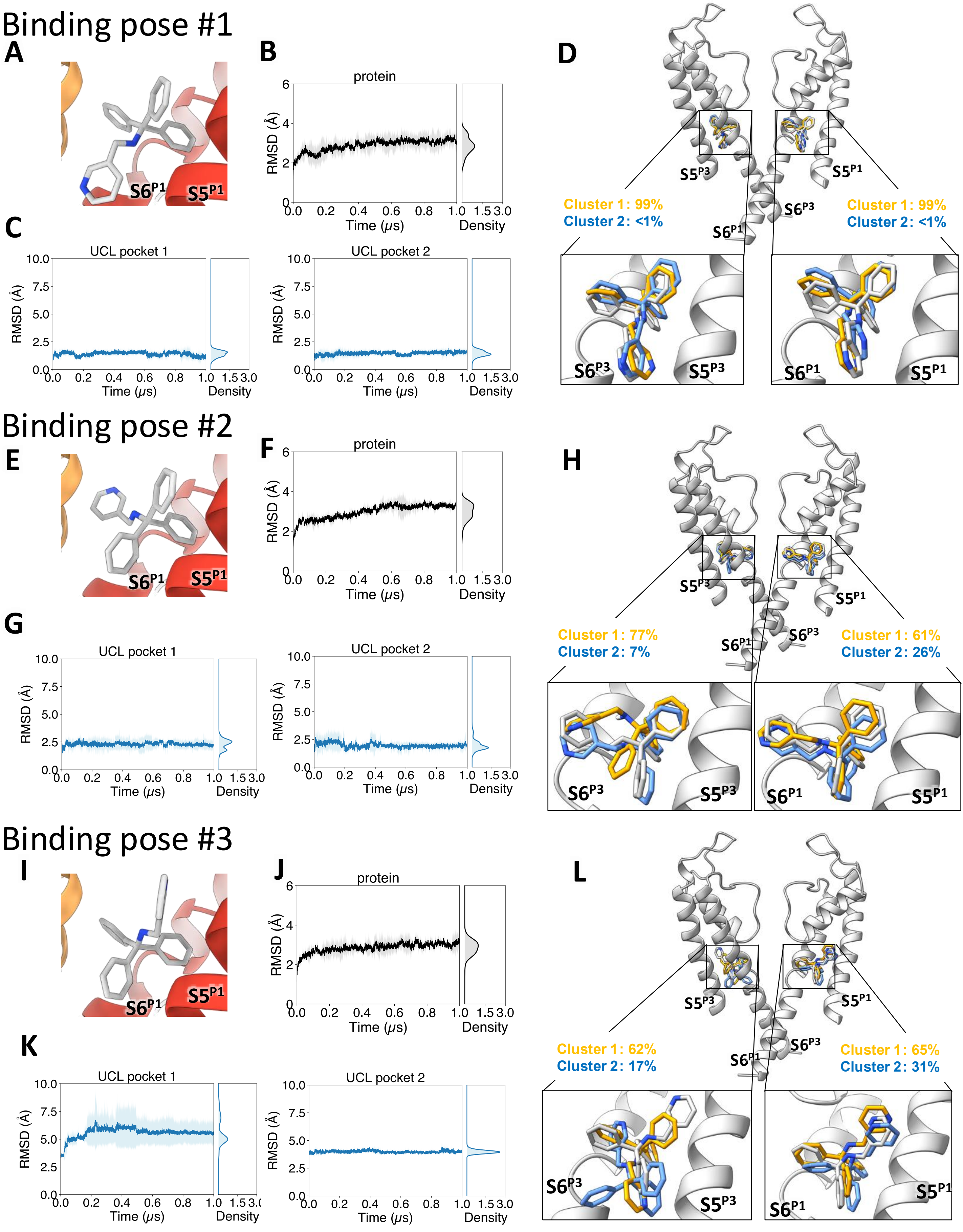

Fig. S6. MD simulations of the three Rosetta EMERALD poses. (**A,E,I**) Starting Rosetta EMERALD pose for binding poses 1, 2 and 3, respectively. (**B,F,J**) Protein backbone RMSD during simulations, with KDEs from aggregated data. (**C,G,K**) Ligand RMSD in each pocket during 1 μs MD simulations for binding poses 1, 2 and 3, with KDEs from aggregated data. (**D,H,L**) Cluster centroids of ligand from simulations of binding pose 1, 2 and 3. The first and second most populated clusters are colored orange and blue, respectively, while the starting pose is grey.

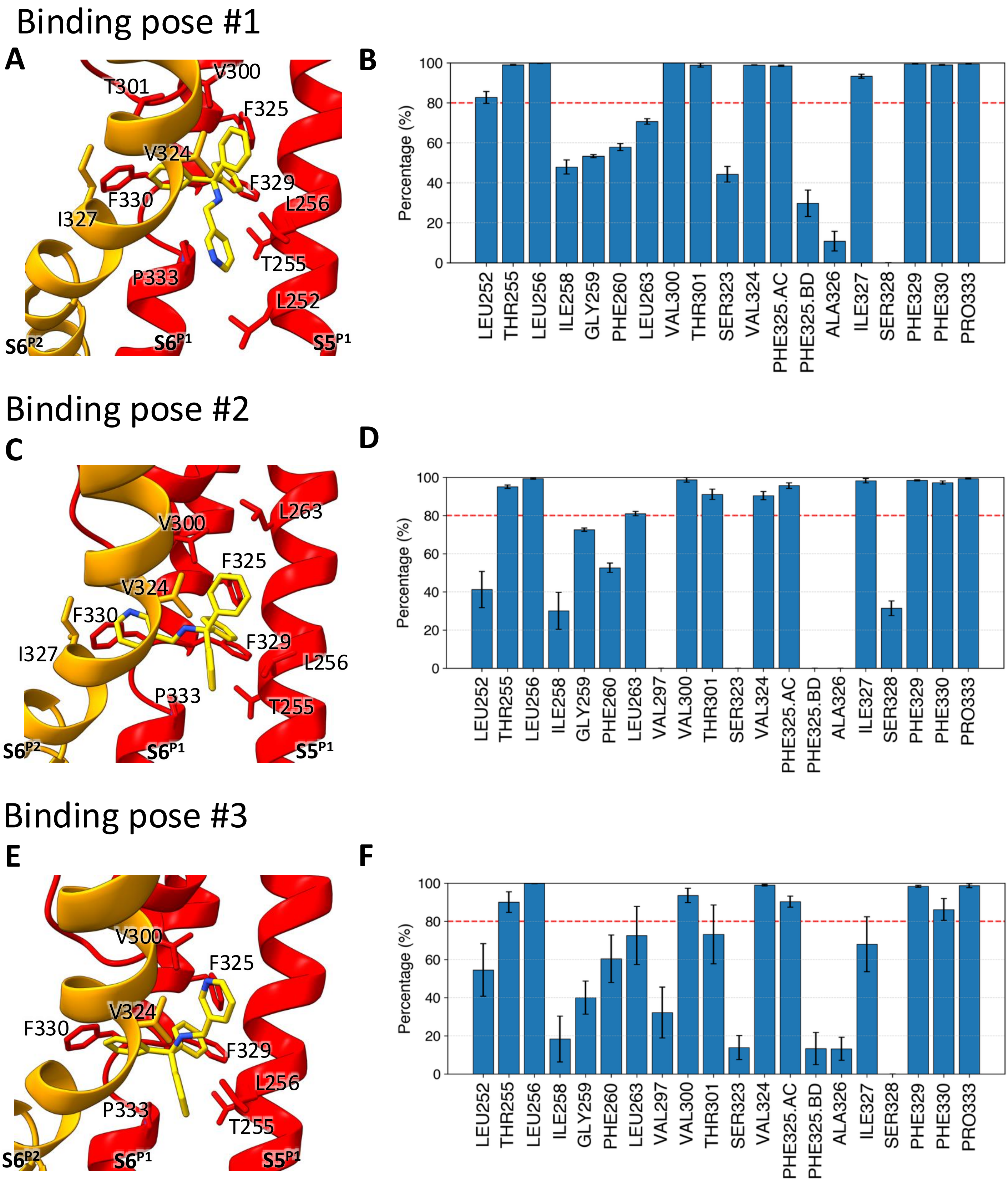

Fig. S7. Contact Analysis of the three most favorable drug binding poses. (**A,C,E**) Residues contacting the ligand with a persistence > 80%, in binding poses 1, 2 and 3, respectively. (**B,D,F**) Persistence (%) of ligand-residue interactions throughout the simulations for binding poses 1, 2 and 3.

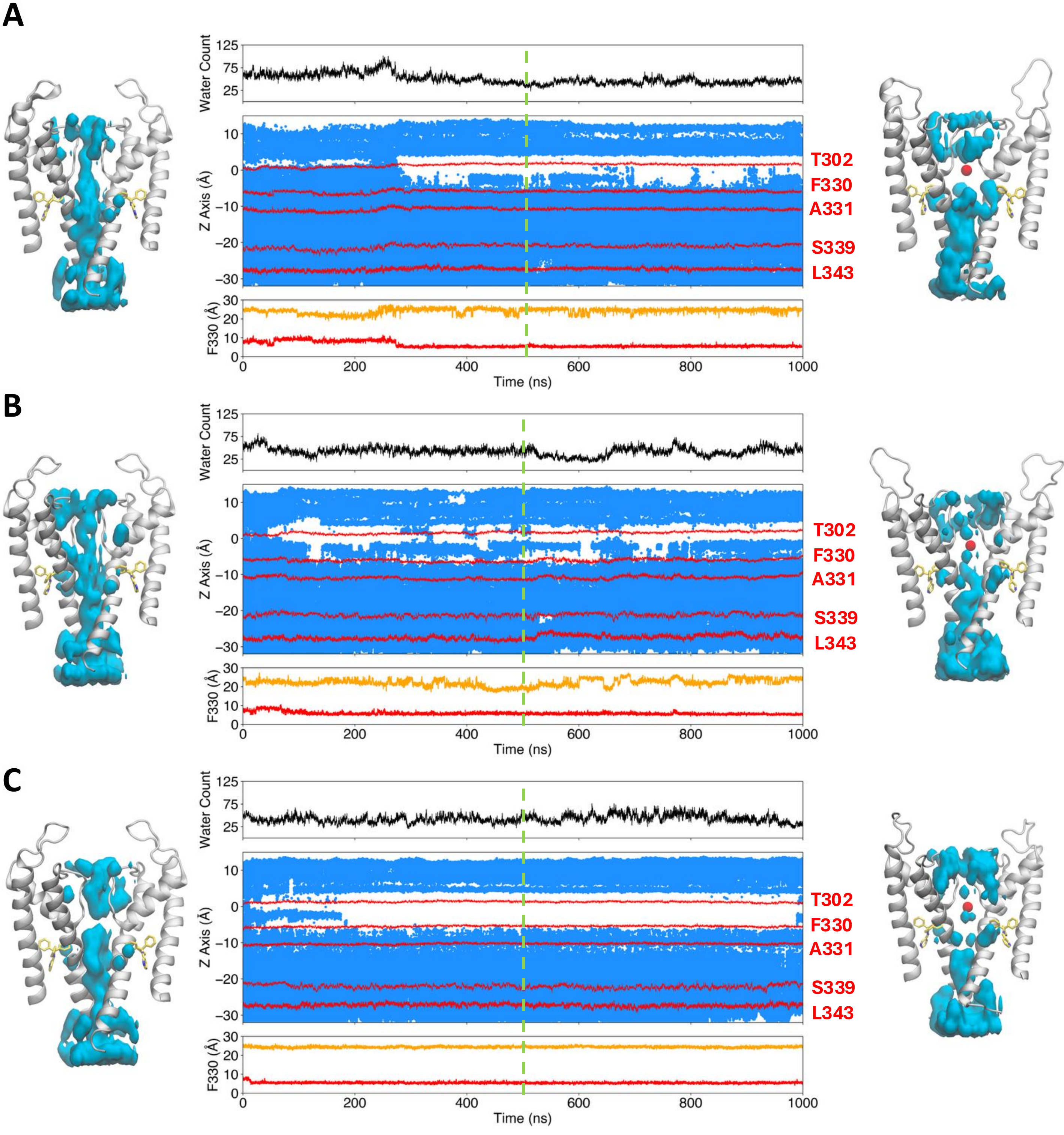

Fig. S8. xE1R/R2E-UCL2077 pore hydration. (**A,B,C**) Pore hydration of three simulation replicas. On the top the water count into the pore region. The center plot represent water molecule z-coordinates within the channel pore using blue dots. The five red lines are the z coordinates of the side chains of the pore residues (xT302, xF330, xA331, xS339, xL343). On the bottom the distances between xF330 rings of opposite protomers. At the sides the 3D representation of the water column, first step at left, last step at right. Green dashed lines divide the equilibration (0-500 ns) from the production phase (500-1000 ns).

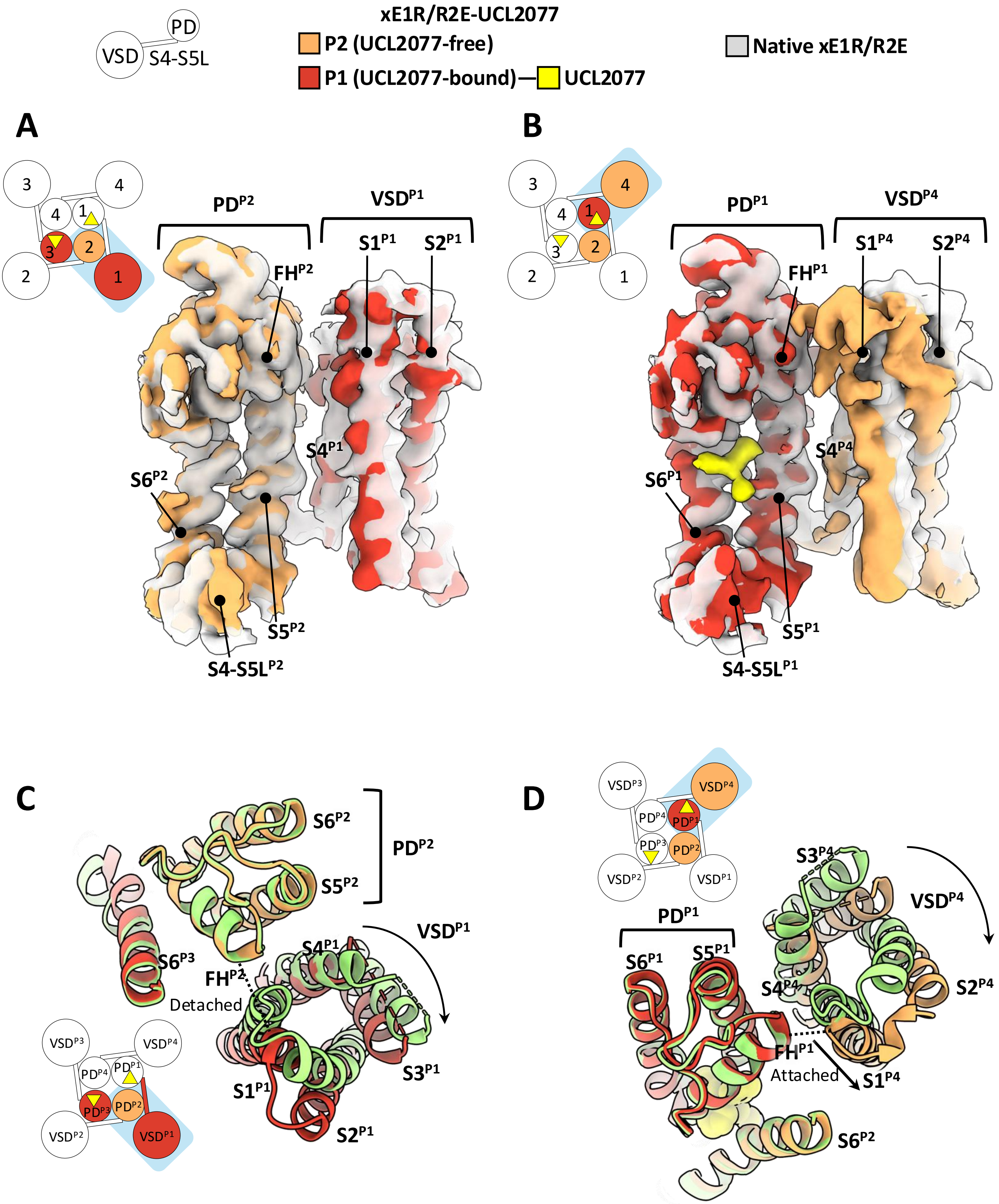

Fig. S9. Comparison between the domain-swapped PD-VSD UCL2077-bound and -free pairs. xE1R/R2E native structure is shown for reference in white, xWT in green, UCL2077 PD-bound protomers red (P1 and P3), PD-free protomers orange (P2 and P4), and UCL2077 yellow. (**A-B**) Side view of the cryo-EM maps for the domain-swapped pairs. The xE1R/R2E-UCL2077 cryo-EM map is shown at contour level of 0.0275, and native xE1R/R2E map at 0.103. (**A**) Comparison focused on the PD-bound VSD (P1, red) and the adjacent UCL2077-free PD (P2, orange). (**B**) Comparison focused on the UCL2077-bound PD (P1, red) and the adjacent PD-free VSD (P4, orange). (**C-D**) **Comparison between xE1R/R2E-UCL2077 and native xWT structures.** Top views of the same configurations as in panels (**A**) and (**B**) is shown.

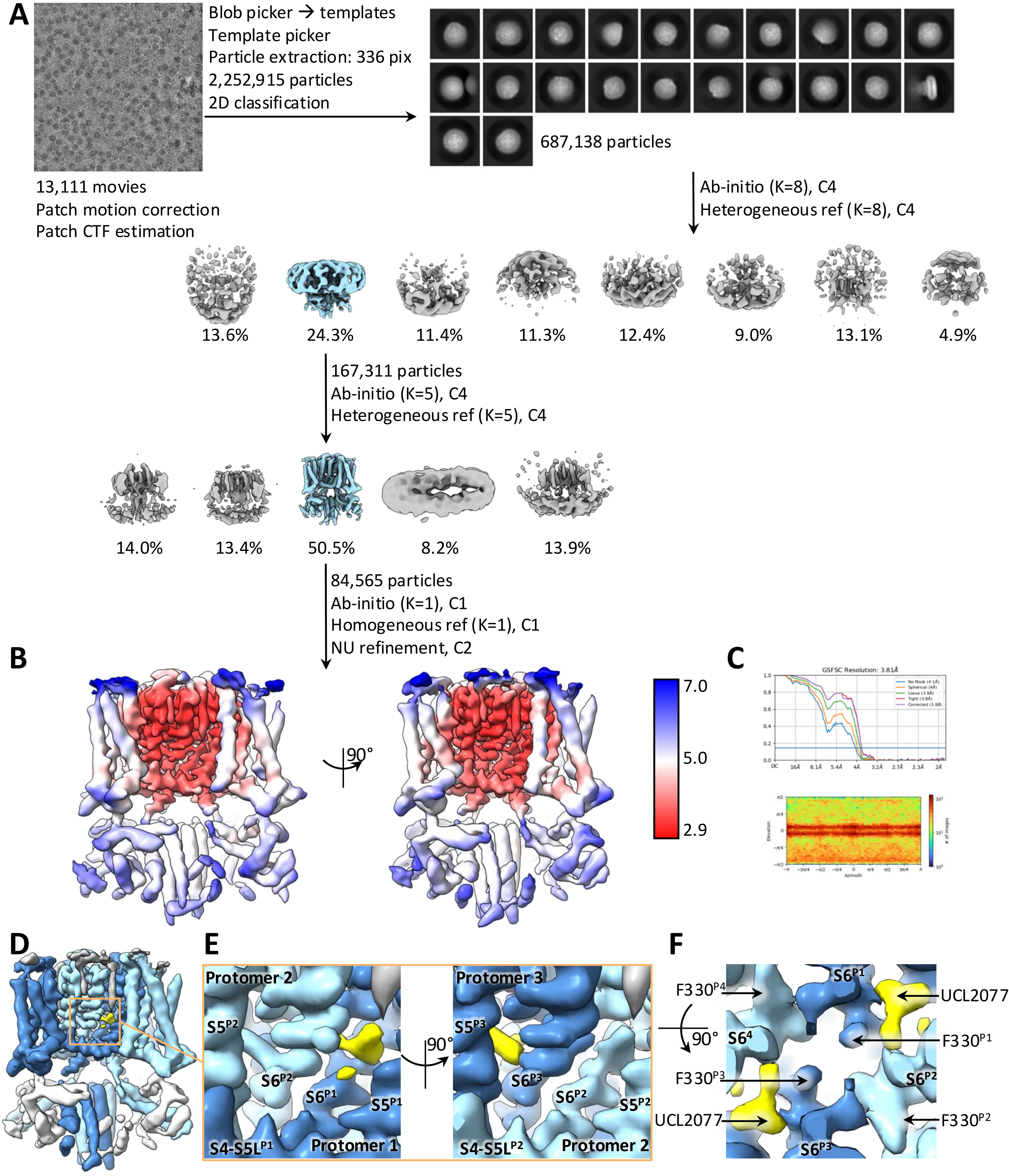

Fig. S10. Processing pipeline for xWT-CaM-UCL2077 dataset. (**A**) Data processing and rounds of classification to remove particles not resembling KCNQ1. (**B**) Non-uniform (NU) refinement map colored based on the local resolution estimation, and **C,** FSC curves and particle distribution plot. (**D**) Same map colored by the diagonal protomers in blue (UCL2077 bound) and cyan (UCL2077 free). CaM is colored white and detergent densities gray. UCL2077 densities are colored yellow focused on the binding site from the side (**E**) and from the top (**F**).

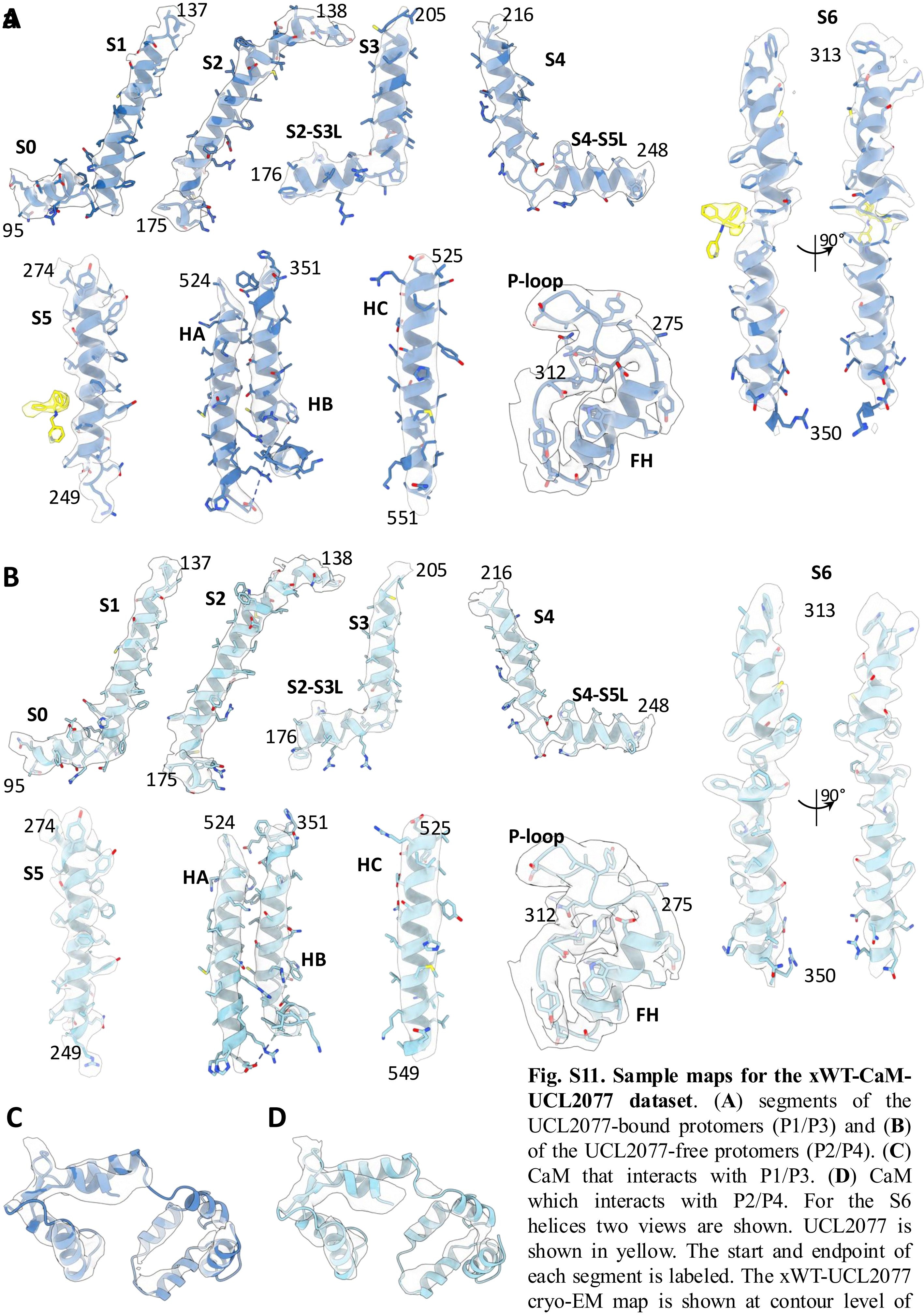

Fig. S11. Sample maps for the xWT-CaM-UCL2077 dataset. (**A**) segments of the UCL2077-bound protomers (P1/P3) and (**B**) of the UCL2077-free protomers (P2/P4). (**C**) CaM that interacts with P1/P3. (**D**) CaM which interacts with P2/P4. For the S6 helices two views are shown. UCL2077 is shown in yellow. The start and endpoint of each segment is labeled. The xWT-UCL2077 cryo-EM map is shown at contour level of 0.0951.

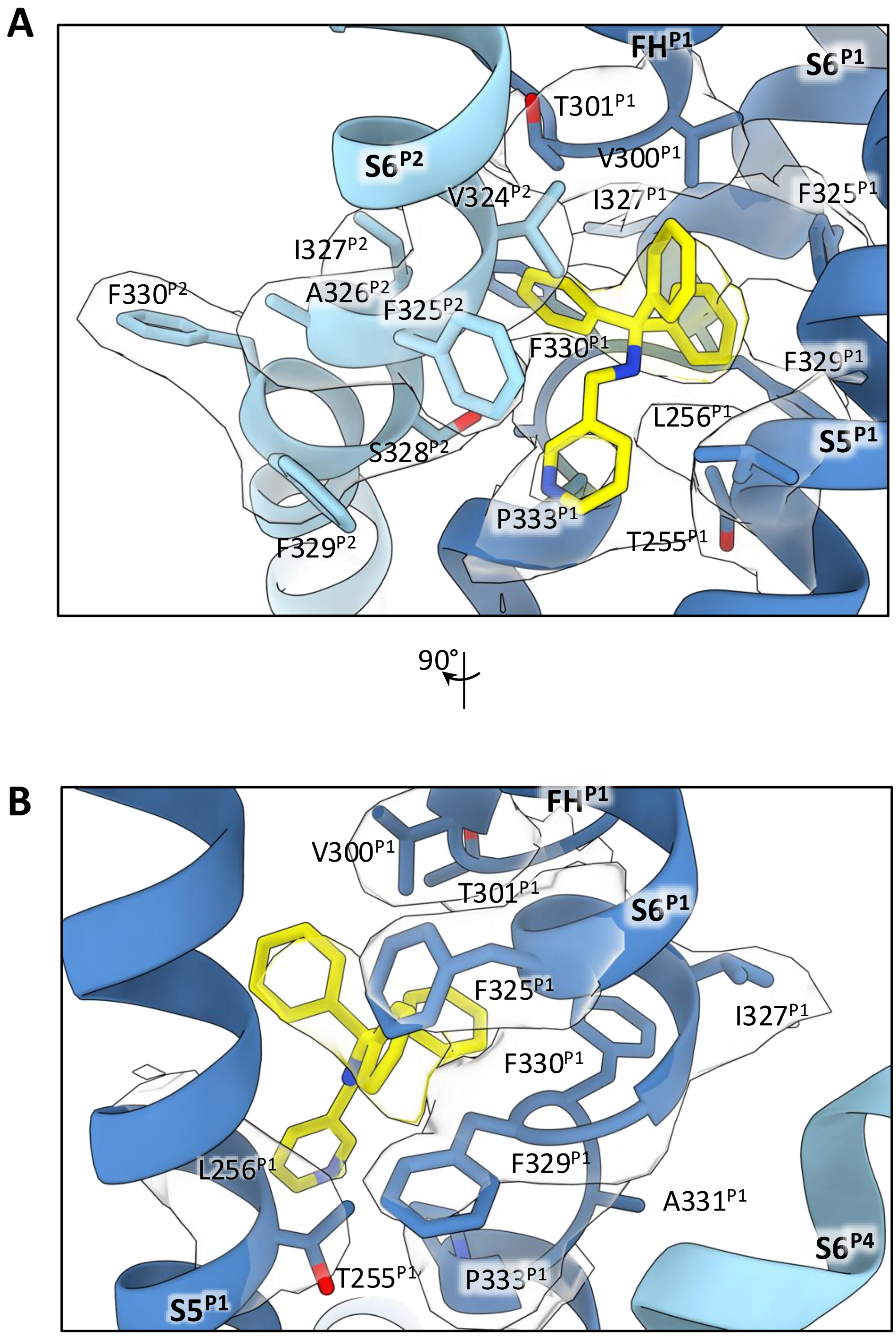

Fig. S12. Structure and map of the xWT-CaM-UCL2077 complex and interactions with binding site residues. Adjacent protomers of xWT are colored blue/cyan, and UCL2077 yellow. Cryo-EM map for binding site resides is shown as transparent surface. Cryo-EM map was clipped around the residues in the UCL2077 binding site. (**A**) Side view of the binding site formed by the S5–S6 helices of one protomer and the S6 helix of the adjacent protomer. S6 residues important for UCL2077 binding are also shown in the adjacent S6^{P2} (xV324-xF330) to highlight the quality of the map in this region. (**B**) Side view of the complex channel structure rotated 90° relative to a to (**A**) reveal additional interacting residues not visible in the initial orientation. The xWT-UCL2077 cryo-EM map is shown at contour level of 0.0951.

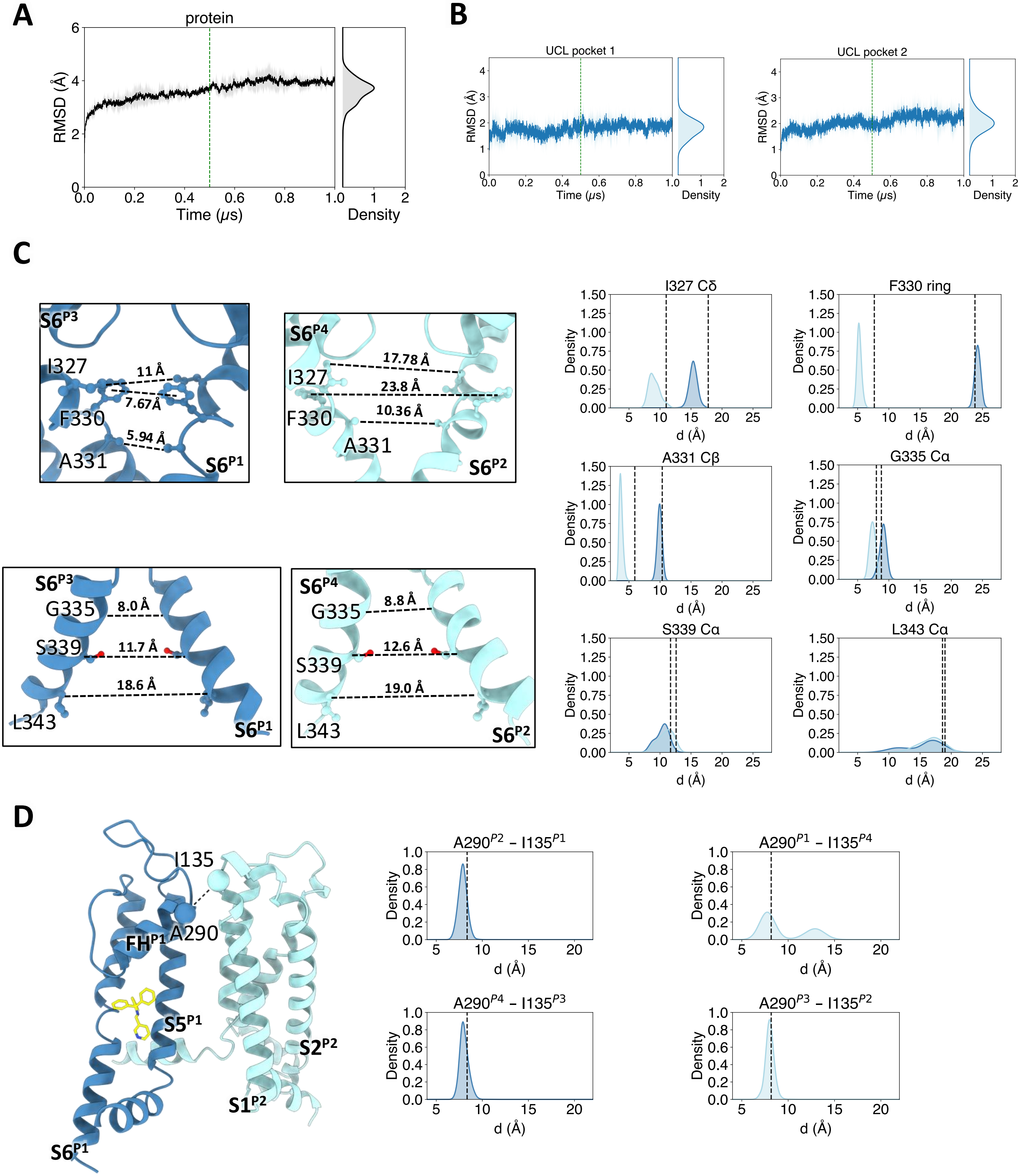

Fig. S13. xWT-UCL2077 analysis. (**A**) Protein backbone RMSD over time, averaged over three replicates of 1 µs each, with KDEs from aggregated replicas. The dashed green line indicates the end of equilibration runs (500 ns). (**B**) Ligands RMSD over time, averaged over three replicates of 1 µs each, with KDEs from aggregated replicas. (**C**) Right: KDEs of distances between facing protomers at the inner vestibule (xI327Cδ, xF330 ring and xA331Cβ) and gate region (xG335Cα, xS339Cα and xL343Cα). Cryo-EM structure reference values are shown as dashed black line. Left: 3D representations of the distances in the Cryo-EM structure. (**D**) Right: KDEs of distances between PDs (xA290Cα) and VSDs (xI135Cα) for the four protomers. Reference value of the Cryo-EM is shown as dashed black line. Left: 3D representation of the distances in the Cryo-EM structure.

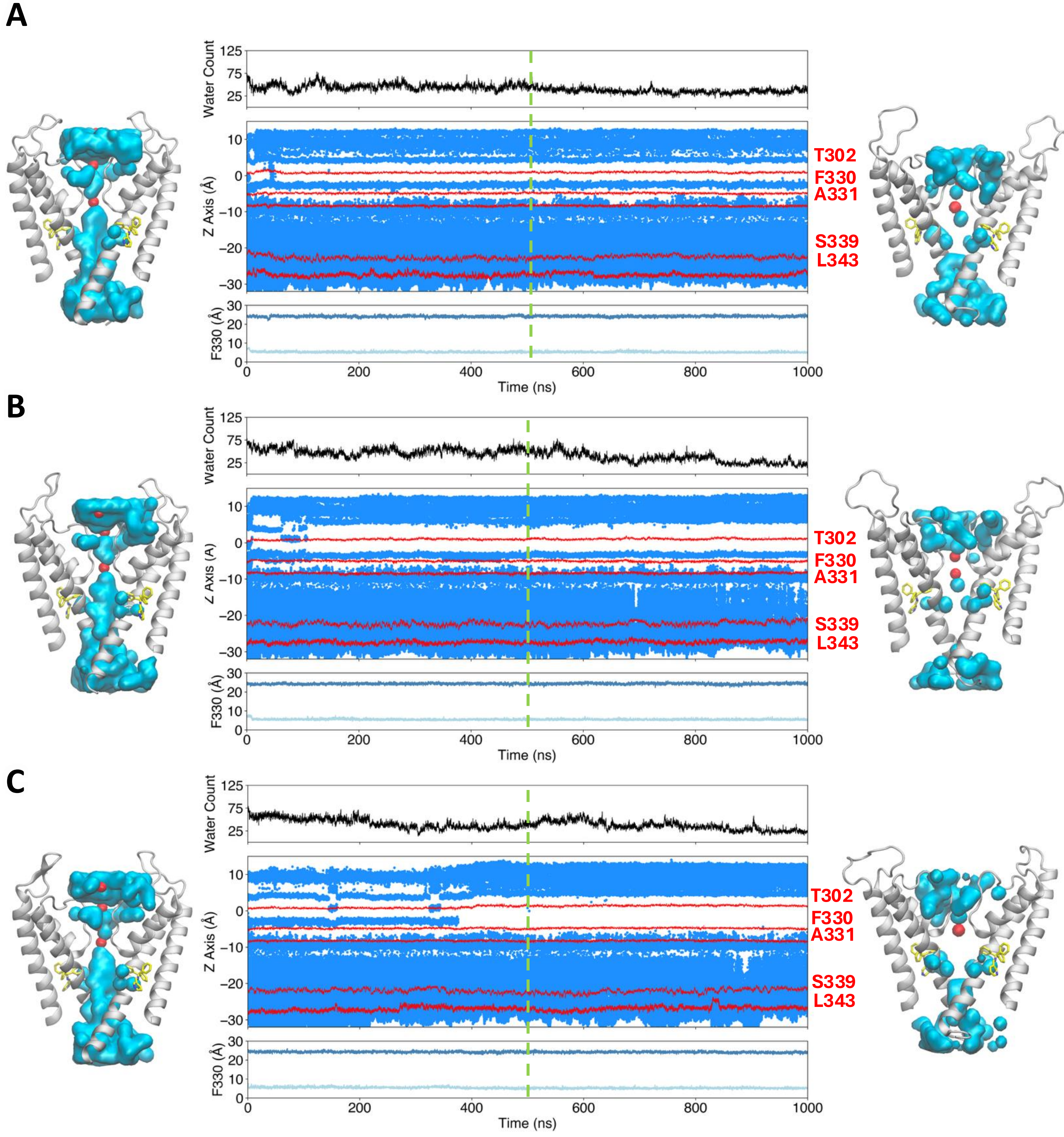

Fig. S14. xWT-UCL2077 pore hydration. (**A,B,C**) Pore hydration of three simulation replicas. On the top the water count into the pore region. The center plot represent water molecule z-coordinates within the channel pore using blue dots. The five red lines are the z coordinates of the side chains of the pore residues (xT302, xF330, xA331, xS339, xL343). On the bottom the distances between xF330 rings of opposite protomers. At the sides the 3D representation of the water column, first step at left, last step at right. Green dashed lines divide the equilibration (0-500 ns) from the production phase (500-1000 ns).

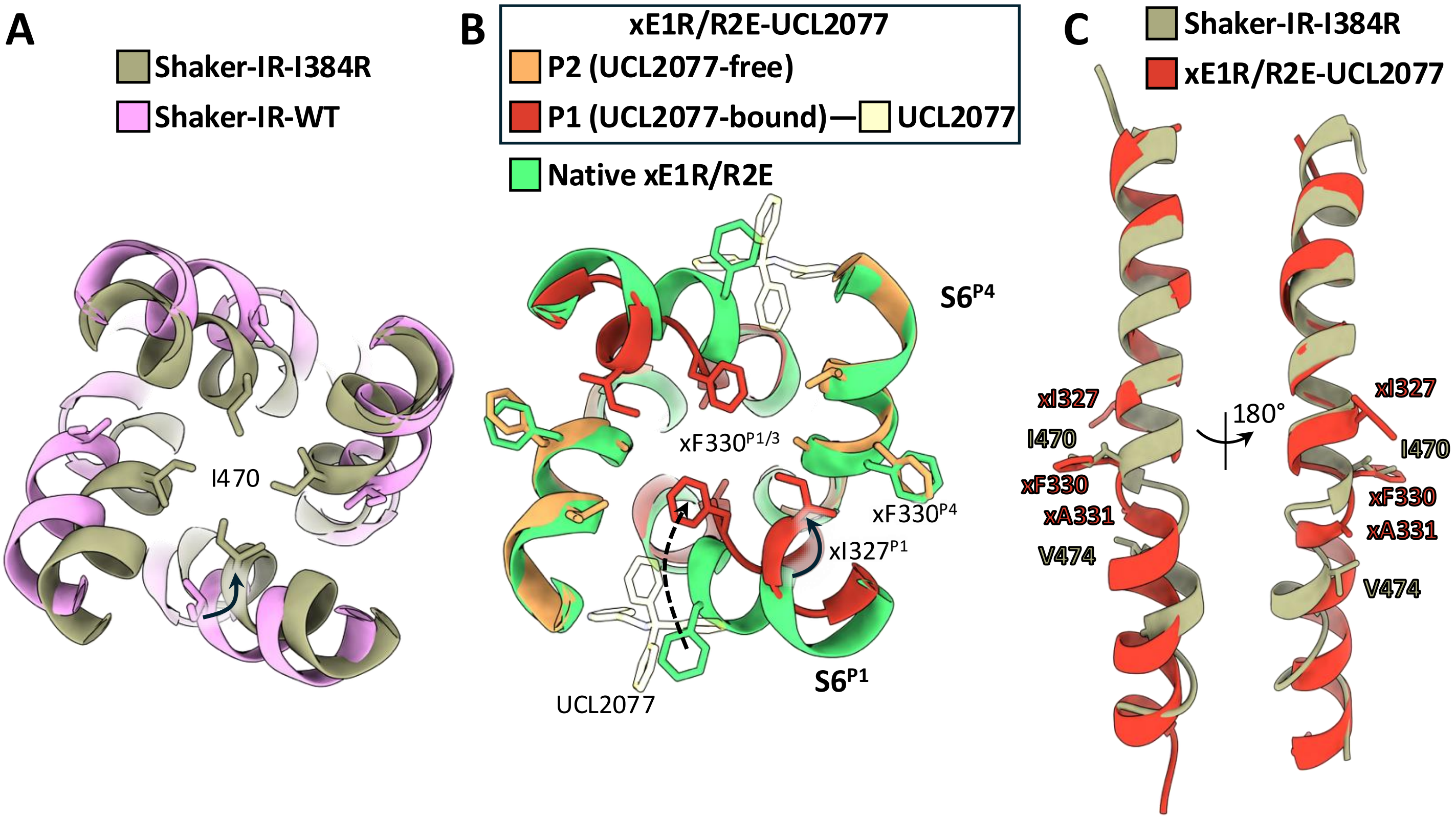

Fig. S15. Comparison between UCL2077 bound xE1R/R2E and Shaker-I384R closed state channel. Diagonally opposed protomers of xE1R/R2E are colored red/orange, and Shaker-IR-I384R tan. UCL2077 is colored yellow and showed in transparency for clarity. The native structures of xE1R/R2E and Shaker-WT are shown for reference in green and pink, respectively. (**A**,**B**) top view of the superposition of Shaker-IR-I384R (PDB-ID: 9OIC) onto Shaker-IR-WT (PDB-ID: 7SIP) (**A**) and xE1R/R2E-UCL2077 onto xE1R/R2E (**B**). (**C**) superposition of the S6 helices of the Shaker-IR-I384R and the UCL2077 bound xE1R/R2E.

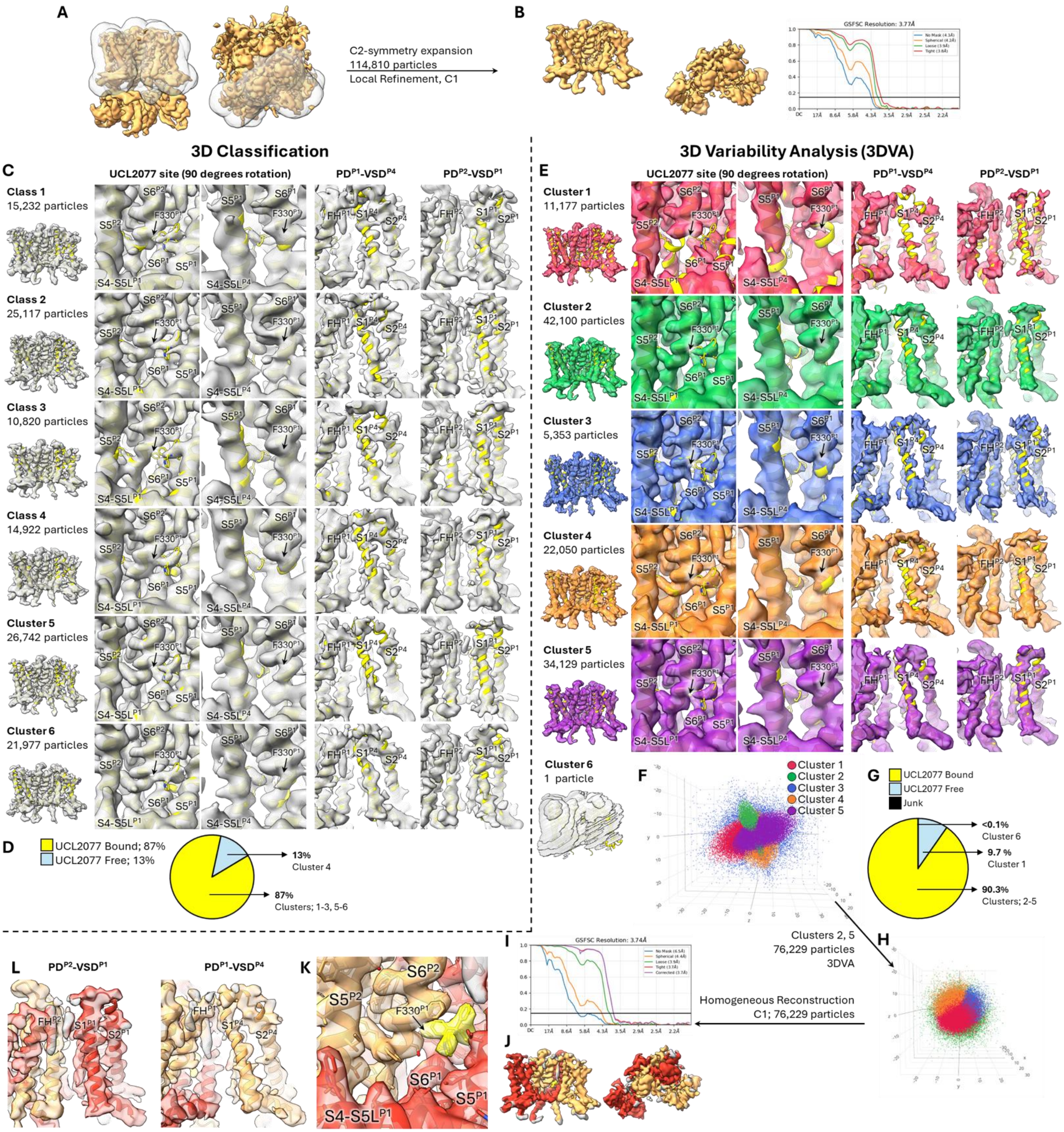

Fig. S16. xE1R/R2E-UCL2077 dataset 3D classification and 3D variability analyses. (**A**) C2 non-uniform (NU) reconstruction and mask used for local refinement. (**B**) Local refinement reconstruction and corresponding FSC plot. (**C**) 3D classification classes. (**D**) Pie chart summarizing the proportion of particles assigned to the different conformational states identified by 3D classification. (**E**) 3DVA clusters. (**F**) Clustered scatter plots of the 3DVA particle distribution. (**G**) Pie chart summarizing the proportion of particles assigned to the different conformational states identified by 3DVA. (**H**) Clustered scatter plots of the 3DVA particle distribution following a second round of 3DVA. (**I**) FSC plot of the homogeneous reconstruction and (**J**) homogeneous reconstruction map. (**K**) xE1R/R2E-UCL2077 homogeneous reconstruction at the level of the UCL2077 binding site. (**L**) VSD-PD packing arrangement in the xE1R/R2E-UCL2077 reconstruction.

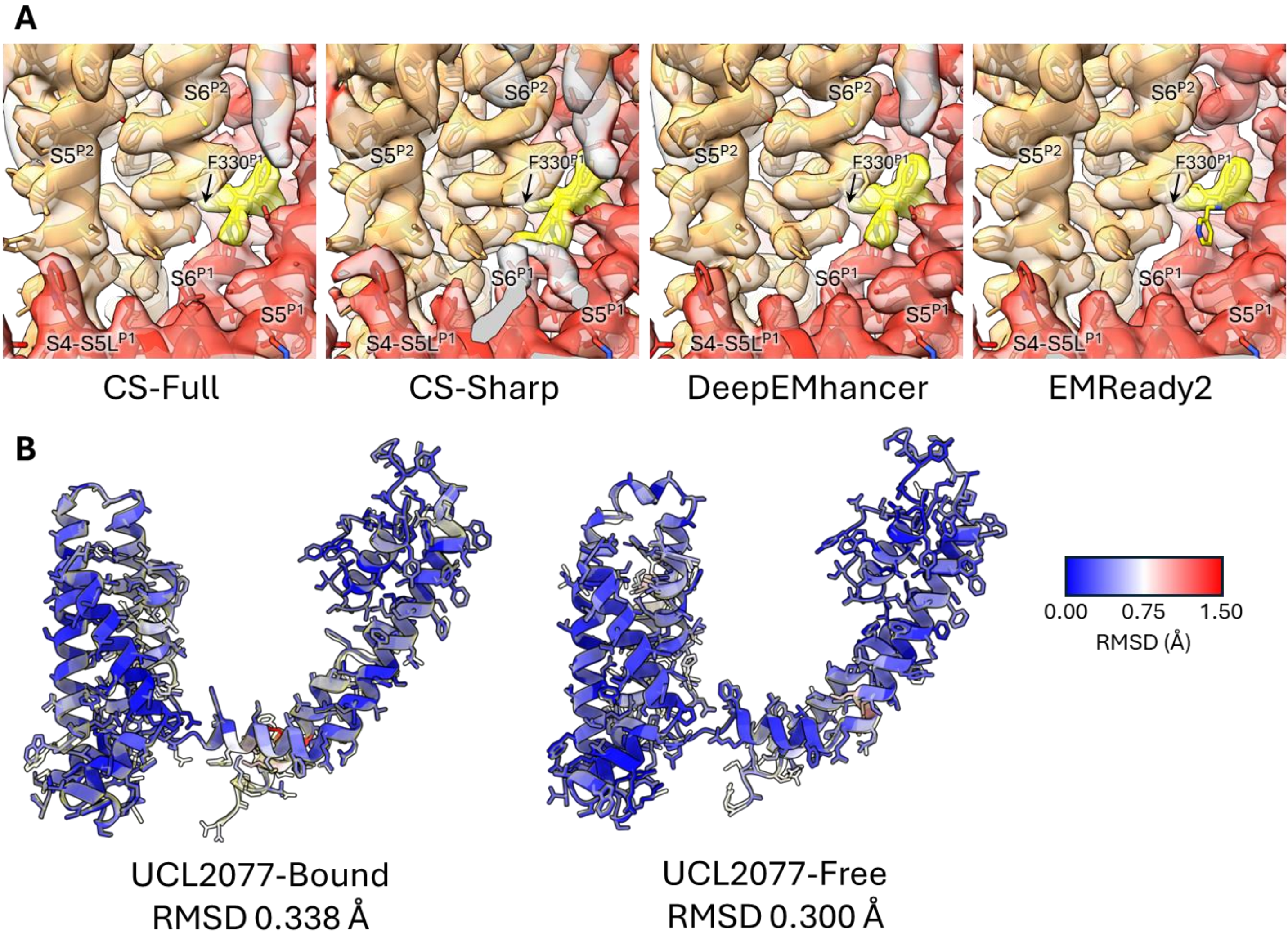

Fig. S17. xE1R/R2E-UCL2077 reconstruction after 3DVA. (**A**) Full homogeneous reconstruction map and post-processed maps. (**B**) RMSD-based coloring of the UCL2077-bound and UCL2077-free protomers relative to the reference C2-NU model. The reference C2-NU structure is shown as a transparent yellow overlay for comparison.

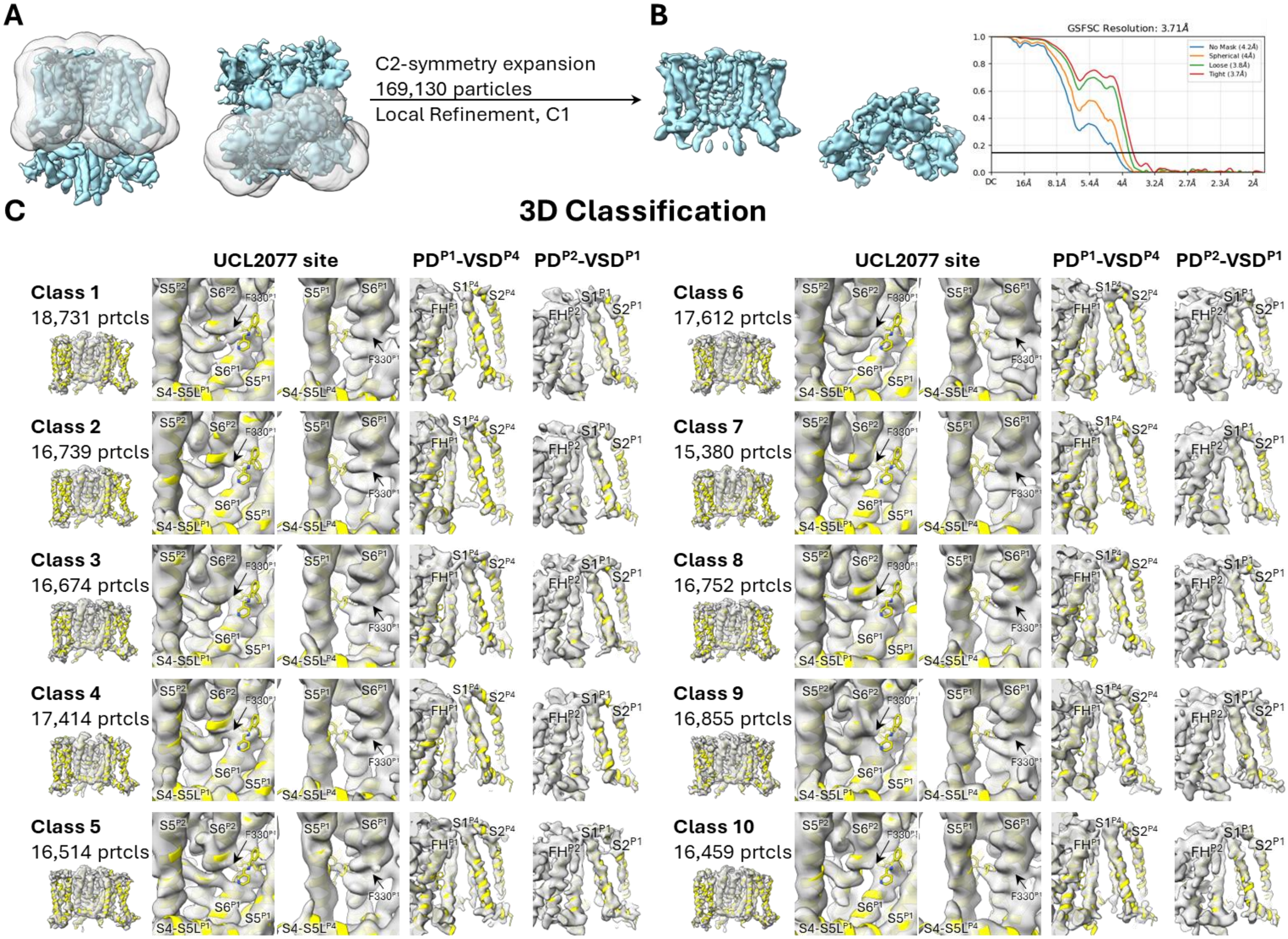

Fig. S18. xWT-UCL2077 dataset 3D classification analysis. (**A**) C2 non-uniform (NU) reconstruction and mask used for local refinement. (**B**) Local refinement reconstruction and corresponding FSC plot. (**C**) 3D classification classes.

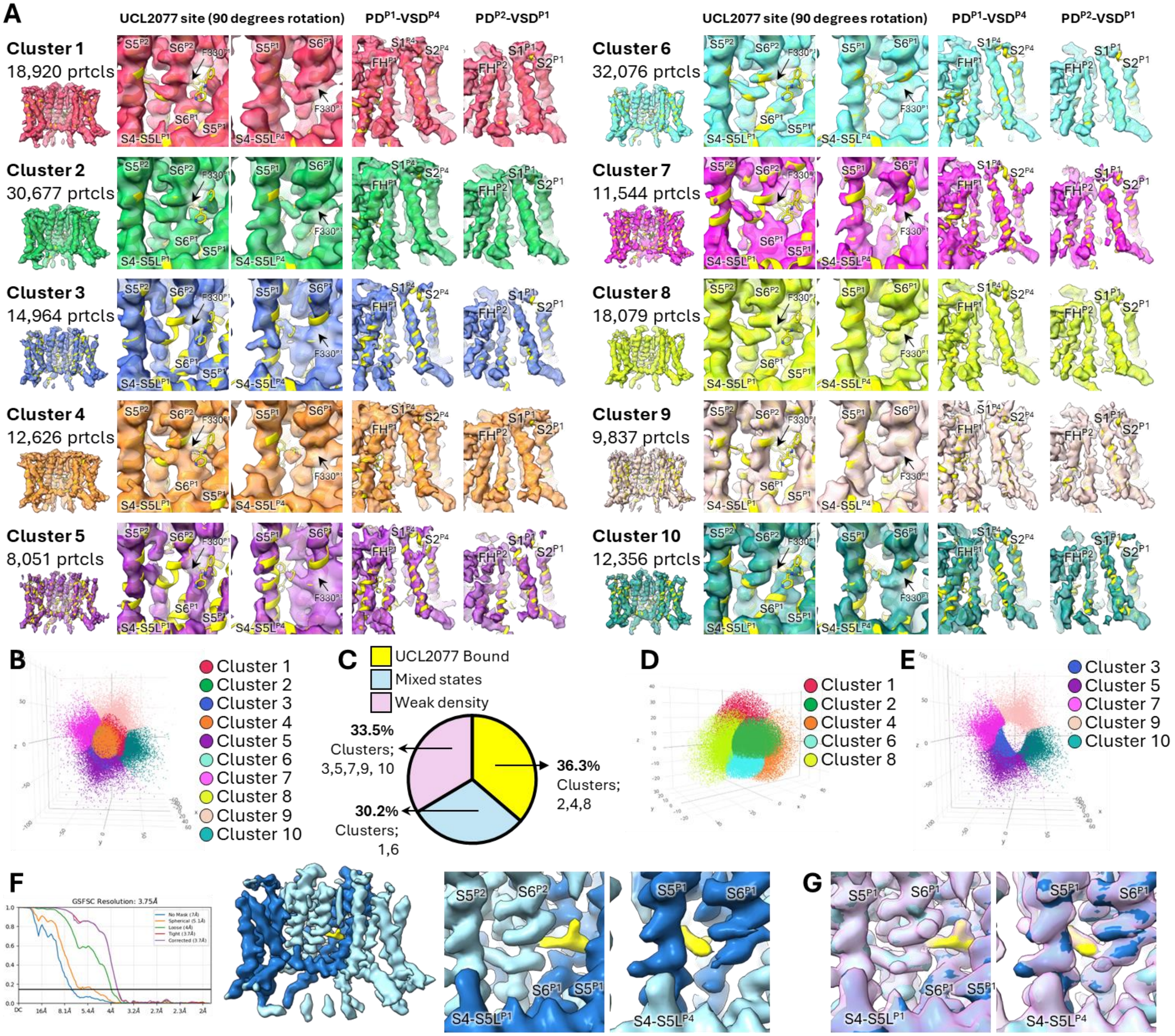

Fig. S19. xWT-UCL2077 dataset 3DVA. (**A**) 3DVA clusters showing, from left to right, the dimer and gate conformations, the UCL2077 binding site (including a ~90° rotated view), and the VSD-PD packing arrangement. (**B**) Clustered scatter plots of the 3DVA particle distribution. (**C**) Pie chart summarizing the proportion of particles assigned to the different conformational states. (**D**) Clustered scatter plots corresponding to subclasses yielding high-quality reconstructions. (**E**) Clustered scatter plots corresponding to subclasses yielding lower-quality reconstructions. (**F**) FSC curves, cryo-EM maps, and close-up views of the UCL2077 binding site from homogeneous reconstructions obtained from clusters 2, 4, and 8. (**G**) Comparison between the local map derived from the homogeneous reconstruction and the C2-NU refinement, shown as a transparent pink surface.

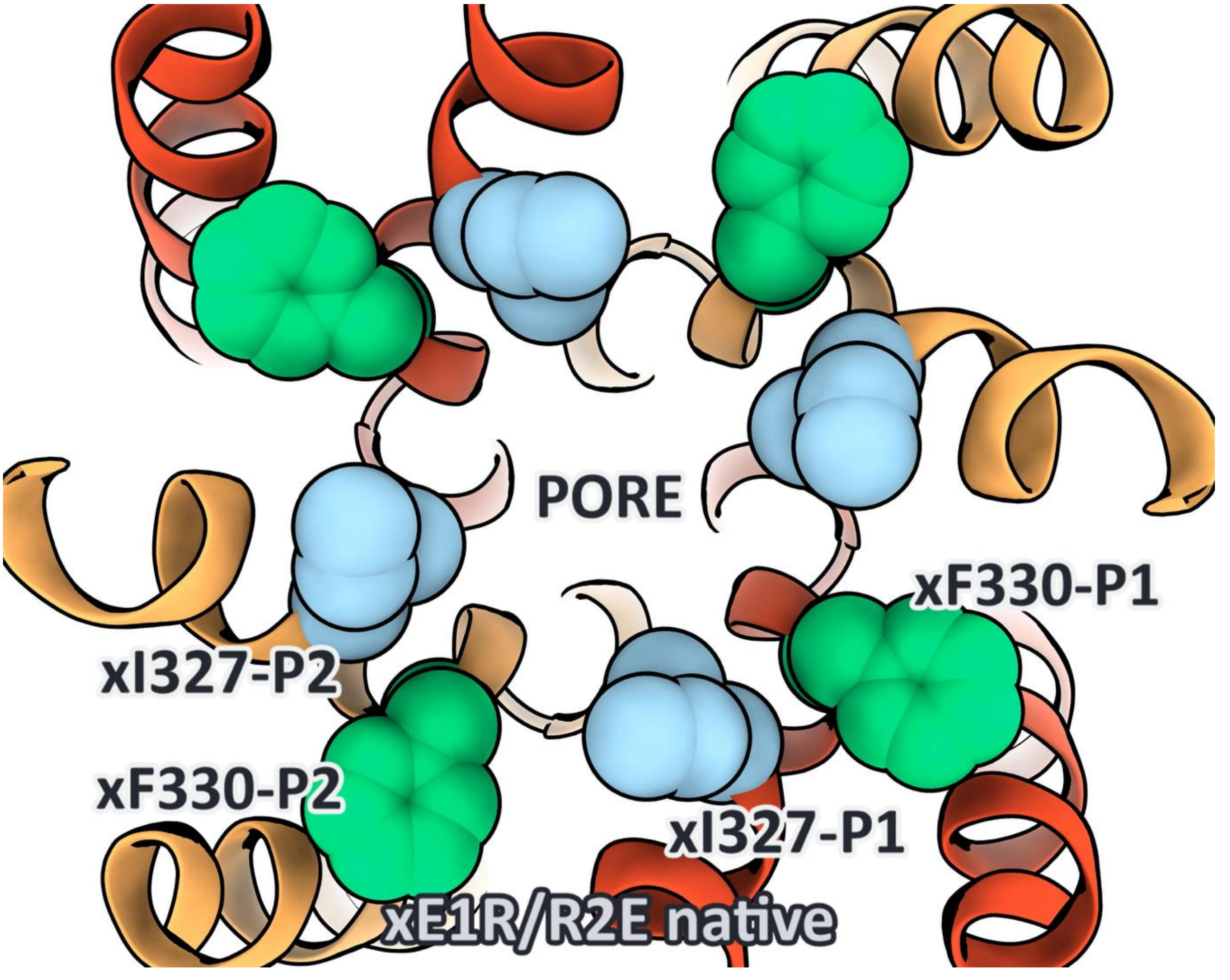

Movie S1. Morph between the native and UCL2077-bound structures shown in top view, clipped at the UCL2077 level. UCL2077-bound PDs are colored red, while UCL2077-free PDs are colored orange. Residues xF330 and xI327 in the S6 helix are shown as green and blue spheres, respectively;. UCL2077 is depicted as yellow sticks.

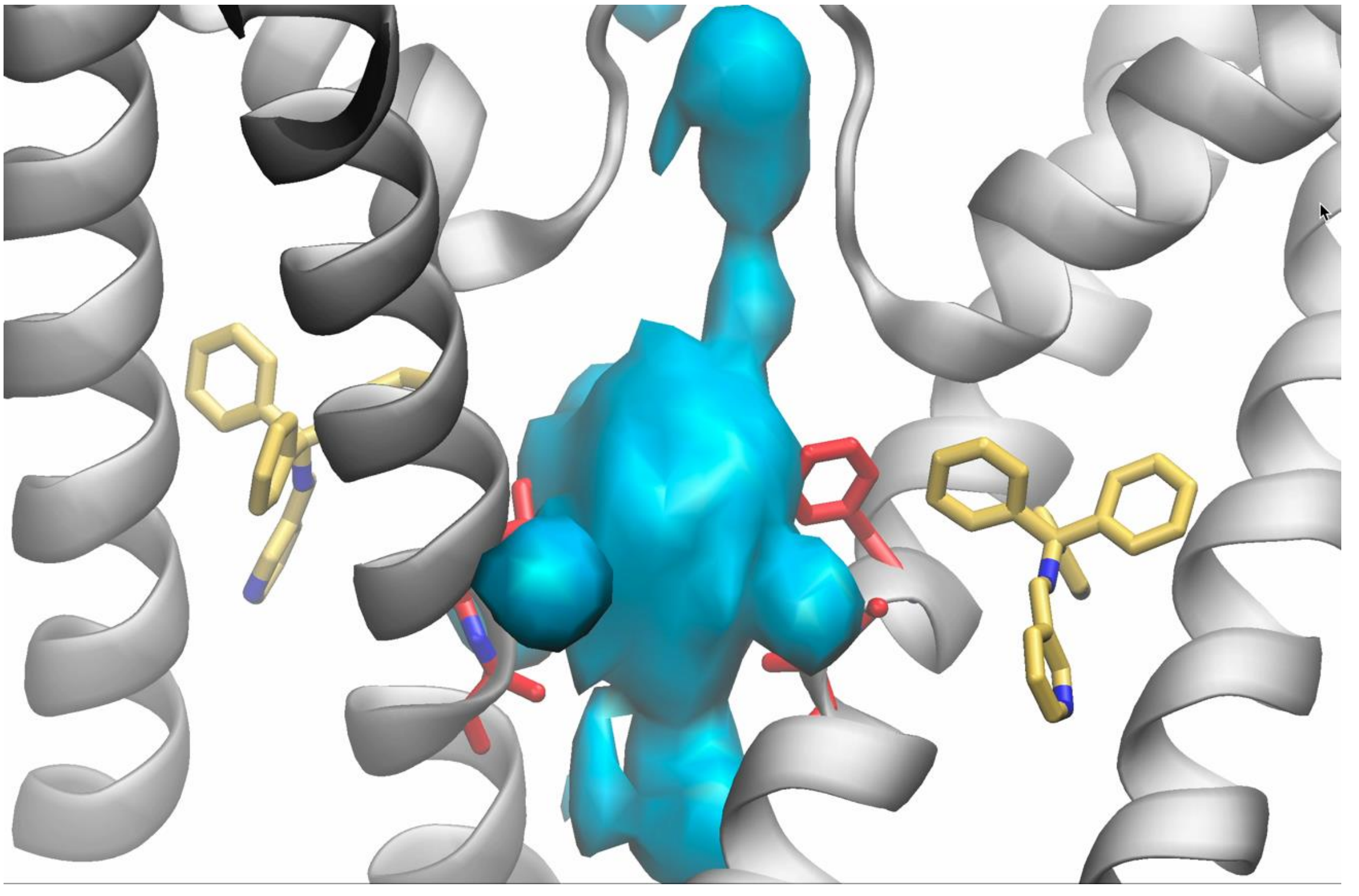

Movie S2. Dehydration of the water pore column. Pore dehydration in the xE1R/R2E structure caused by the diagonal xF330 residues approaching.

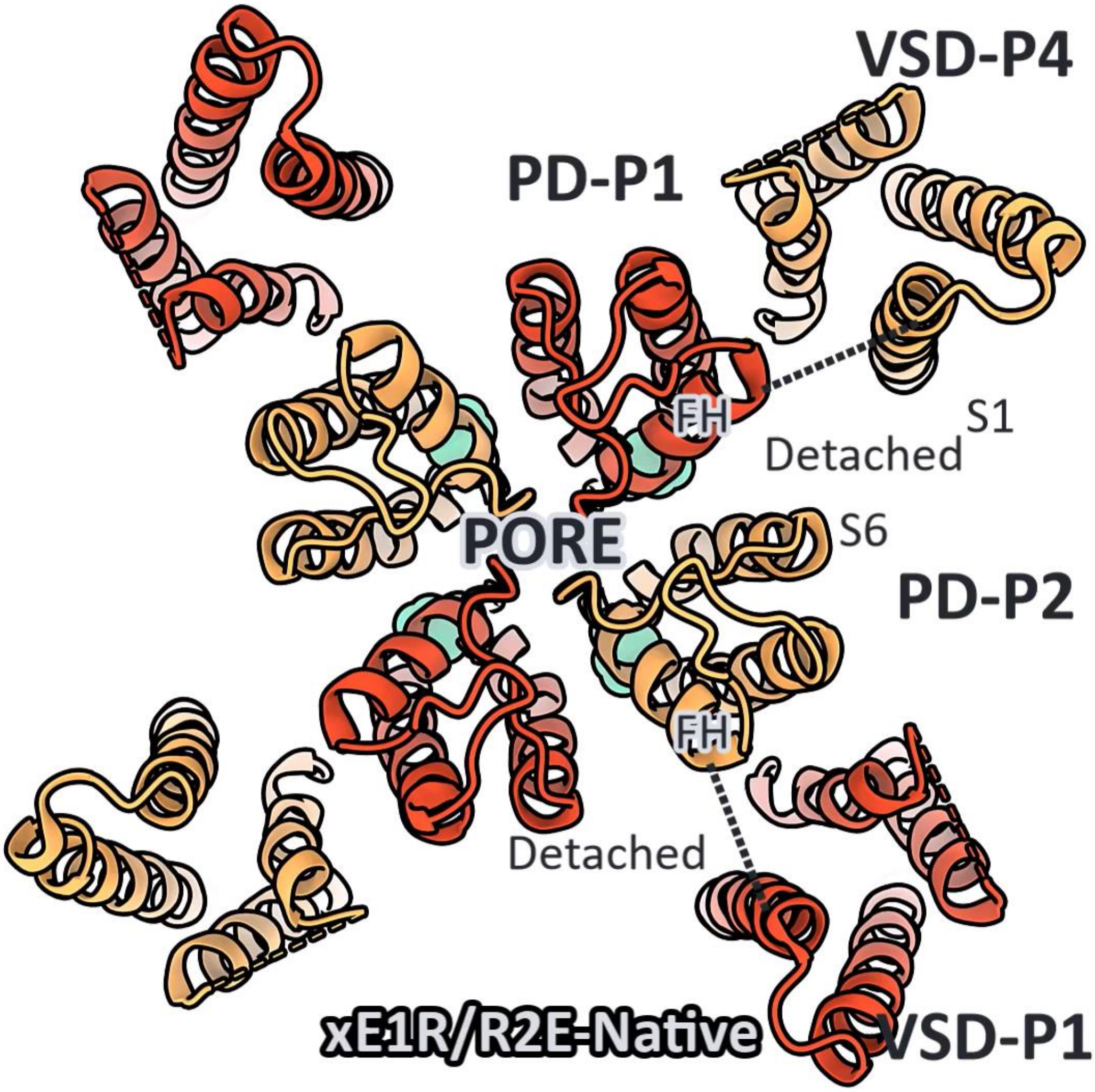

Movie S3. Morph between the native xE1R/R2E and UCL2077-bound structures in top view, focused on the domain-swapped pairs (VSD-P4)/(PD-P1) and (PD-P2)/(VSD-P1). UCL2077-bound PDs are colored red, while UCL2077-free PDs are colored orange. xF330 and UCL2077 are shown as green and yellow spheres, respectively. The attached/detached state of the VSD-PD is also indicated.

Table S1. Cryo-EM data collection, refinement and validation statistics		
	**xE1R/R2E-CaM-UCL2077**<br>**EMD-75305**<br>**PDB 10NK**	**xWT-CaM-UCL2077**<br>**EMD-75306**<br>**PDB 10NL**
**Data collection and processing**		
**Magnification**	130,000	130,000
**Voltage (kV)**	300	300
**Electron exposure (e–/Å^2)**	50	50
**Defocus range (μm)**	-0.5 to -2.0	-0.5 to -2.0
**Pixel size (Å)**	0.96	0.96
**Symmetry imposed**	C2	C2
**Initial particle images (no.)**	1,054,615	687,138
**Final particle images (no.)**	57,405	84,565
**Map resolution (Å)**<br>**FSC threshold**	3.95<br>0.143	3.81<br>0.143
**Map resolution range (Å)**	3.2-8.0	2.9-8.0
**Refinement**		
**Initial model used (PDB code)**	9MY4	9MY3
**Model resolution (Å)**<br>**FSC threshold**	4.0<br>0.5	4.0<br>0.5
**Model resolution range (Å)**	3.8-4.1	3.6-4.0
**Model composition**<br>**Non-hydrogen atoms**<br>**Protein residues**<br>**Ligands**	 14,516<br>1,880<br>2	 14,844<br>1888<br>2
**B factors (Å^2)**<br>**Protein**<br>**Ligand**	 178.38<br>95.40	 180.48<br>98.09
**R.m.s. deviations**<br>**Bond lengths (Å)**<br>**Bond angles (°)**	 0.003<br>0.888	 0.003<br>0.835
**Validation**<br>**MolProbity score**<br>**Clashscore**<br>**Poor rotamers (%)**	 2.05<br>13.84<br>1.82	 1.79<br>9.85<br>0.27
**Ramachandran plot**<br>**Favored (%)**<br>**Allowed (%)**<br>**Disallowed (%)**	 0.0<br>3.25<br>96.75	 0.0<br>3.99<br>96.01

Table S2. Simulations summary. Simulations marked with * were performed in Kyriakis et al.(20)

System	Protein residues	UCL2077	Restrained equilibration	Trajectory length	Replicas
KCNQ1					
xE1R/R2E	N95-Q349	EMERALD pose #1	530 ns	500 ns	3
xE1R/R2E	G235-Q349	EMERALD pose #1	30 ns	1 µs	3
xE1R/R2E	G235-Q349	EMERALD pose #2	30 ns	1 µs	3
xE1R/R2E	G235-Q349	EMERALD pose #3	30 ns	1 µs	3
xE1R/R2E-native*	N95-Q349	Not present	30 ns	500 ns	3
xWT	N95-Q349	Not present	30 ns	500 ns	3
xE1R/R2E native	N95-Q349	Docked	30 ns	500 ns	3

REFERENCES

1. C. C. Shieh, M. Coghlan, J. P. Sullivan, M. Gopalakrishnan, Potassium channels: Molecular defects, diseases, and therapeutic opportunities. *Pharmacol. Rev.* **52**, 557–593 (2000).

2. M. C. Sanguinetti, M. E. Curran, A. Zou, J. Shen, P. S. Specter, D. L. Atkinson, M. T. Keating, Coassembly of KVLQT1 and minK (IsK) proteins to form cardiac IKS potassium channel. *Nature* **384**, 80–83 (1996).

3. J. Barhanin, F. Lesage, E. Guillemare, M. Fink, M. Lazdunski, G. Romey, KvLQT1 and IsK (minK) proteins associate to form the IKS cardiac potassium current. *Nature* **384**, 78–80 (1996).

4. B. C. Schroeder, S. Waldegger, S. Fehr, M. Bleich, R. Warth, R. Greger, T. J. Jentsch, A constitutively open potassium channel formed by KCNQ1 and KCNE3. *Nature* **403**, 196–199 (2000).

5. S. I. Liin, R. Barro-Soria, H. P. Larsson, The KCNQ1 channel—Remarkable flexibility in gating allows for functional versatility. *J. Physiol.* **593**, 2605–2615 (2015).

6. H. Hibino, F. Nin, C. Tsuzuki, Y. Kurachi, How is the highly positive endocochlear potential formed? The specific architecture of the stria vascularis and the roles of the ion-transport apparatus. *Pflugers Arch.* **459**, 521–533 (2010).

7. D. Fedida, Vernakalant (RSD1235): A novel, atrial-selective antifibrillatory agent. *Expert Opin. Investig. Drugs* **16**, 519–532 (2007).

8. L. Shamgar, L. Ma, N. Schmitt, Y. Haitin, A. Peretz, R. Wiener, J. Hirsch, O. Pongs, B. Attali, Calmodulin is essential for cardiac I_{KS} channel gating and assembly: Impaired function in long-QT mutations. *Circ. Res.* **98**, 1055–1063 (2006).

9. J. Sun, R. MacKinnon, Cryo-EM structure of a KCNQ1/CaM complex reveals insights into congenital long QT syndrome. *Cell* **169**, 1042–1050.e9 (2017).

10. J. Sun, R. MacKinnon, Structural basis of human KCNQ1 modulation and gating. *Cell* **180**, 340–347.e9 (2020).

11. K. Willegems, J. Eldstrom, E. Kyriakis, F. Ataei, H. Sahakyan, Y. Dou, S. Russo, F. Van Petegem, D. Fedida, Structural and electrophysiological basis for the modulation of KCNQ1 channel currents by ML277. *Nat. Commun.* **13**, 3760 (2022).

12. H. Zhang, L. C. Craciun, T. Mirshahi, T. Rohács, C. M. B. Lopes, T. Jin, D. E. Logothetis, PIP2 activates KCNQ channels, and its hydrolysis underlies receptor-mediated inhibition of M currents. *Neuron* **37**, 963–975 (2003).

13. M. A. Zaydman, J. R. Silva, K. Delaloye, Y. Li, H. Liang, H. P. Larsson, J. Shi, J. Cui, Kv7.1 ion channels require a lipid to couple voltage sensing to pore opening. *Proc. Natl. Acad. Sci. U.S.A.* **110**, 13180–13185 (2013).

14. P. Hou, P. W. Kang, A. D. Kongmeneck, N.-D. Yang, Y. Liu, J. Shi, X. Xu, K. M. White, M. A. Zaydman, M. A. Kasimova, G. Seebohm, L. Zhong, X. Zou, M. Tarek, J. Cui, Two-stage electro–mechanical coupling of a KV channel in voltage-dependent activation. *Nat. Commun.* **11**, 676 (2020).

15. V. S. Mandala, R. MacKinnon, The membrane electric field regulates the PIP_2–binding site to gate the KCNQ1 channel. *Proc. Natl. Acad. Sci. U.S.A.* **120**, e2301985120 (2023).

16. P. Hou, J. Eldstrom, J. Shi, L. Zhong, K. McFarland, Y. Gao, D. Fedida, J. Cui, Inactivation of KCNQ1 potassium channels reveals dynamic coupling between voltage sensing and pore opening. *Nat. Commun.* **8**, 1730 (2017).

17. M. A. Zaydman, M. A. Kasimova, K. McFarland, Z. Beller, P. Hou, H. E. Kinser, H. Liang, G. Zhang, J. Shi, M. Tarek, J. Cui, Domain–domain interactions determine the gating, permeation, pharmacology, and subunit modulation of the IKs ion channel. *eLife* **3**, e03606 (2014).

18. D. Wu, K. Delaloye, M. A. Zaydman, A. Nekouzadeh, Y. Rudy, J. Cui, State-dependent electrostatic interactions of S4 arginines with E1 in S2 during Kv7.1 activation. *J. Gen. Physiol.* **135**, 595–606 (2010).

19. D. Sastre, E. Kyriakis, J. Schauer, M. Chan, Y. Dou, J. Eldstrom, D. Fedida, Electrophysiological characterization of the state-dependent inhibition of Kv7.1 and IKs by UCL2077. *Mol. Pharmacol.* **107**, 100076 (2025).

20. E. Kyriakis, D. Sastre, J. Eldstrom, A. Roscioni, S. Russo, F. Ataei, Y. Dou, M. Chan, S. Molinarolo, L. Maragliano, F. Van Petegem, D. Fedida, A physiologically-relevant intermediate state structure of a voltage-gated potassium channel. *Nat. Commun.* **16**, 8814 (2025).

21. H. Soh, A. V. Tzingounis, The specific slow afterhyperpolarization inhibitor ucl2077 is a subtype-selective blocker of the epilepsy associated KCNQ channels. *Mol. Pharmacol.* **78**, 1088–1095 (2010).

22. M. M. Shah, M. Javadzadeh-Tabatabaie, D. C. H. Benton, C. R. Ganellin, D. G. Haylett, Enhancement of hippocampal pyramidal cell excitability by the novel selective slow-afterhyperpolarization channel blocker 3-(Triphenylmethylaminomethyl)pyridine (UCL2077). *Mol. Pharmacol.* **70**, 1494–1502 (2006).

23. R. Barro-Soria, S. Rebolledo, S. I. Liin, M. E. Perez, K. J. Sampson, R. S. Kass, H. P. Larsson, KCNE1 divides the voltage sensor movement in KCNQ1/KCNE1 channels into two steps. *Nat. Commun.* **5**, 3750 (2014).

24. L.-J. Ma, I. Ohmert, V. Vardanyan, Allosteric Features of KCNQ1 Gating Revealed by Alanine Scanning Mutagenesis. *Biophys. J.* **100**, 885–894 (2011).

25. L. Ma, “Mutational analysis of KCNQ1 (Kv 7.1) channel gating,” thesis, University of Hamburg, Hamburg (2007).

26. A. Muenks, S. Zepeda, G. Zhou, D. Veesler, F. DiMaio, Automatic and accurate ligand structure determination guided by cryo-electron microscopy maps. *Nat. Commun.* **14**, 1164 (2023).

27. O. Trott, A. J. Olson, AutoDock Vina: Improving the speed and accuracy of docking with a new scoring function, efficient optimization, and multithreading. *J. Comput. Chem.* **31**, 455–461 (2010).

28. L. Zhong, X. Lin, X. Cheng, S. Wan, Y. Hua, W. Nan, B. Hu, X. Peng, Z. Zhou, Q. Zhang, H. Yang, F. Noé, Z. Yan, D. Jiang, H. Zhang, F. Liu, C. Xiao, Z. Zhou, Y. Mou, H. Yu, L. Ma, C. Huang, V. K. W. Wong, S. K. Chung, B. Shen, Z.-H. Jiang, E. Neher, W. Zhu, J. Zhang, P. Hou, Secondary structure transitions and dual PIP2 binding define cardiac KCNQ1-KCNE1 channel gating. *Cell Res.* **35**, 887–899 (2025).

29. C. Cui, L. Zhao, A. A. Kermani, S. Du, T. Pipatpolkai, M. Jiang, S. Chittori, Y. Z. Tan, J. Shi, L. Delemotte, J. Cui, J. Sun, Mechanisms of KCNQ1 gating modulation by KCNE1/3 for cell-specific function. *Cell Res.* **35**, 876–886 (2025).

30. J. Li, Z. Yang, S. Zhang, Y. Ye, J. He, Y. Zhang, H. Han, W. Kong, J. Liu, Y. Min, J. Shen, L. Mei, Z. Chen, P. Hou, J. Guo, Q. Zhang, H. Yang, Small molecule inhibits KCNQ channels with a non-blocking mechanism. *Nat. Chem. Biol.* **21**, 1100–1109 (2025).

31. T. Li, K. Wu, Z. Yue, Y. Wang, F. Zhang, H. Shen, Structural basis for the modulation of human KCNQ4 by small-molecule drugs. *Mol. Cell* **81**, 25–37.e4 (2021).

32. J. Robbins, KCNQ potassium channels: Physiology, pathophysiology, and pharmacology. *Pharmacol. Ther.* **90**, 1–19 (2001).

33. G. Peng, R. Barro-Soria, K. J. Sampson, H. P. Larsson, R. S. Kass, Gating mechanisms underlying deactivation slowing by two KCNQ1 atrial fibrillation mutations. *Sci. Rep.* **7**, 45911 (2017).

34. E. Tao, B. Corry, Characterizing fenestration size in sodium channel subtypes and their accessibility to inhibitors. *Biophys. J.* **121**, 193–206 (2022).

35. N.-D. Yang, R. Kanyo, L. Zhao, J. Li, P. W. Kang, A. K. Dou, K. M. White, J. Shi, J. M. Nerbonne, H. T. Kurata, J. Cui, Electro-mechanical coupling of KCNQ channels is a target of epilepsy-associated mutations and retigabine. *Sci. Adv.* **8**, eabo3625 (2022).

36. M. Nappi, D. J. A. Frampton, A. S. Kusay, K. Wang, S. S. Yasarbas, S. Pozzi, F. Miceli, S. I. Liin, M. Taglialatela, A. Pantazis, Two-step voltage-sensor activation of the human KV7.4 channel and effect of a deafness-associated mutation. *Nat. Commun.* **17**, 2381 (2026).

37. A. Abrahamyan, J. Eldstrom, H. Sahakyan, N. Karagulyan, L. Mkrtchyan, T. Karapetyan, E. Sargsyan, M. Kneussel, K. Nazaryan, J. R. Schwarz, D. Fedida, V. Vardanyan, Mechanism of external K+ sensitivity of KCNQ1 channels. *J. Gen. Physiol.* **155**, e202213205 (2023).

38. Y. Liu, C. Bassetto, G. F. Contreras, E. Perozo, F. Bezanilla, Closed state structure of the pore revealed by uncoupled Shaker K+ channel. *Nat. Commun.* **16**, 10180 (2025).

39. G. A. Haddad, R. Blunck, Mode shift of the voltage sensors in Shaker K+ channels is caused by energetic coupling to the pore domain. *J. Gen. Physiol.* **137**, 455–472 (2011).

40. T. D. Goddard, C. C. Huang, E. C. Meng, E. F. Pettersen, G. S. Couch, J. H. Morris, T. E. Ferrin, UCSF ChimeraX: Meeting modern challenges in visualization and analysis. *Protein Sci.* **27**, 14–25 (2018).

41. A. Punjani, J. L. Rubinstein, D. J. Fleet, M. A. Brubaker, cryoSPARC: Algorithms for rapid unsupervised cryo-EM structure determination. *Nat. Methods* **14**, 290–296 (2017).

42. P. Emsley, B. Lohkamp, W. G. Scott, K. Cowtan, Features and development of *Coot*. *Acta Crystallogr. D Biol. Crystallogr.* **66**, 486–501 (2010).

43. D. Liebschner, P. V. Afonine, M. L. Baker, G. Bunkóczi, V. B. Chen, T. I. Croll, B. Hintze, L.-W. Hung, S. Jain, A. J. McCoy, N. W. Moriarty, R. D. Oeffner, B. K. Poon, M. G. Prisant, R. J. Read, J. S. Richardson, D. C. Richardson, M. D. Sammito, O. V. Sobolev, D. H. Stockwell, T. C. Terwilliger, A. G. Urzhumtsev, L. L. Videau, C. J. Williams, P. D. Adams, Macromolecular structure determination using X-rays, neutrons and electrons: Recent developments in *Phenix*. *Acta Crystallogr. Sect. Struct. Biol.* **75**, 861–877 (2019).

44. C. J. Williams, J. J. Headd, N. W. Moriarty, M. G. Prisant, L. L. Videau, L. N. Deis, V. Verma, D. A. Keedy, B. J. Hintze, V. B. Chen, S. Jain, S. M. Lewis, W. B. Arendall, J. Snoeyink, P. D. Adams, S. C. Lovell, J. S. Richardson, D. C. Richardson, MolProbity: More and better reference data for improved all-atom structure validation. *Protein Sci.* **27**, 293–315 (2018).

45. S. Jo, T. Kim, W. Im, Automated builder and database of protein/membrane complexes for molecular dynamics simulations. *PLOS ONE* **2**, e880 (2007).

46. G. Alberini, S. Alexis Paz, B. Corradi, C. F. Abrams, F. Benfenati, L. Maragliano, Molecular dynamics simulations of ion permeation in human voltage-gated sodium channels. *J. Chem. Theory Comput.* **19**, 2953–2972 (2023).

47. M. Nappi, G. Alberini, A. Berselli, A. Roscioni, M. V. Soldovieri, I. Servettini, V. Barrese, S. Weckhuysen, T.-G. A. Chiu, I. E. Scheffer, F. Benfenati, L. Maragliano, F. Miceli, M. Taglialatela, Constitutive opening of the Kv7.2 pore activation gate causes *KCNQ2* -developmental encephalopathy. *Proc. Natl. Acad. Sci. U.S.A.* **121**, e2412388121 (2024).

48. S. Bienert, A. Waterhouse, T. A. P. de Beer, G. Tauriello, G. Studer, L. Bordoli, T. Schwede, The SWISS-MODEL Repository—new features and functionality. *Nucleic Acids Res.* **45**, D313–D319 (2017).

49. J. C. Phillips, R. Braun, W. Wang, J. Gumbart, E. Tajkhorshid, E. Villa, C. Chipot, R. D. Skeel, L. Kalé, K. Schulten, Scalable molecular dynamics with NAMD. *J. Comput. Chem.* **26**, 1781–1802 (2005).

50. J. Huang, S. Rauscher, G. Nawrocki, T. Ran, M. Feig, B. L. De Groot, H. Grubmüller, A. D. MacKerell, CHARMM36m: An improved force field for folded and intrinsically disordered proteins. *Nat. Methods* **14**, 71–73 (2017).

51. W. L. Jorgensen, J. Chandrasekhar, J. D. Madura, R. W. Impey, M. L. Klein, Comparison of simple potential functions for simulating liquid water. *J. Chem. Phys.* **79**, 926–935 (1983).

52. K. Vanommeslaeghe, E. Hatcher, C. Acharya, S. Kundu, S. Zhong, J. Shim, E. Darian, O. Guvench, P. Lopes, I. Vorobyov, A. D. Mackerell, CHARMM general force field: A force field for drug-like molecules compatible with the CHARMM all-atom additive biological force fields. *J. Comput. Chem.* **31**, 671–690 (2010).

53. P. J. Steinbach, B. R. Brooks, New spherical-cutoff methods for long-range forces in macromolecular simulation. *J. Comput. Chem.* **15**, 667–683 (1994).

54. T. Darden, D. York, L. Pedersen, Particle mesh Ewald: An N log(N) method for Ewald sums in large systems. *J. Chem. Phys.* **98**, 10089–10092 (1993).

55. J.-P. Ryckaert, G. Ciccotti, H. J. C. Berendsen, Numerical integration of the cartesian equations of motion of a system with constraints: Molecular dynamics of n-alkanes. *J. Comput. Phys.* **23**, 327–341 (1977).

56. S. Miyamoto, P. A. Kollman, Settle: An analytical version of the SHAKE and RATTLE algorithm for rigid water models. *J. Comput. Chem.* **13**, 952–962 (1992).

57. G. J. Martyna, D. J. Tobias, M. L. Klein, Constant pressure molecular dynamics algorithms. *J. Chem. Phys.* **101**, 4177–4189 (1994).

58. S. E. Feller, Y. Zhang, R. W. Pastor, B. R. Brooks, Constant pressure molecular dynamics simulation: The Langevin piston method. *J. Chem. Phys.* **103**, 4613–4621 (1995).

59. J. Eberhardt, D. Santos-Martins, A. F. Tillack, S. Forli, AutoDock Vina 1.2.0: New docking methods, expanded force field, and Python bindings. *J. Chem. Inf. Model.* **61**, 3891–3898 (2021).

60. X. Daura, K. Gademann, B. Jaun, D. Seebach, W. F. Van Gunsteren, A. E. Mark, Peptide folding: When SIMULATION MEETS EXPERIMENT. *Angew. Chem. Int. Ed. Engl.* **38**, 236–240 (1999).

61. O. S. Smart, J. G. Neduvelil, X. Wang, B. A. Wallace, M. S. P. Sansom, HOLE: A program for the analysis of the pore dimensions of ion channel structural models. *J. Mol. Graph.* **14**, 354–360 (1996).

62. O. S. Smart, J. Breed, G. R. Smith, M. S. Sansom, A novel method for structure-based prediction of ion channel conductance properties. *Biophys. J.* **72**, 1109–1126 (1997).

63. W. Humphrey, A. Dalke, K. Schulten, VMD: Visual molecular dynamics. *J. Mol. Graph.* **14**, 33–38 (1996).

64. G. Fiorin, M. L. Klein, J. Hénin, Using collective variables to drive molecular dynamics simulations. *Mol. Phys.* **111**, 3345–3362 (2013).

65. C. Bouysset, S. Fiorucci, ProLIF: A library to encode molecular interactions as fingerprints. *J. Cheminform.* **13**, 72 (2021).

66. R. Gowers, M. Linke, J. Barnoud, T. Reddy, M. Melo, S. Seyler, J. Domański, D. Dotson, S. Buchoux, I. Kenney, O. Beckstein, "MDAnalysis: A Python Package for the Rapid Analysis of Molecular Dynamics Simulations" in *Proceedings of the 15th Python in Science Conference,* Austin, TX, 2016 (SciPy, 2016), pp. 98–105; https://doi.curvenote.com/10.25080/Majora-629e541a-00e.

67. X. Li, Q. Zhang, P. Guo, J. Fu, L. Mei, D. Lv, J. Wang, D. Lai, S. Ye, H. Yang, J. Guo, Molecular basis for ligand activation of the human KCNQ2 channel. *Cell Res.* **31**, 52–61 (2021).

68. A. Punjani, D. J. Fleet, 3D variability analysis: Resolving continuous flexibility and discrete heterogeneity from single particle cryo-EM. *J. Struct. Biol.* **213**, 107702 (2021).

69. R. Sanchez-Garcia, J. Gomez-Blanco, A. Cuervo, J. M. Carazo, C. O. S. Sorzano, J. Vargas, DeepEMhancer: A deep learning solution for cryo-EM volume post-processing. *Commun. Biol.* **4**, 874 (2021).

70. H. Cao, Y. Zhu, T. Li, J. Chen, J. He, X. Wang, S.-Y. Huang, EMReady2: Improvement of cryo-EM and cryo-ET maps by local quality-aware deep learning with Mamba. *Nat. Commun.* **17**, 6493 (2026).
